# Whole Genome In-Silico Analysis of South African G1P[8] Rotavirus Strains before and after Vaccine Introduction over a Period of 14 Years

**DOI:** 10.3390/vaccines8040609

**Published:** 2020-10-14

**Authors:** Peter N. Mwangi, Milton T. Mogotsi, Mapaseka L. Seheri, M. Jeffrey Mphahlele, Ina Peenze, Mathew D. Esona, Benjamin Kumwenda, A. Duncan Steele, Carl D. Kirkwood, Valantine N. Ndze, Francis E. Dennis, Khuzwayo C. Jere, Martin M. Nyaga

**Affiliations:** 1 Next Generation Sequencing Unit and Division of Virology, Faculty of Health Sciences, University of the Free State, Bloemfontein 9300, South Africa; nthigapete@gmail.com (P.N.M.); tmogotsi16@gmail.com (M.T.M.); 2 Diarrheal Pathogens Research Unit, Sefako Makgatho Health Sciences University, Medunsa 0204, South Africa; mapaseka.seheri@smu.ac.za (M.L.S.); Jeffrey.Mphahlele@mrc.ac.za (M.J.M.); ina.peenze@smu.ac.za (I.P.); mathew.esona@gmail.com (M.D.E.); 3 South African Medical Research Council, Pretoria 0001, South Africa; 4 College of Medicine, Department of Biomedical Sciences, Faculty of Biomedical Sciences and Health Professions, University of Malawi, Private Bag 360, Chichiri, Blantyre 3, Malawi; bkumwenda@medcol.mw; 5 Enteric and Diarrheal Diseases, Global Health, Bill & Melinda Gates Foundation, P.O. Box 23350, Seattle, WA 98109, USA; Duncan.Steele@gatesfoundation.org (A.D.S.); Carl.Kirkwood@gatesfoundation.org (C.D.K.); 6 Faculty of Health Sciences, University of Buea, P.O. Box 63, Buea, Cameroon; valentinengum@yahoo.com; 7 Noguchi Memorial Institute for Medical Research, University of Ghana, P.O. Box LG581, Legon, Ghana; fdennis@noguchi.ug.edu.gh; 8 Center for Global Vaccine Research, Institute of Infection, Liverpool L697BE, UK; Khuzwayo.Jere@liverpool.ac.uk; 9 Veterinary and Ecological Sciences, University of Liverpool, Liverpool L697BE, UK; 10 Malawi-Liverpool-Wellcome Trust Clinical Research Program, Department of Medical Laboratory Sciences, College of Medicine, University of Malawi, Blantyre 312225, Malawi

**Keywords:** evolution, rotavirus strains, Wa-like constellation, whole-genome, lineages

## Abstract

Rotavirus G1P[8] strains account for more than half of the group A rotavirus (RVA) infections in children under five years of age, globally. A total of 103 stool samples previously characterized as G1P[8] and collected seven years before and seven years after introducing the Rotarix^®^ vaccine in South Africa were processed for whole-genome sequencing. All the strains analyzed had a Wa-like constellation (G1-P[8]-I1-R1-C1-M1-A1-N1-T1-E1-H1). South African pre- and post-vaccine G1 strains were clustered in G1 lineage-I and II while the majority (84.2%) of the P[8] strains were grouped in P[8] lineage-III. Several amino acid sites across ten gene segments with the exception of VP7 were under positive selective pressure. Except for the N147D substitution in the antigenic site of eight post-vaccine G1 strains when compared to both Rotarix^®^ and pre-vaccine strains, most of the amino acid substitutions in the antigenic regions of post-vaccine G1P[8] strains were already present during the pre-vaccine period. Therefore, Rotarix^®^ did not appear to have an impact on the amino acid differences in the antigenic regions of South African post-vaccine G1P[8] strains. However, continued whole-genome surveillance of RVA strains to decipher genetic changes in the post-vaccine period remains imperative.

## 1. Introduction

Group A rotavirus (RVA) is the major causative agent of acute gastroenteritis (AGE) in children under five years [1]. RVA-induced diarrhea is responsible for approximately 125,000 childhood mortality cases worldwide [2]. RVA is classified in the *Reoviridae* family and comprises 11 dsRNA gene segments that encode six structural proteins (VP1-VP4, VP6, and VP7) and five/six non-structural proteins (NSP1-NSP5/6) [1]. A binary classification scheme based on the properties of the outer capsid proteins, VP7 and VP4, has been universally used to classify RVA strains [3]. However, to fully describe rotavirus strains, the binary classification system was expanded by incorporating the other nine genome segments [4].

In the whole-genome classification scheme, nucleotide percentage similarity cut-off values of all the eleven viral gene segments, as recommended by the Rotavirus Classification Working Group (RCWG), are used to determine a genotypic scheme Gx-P[x]-Ix-Rx-Cx-Mx-Ax-Nx-Tx-Ex-Hx designating VP7-VP4-VP6-VP1-VP2-VP3-NSP1-NSP2-NSP3-NSP4-NSP5/6, respectively [5]. The majority of RVA strains are assigned into three genogroups: Wa-like (I1-R1-C1-M1-A1-N1-T1-E1-H1), DS-1-like (I2-R2-C2-M2-A2-N2-T2-E2-H2), and a relatively minor group, AU-1-like (I3-R3-C3-M3-A3-N3-T3-E3-H3) [4]. G1P[8], G3P[8], G4P[8], G9P[8], and G12P[8] typically have the Wa-like genotype constellation, while G2P[4], G8P[4] and G8P[6] usually have the DS-1-like genotype constellation [6].

G1P[8] is among the most predominant and medically important RVA strain, globally [7]. In Africa, G1P[8] accounts for approximately 29% of all the circulating RVA strains [7]. The G1 and P[8] gene segments sub-cluster into lineages whose emergence is attributed to various mechanisms of genetic diversity common in RNA viruses such as genetic mutation, recombination, and reassortment [8]. Distinct lineages for both genotypes G1 and P[8] collected from different geographical regions have been described in the literature [9,10,11,12,13].

In order to alleviate RVA disease burden, four vaccines: RotaTeq^®^ (Merck & Co., West Point, PA, USA); Rotarix^®^ (GlaxoSmithKline, Rixenstart, Belgium); ROTAVAC^®^ (Bharat Biotech, Hyderabad, India); and Rotasiil^®^ (Serum Institute of India, Pune, India) have been pre-qualified by the World Health Organization (WHO) for global use [14]. Rotarix^®^ contains an attenuated human G1P[8] RVA strain [15], while RotaTeq^®^ is composed of five human-bovine reassortant strains (G1P[5], G2P[5], G3P[5], G4P[5], and G6P[8]) [16]. ROTAVAC^®^ contains a G9P[11] strain [17], while Rotasiil^®^ is a pentavalent human-bovine reassortant vaccine comprising five reassortant strains containing human VP7, representing the G1, G2, G3, G4 and G9 genotypes [18].

South Africa was the first African country to introduce the monovalent RVA vaccine, Rotarix^®^, into its Expanded Program on Immunization (EPI) in September 2009 [19]. In the first year, after the vaccine was introduced, RVA infections indicated by laboratory confirmed results and hospitalizations were reduced significantly by approximately 58% [20]. After the introduction of Rotarix^®^ in South Africa, the prevalence of non-G1P[8] strains (such as G2P[4], G2P[6], G9P[8], G12P[8] and G8P[4]) that were not incorporated in the monovalent G1P[8] vaccine increased significantly [21]. Notably, at Dr. George Mukhari Hospital, a key RVA surveillance site, no G1P[8] strains were reported in 2012 [21], whereas this was a predominant strain during the pre-vaccine period [22]. The enormous genetic and antigenic diversity within RVA [8,23] and the recent emergence of novel strains [24,25,26] emphasize the need to monitor the impact of RVA vaccines on the genetic and antigenic landscape of RVA circulating in the population.

The impact of RVA vaccination in Sub-Saharan Africa has been substantial [27] and it is essential to continuously assess the long-term impact of vaccination on circulating RVA strains. Thus, there is a need for whole-genome longitudinal surveillance studies in South Africa to decipher potential RVA vaccine-induced strain changes. In this study, we investigated the impact of RVA vaccine introduction in South Africa on the most common human RVA strain, G1P[8]. This study is the first large-scale genomic analysis of human RVA collected seven years before and seven years after the introduction of the RVA vaccine in South Africa.

## 2. Materials and Methods

### 2.1. Ethics Approval

The diarrheal stool samples were collected as a routine diagnostic clinical specimen when the parents brought their child to a health facility for clinical management, requiring no written informed consent. As part of the WHO-coordinated RVA surveillance network, the archived RVA-positive specimens were anonymized and utilized for strain characterization under a Technical Service Agreement and a Materials Transfer Agreement (MTA) to the WHO/AFRO Regional Reference Laboratory (WHO-RRL) based at Sefako Makgatho Health Sciences University (SMU), Pretoria, South Africa. The WHO Research Ethics Review Committee granted an exemption activity, noting that the study procedures were part of routine hospital-based RVA surveillance. The samples were transferred for whole-genome sequencing at the University of the Free State-Next Generation Sequencing (UFS-NGS) Unit through a MTA (SMU-UFS.1). The Health Sciences Research Ethics Committee (HSREC) of the UFS, Bloemfontein, South Africa, approved the study under ethics number UFS-HSD2018/0510/3107.

### 2.2. Strain Description

Rotavirus positive stool samples (*n* = 103) previously characterized as G1P[8] were sourced from the archival storage of the Diarrheal Pathogens Research Unit (DPRU), WHO-RRL in Pretoria, South Africa. The samples were distributed as follows: 2002 (*n* = 14), 2003 (*n* = 7), 2004 (*n* = 14), 2005 (*n* = 6), 2006 (*n* = 22), 2007 (*n* = 7), 2008 (*n* = 9), 2009 (*n* = 13), 2010 (*n* = 1), 2013 (*n* = 1), 2014 (*n* = 5), 2015 (*n* = 3), and 2017 (*n* = 1). In addition to the 103 samples, an additional 68 whole-genome sequences for G1P[8] strains collected from South Africa were extracted from the GenBank database [28].

### 2.3. Extraction and Purification of Double-Stranded RNA

A fecal suspension was prepared by adding approximately 100 mg stool sample into 200 µL Phosphate Buffered Saline (PBS) solution, 0.01 M, pH 7.2 (Sigma-Aldrich^®^, St Louis, MO, USA) with subsequent extraction of viral RNA as previously described [29] albeit with some modifications. Briefly, the modifications included the volume (900 µL TRIzol™-LS: 300 µL stool sample suspension), the incubation period of dsRNA enrichment (24 h), centrifugation speeds (20,000*× g*), and the staining reagent (PronaSafe, Condalab, Camberley, UK), as captured in the UFS-NGS unit extraction Standard Operating Procedure (SOP). The extracted RNA was purified using the MinElute PCR Purification Kit by following the manufacturers’ instructions (Qiagen, Hilden, Germany).

### 2.4. Complementary DNA (Cdna) Synthesis

cDNA was synthesized from the extracted viral RNA using the Maxima H Minus Double-Stranded Synthesis Kit and protocol (Thermo Fischer Scientific, Waltham, MA, USA) with some modifications (UFS-NGS Unit SOP). Briefly, for first-strand cDNA synthesis, a denaturing step of the purified extracted RNA at 95 °C for 5 min in Multigene Optimax thermocycler (Labnet, Edison, NJ, USA) was followed by the addition of 1 µL of Random Hexamer primer, 100 µM. The reaction mixture was incubated in a thermocycler at 65 °C for 5 min. Afterward, 5 µL volume of the 4× First-Strand Reaction Mix and 1 µL of First Strand Enzyme Mix was added and the reaction mixture was incubated in a thermocycler pre-programmed as follows: 10 min at 25 °C, 120 min at 50 °C, and 5 min at 85 °C. For the second-strand cDNA synthesis step, a 55 µL volume of Nuclease-Free water, 20 µL of 5× Second Strand Reaction Mix, and 5 µL of Second Strand Enzyme Mix was added and incubated in the thermal cycler at 16 °C for 60 min. A 6 µL volume of EDTA, pH 8.9 was added followed by 10 µL of RNase I. The synthesized cDNA was purified using MSB^®^ Spin PCRapace Kit by following the manufacturer’s protocol (Stratec Molecular, Berlin, Germany).

### 2.5. DNA Library Preparation and Whole-Genome Sequencing

The DNA libraries were prepared by utilizing the Nextera^®^ XT DNA Library Preparation Kit (Illumina, San Diego, CA, USA). Quantitative and qualitative assessment of DNA was performed using Qubit 3.0 fluorometer (Invitrogen, Carlsbad, CA, USA) and Agilent 2100 BioAnalyzer^®^ (Agilent Technologies, Waldbronn, Germany), respectively, by following the manufacturer’s instructions. The DNA library and the PhiX Control v3 library (Illumina, San Diego, CA, USA) were normalized to 8 pM and 20 pM concentrations, respectively. A volume of 600 µL pooled denatured DNA library spiked with 20% PhiX Control v3 library was loaded into a MiSeq^®^ Reagent Kit V3 for paired-end nucleotide sequencing (301 × 2) on a MiSeq^®^ sequencer (Illumina, San Diego, CA, USA) at the University of the Free State-Next Generation Sequencing (UFS-NGS) Unit, Bloemfontein, South Africa.

### 2.6. Genome Assembly

The trimming of Illumina read ends and subsequent genome assembly was performed using a suite of tools embedded in Geneious Prime^®^ software, version 2020.1.1 [30]. Complementary RVA assembly was also performed using an in-house developed genome assembly pipeline and CLC Genomics Workbench 12 (https://www.qiagenbioinformatics.com/).

### 2.7. Generation of Whole-Genome Constellations

The genotype of each gene segment was determined using the RVA determination tool in the Virus Pathogen Database and Analysis Resource (ViPR) [31] to generate the full genome constellations for each RVA strain.

### 2.8. Phylogenetic Analyses

Alignments and comparative analysis of the full-length sequences for each gene segment was performed as described previously [24]. Multiple sequence alignments were performed using the MAFFT package in Geneious Prime 2020 [30]. Duplicated sequences in the alignments were identified utilizing ElimDupes (https://www.hiv.lanl.gov/content/sequence/elimdupesv2/elimdupes.html). The best evolutionary models for each gene segment were estimated using the DNA Model Test program in MEGA 6 to guide in the construction of maximum-likelihood trees with 1000 bootstrap replicates [32].

### 2.9. Selection Pressure and Recombination Analysis

Analysis of natural selection in RVA genome segments was done using the suite from the DataMonkey Webserver [33]: Fixed-effects Likelihood (FEL) [34], Fast Unconstrained Bayesian Approximation for Inferring Selection (FUBAR) [35] and mixed-effects model of episodic selection (MEME) [36]. Amino acid sites undergoing positive selection were identified and tabulated. Analysis of genetic recombination was performed using Genetic Algorithm for Recombination Detection (GARD) [34].

### 2.10. Protein Modeling

The RVA protein structures were modeled using SWISS-MODEL with an initial template search [37]. The templates were selected from the SWISS-MODEL Template Library (SMTL) and their respective resolution values for the analyzed genes were as follows: VP7 (3 fmg.1, 3.40 Å) and VP4 (2 dwr.1, 2.50 Å). The evaluation of stereochemical quality parameters of the generated structures was performed using the Structure Assessment feature in the SWISS-MODEL server [37] and VERIFY3D [38]. Image visualization and analysis was performed in PyMol software [39].

### 2.11. In Silico Analysis of Effect of Mutation(s) on Protein Stability

The FoldX plugin [40] integrated in the YASARA platform [41] was used to predict the stability effect of mutation(s) in a 3D structure. FoldX estimates the stability effect of a mutation empirically whereby the stability (ΔG) of a protein is defined by the free energy, which is expressed in kcal/mol. In this study, G is the difference of free energy between the Rotarix^®^ (vaccine) strain or pre-vaccine strain and mutant strain. The energy with positive value is regarded to destabilize the structure, while a mutation with a negative value is regarded to stabilize the structure. Free energy change of ±0.5 kcal/moL is regarded as statistically significant for the stabilizing/destabilizing effect [40].

## 3. Results

### 3.1. Whole-Genome Constellation Determination

The genotype constellation for the 103 South African G1P[8] strains (92 pre- and 11 post-vaccine) sequenced in this study and corresponding 68 strains (56 pre- and 12 post-vaccine) acquired from the GenBank database as reference strains was typical G1-P[8]-I1-R1-C1-M1-A1-N1-T1-E1-H1 (Supplementary Materials 1 (S1)). The sizes of the full-length genome segments one to eleven and their respective open reading frames (ORFs) were determined (S1). All the gene sequences in this study were submitted in the NCBI GenBank database under accession numbers MT854335-MT855467.

### 3.2. Phylogenetic Analyses

#### 3.2.1. Phylogenetic Analyses of VP7 and VP4

**Figure 1:**
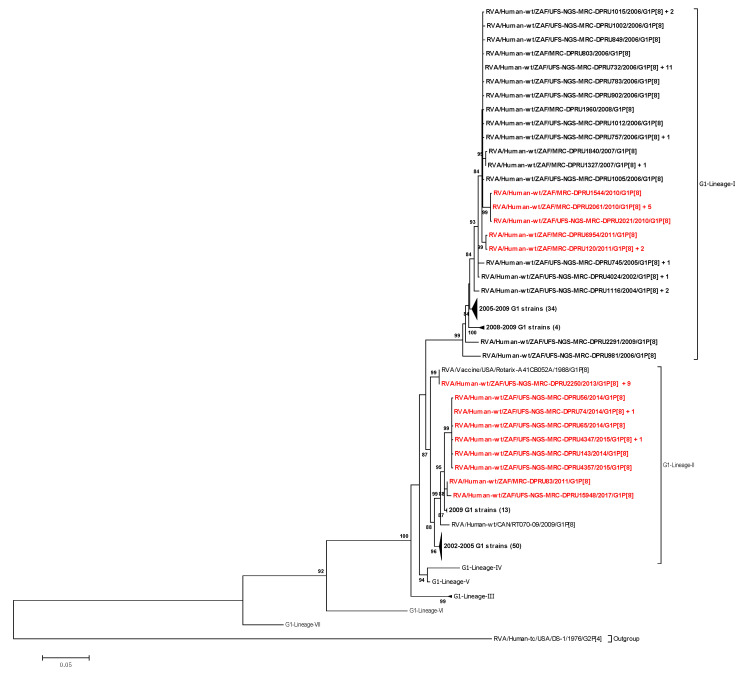
Maximum likelihood phylogenetic tree based on the full-length nucleotide sequences of genome segment 9 encoding the VP7 protein. The T92 + G evolutionary model was used for phylogenetic inference. South African pre-vaccine G1 strains are highlighted in bold-face while post-vaccine strains are highlighted in bold-red. Adjacent to some sequences is indicated a plus (+) sign followed by the number of identical sequences (S3). The number in brackets denotes the number of compressed strains. Lineages are indicated in roman numerals. Only bootstrap values ≥70% are shown adjacent to each branch node. Scale bar indicates the number of nucleotide substitutions per site.

Phylogenetic trees for each of the 11 gene segments were constructed. For VP7 and VP4, well-known lineage designations were utilized [9,11]. The VP7 phylogenetic tree comprised the VP7 gene sequences of the South African strains together with VP7 gene sequences of the reference strains from the seven established VP7 G1 genotype lineages [9]. The 171 G1 South African sequences utilized in this study were segregated into two main lineages: G1-lineage I and lineage II (Figure 1). G1-lineage I comprised 76 pre-vaccine G1 strains and 12 post-vaccine G1 strains, while G1-lineage II comprised 72 pre-vaccine G1 strains and 11 post-vaccine G1strains (Figure 1). Strain RVA/Human-wt/ZAF/UFS-NGS-MRC-DPRU2250/2013/G1P[8] had ≥99.9% nucleotide identity with Rotarix^®^ in all its structural and nonstructural genes (S2) and clustered alongside cognate Rotarix^®^ genes in all the 11 phylogenetic trees. South African pre- and post-vaccine G1 strains were highly identical to each other and similarly distant from the Rotarix^®^ strain (Table 1).

**Figure 2:**
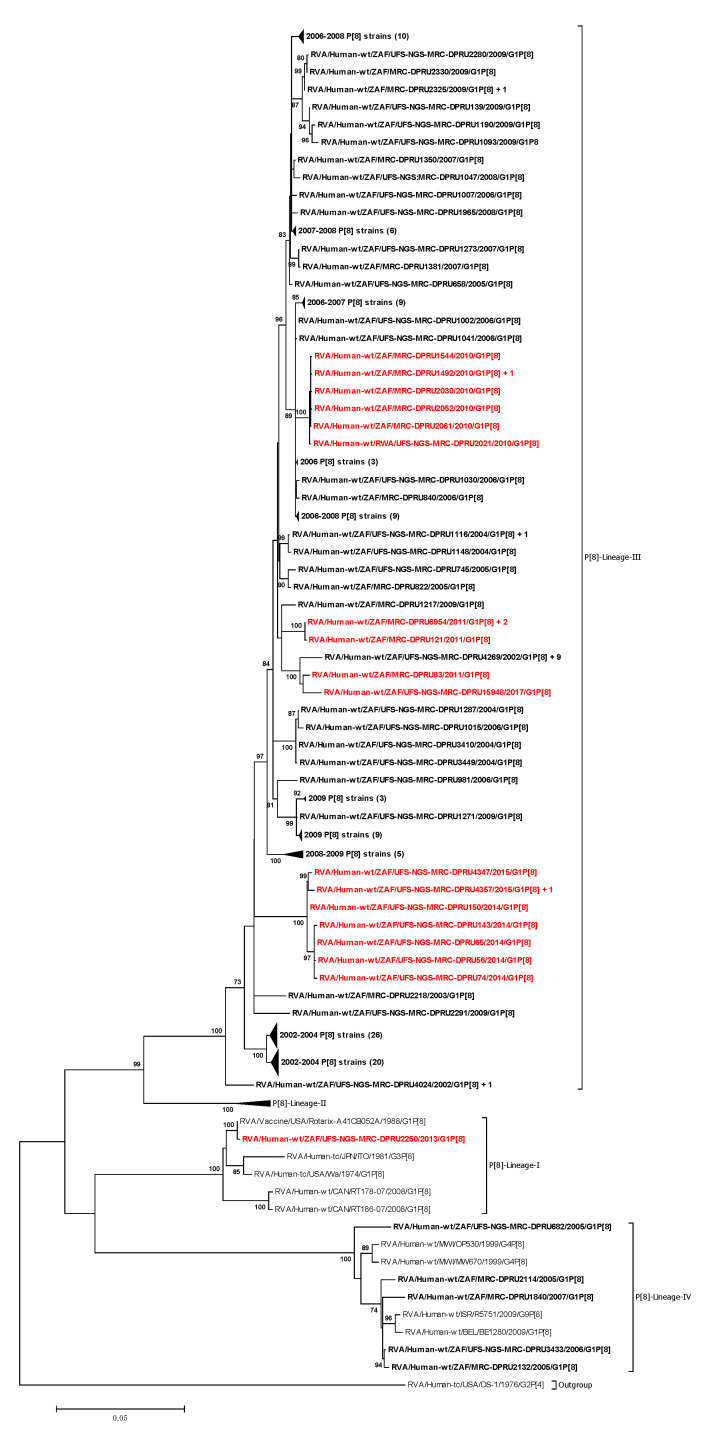
Maximum likelihood phylogenetic tree based on the full-length nucleotide sequences of genome segment 4 encoding VP4 protein. The GTR+G+I evolutionary model was used for phylogenetic inference. African pre-vaccine P[8] strains are highlighted in bold-face while post-vaccine strains are highlighted in bold-red. Adjacent to some sequences is indicated a plus (+) sign followed by the number of identical sequences (S3). The number in brackets denotes the number of compressed strains. Lineages are indicated in roman numerals. Only bootstrap values ≥ 70% are shown adjacent to each branch node. Scale bar indicates the number of nucleotide substitutions per site.

The VP4 phylogenetic tree comprised nucleotide sequences of the South African RVA strains and those of the reference strains from the four established VP4 P[8] genotype lineages (I to IV) [11] (Figure 2). The P[8] strains for South African rotaviruses segregated into three evolutionary lineages, P[8]-lineage I, III, and IV (Figure 2). Lineage III comprised a mixture of 144 pre-vaccine and 21 post-vaccine P[8] strains, while lineage IV comprised five pre-vaccine strains (Figure 2). The P[8] strains identified pre- and post-vaccination were highly identical to each other and similarly distant from the Rotarix^®^ strain (Table 1).

#### 3.2.2. Phylogenetic Analysis of VP1–VP3, VP6, and NSP1–NSP5

South African VP1–VP3, VP6, and NSP1–NSP5 were clustered into two main lineages, one of the lineages comprising of the Rotarix^®^ genome segments (Figures S1–S9 in S4). South African strains identified pre- and post-vaccination were highly identical to each other as well as similarly distant from the vaccine strain (Table 1).

### 3.3. Protein Modeling and Amino Acid Analysis

#### 3.3.1. Comparative Analysis of Neutralizing Antigenic VP7 Epitopes of South African G1 Strains and Rotarix^®^ Strains

**Figure 3:**
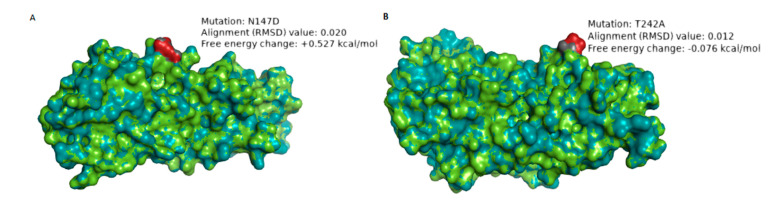
Structure models showing the mutation sites N147D (**A**) and T242A (**B**), respectively, that were only present in post-vaccine G1P[8] strains when superposed with VP7 of Rotarix^®^. The structure of the study strain is in green, while the Rotarix^®^ structure is in deep-teal. The amino acid residues are represented in dark gray and red to examine whether the replacing amino acid alters the conformation of the protein structure. The amino acid residue highlighted in dark gray represents the study strain while that in firebrick red represents the Rotarix^®^ strain. RMSD is the superposition value where the value of zero indicates absolute similarity. The stability of the protein after mutation was measured in kcal/mol, whereby free energy change of ±0.5 kcal/moL was regarded as significant for the stabilizing/destabilizing effect.

The VP7 protein contains two defined neutralization antigenic epitopes spanning across 29 amino acid residues: 7–1 (7–1a and 7–1b) and 7–2 regions [42]. A comparison of the amino acid residues in the antigenic epitopes of wild type South African G1 strains with cognate sites in the Rotarix^®^ strain was performed (Figure S1 in S5). Five amino acid differences appeared in both pre- and post-vaccine strains, four amino acids occurred before vaccine introduction while two amino acid differences, N147D (in eight strains) and T242A (in one strain), appeared only during the post-vaccine period (Figure S1 in S5; Table S1 in S6). We performed protein modeling analysis on the post-vaccine strains, which showed amino acid differences relative to both the pre-vaccine strains and Rotarix^®^ strain. Therefore, for VP7, we selected strain RVA/Human-wt/ZAF/UFS-NGS-MRC-DPRU4357/2015/G1P[8] as a representative strain of the eight G1 post-vaccine strains that had N147D amino acid substitution (Figure S1 in S5). Additionally, we selected strain RVA/Human-wt/ZAF/MRC-DPRU1544/2010/G1P[8], the only post-vaccine strain exhibiting a T242A amino acid difference (Figure S1 in S5). The VP7 protein structures of the selected G1 strains were superposed with the Rotarix^®^ VP7 structure to assess differences in the structural conformation. The VP7 structure of strain RVA/Human-wt/ZAF/UFS-NGS-MRC-DPRU4357/2015/G1P[8] when superposed with the Rotarix^®^ VP7 had a root mean square deviation (RMSD) value of 0.020 Å (Table 2; Figure 3A). The RMSD value closer to zero suggests high structural homology [43]. Replacement of asparagine with aspartate (N147D) significantly destabilized the protein structure as the free energy change was +0.527 kcal/mol [40] (Table 2; Figure 3A). Apart from the VP7 protein structure of Rotarix^®^, we performed protein modeling analysis using the protein structures of five randomly selected pre-vaccine study strains (Figure S1 in S7). Similar significant free energy change trends for the destabilizing effect were observed and ranged from +0.506 to +0.579 kcal/mol (Figure S1 in S7). The VP7 structure of strain RVA/Human-wt/ZAF/MRC-DPRU1544/2010/G1P[8] had an RMSD value of 0.012 Å when superposed with Rotarix^®^ VP7 (Table 2; Figure 3B). Threonine, which is a neutral hydrophilic amino acid, was replaced with alanine, an aliphatic hydrophobic amino acid, showing a change in polarity. However, the replacing amino acid (alanine) does not significantly alter the stability of the protein at this epitope (−0.076 kcal/mol) [40]. Protein modeling analysis with VP7 protein structures from five randomly selected pre-vaccine study strains showed similar free energy change trends ranging from −0.070 to −0.078 kcal/mol (Figure S2 in S7).

#### 3.3.2. Comparative Analysis of VP7 Cytotoxic T Lymphocyte Epitopes of South Africa With Rotarix^®^ Vaccine Strain

The two established VP7 cytotoxic T-cell lymphocyte epitopes at positions 16–28 and 40–52 [44] of the VP7 of South African G1P[8] strains were compared with cognate regions of Rotarix^®^ VP7. Five amino acid differences appeared before Rotarix^®^ introduction, while two amino acid substitutions (A43V in two strains and A46T in four strains) were found after vaccine introduction (Table S1 in S6). Comparative analysis of the South African post-vaccine G1 genotypes outside the known epitope regions with cognate regions in G1 of Rotarix^®^ identified three amino acid differences (S37I, A68D, and E222K) (S8). The replacing amino acid at positions S37I, A68D, and E222K resulted in a change in the polarity of the amino acid residue (Table S2 in S6).

#### 3.3.3. Comparative Analysis of Neutralizing Antigenic Epitopes in VP4 Genes of South African P[8] Strains and Rotarix^®^ Vaccine Strain

**Figure 4:**
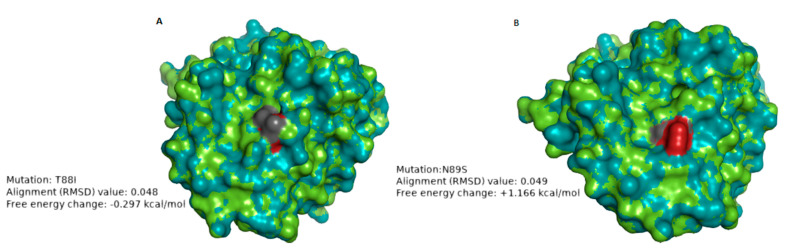
VP4 structure models showing mutations at sites T88I (**A**) and N89S (**B**), respectively, that were only seen in some post-vaccine strains when superposed with VP4 of Rotarix^®^. The structure of the study strain is in green while the Rotarix^®^ structure is in deep-teal. The amino acid residues are represented in dark gray and red to examine whether the replacing amino acid alters the conformation of the protein structure. The amino acid residue highlighted in dark gray represents the study strain while the firebrick red represents the Rotarix^®^ strain. The stability of the protein after mutation was measured in kcal/mol, whereby the folding energy change of ±0.5 kcal/mol is regarded as statistically significant for either stabilizing (−)/destabilizing (+) effect.

The VP4 protein comprises the VP5* and VP8* regions. The VP8* region contains four (8–1 to 8–4) neutralizing antigenic epitopes, while VP5* has five (5–1 to 5–5) [45]. The differences between the P[8] study strains and Rotarix^®^ P[8] component were mostly contained in the VP8* epitopes 8–1, 8–3, and 8–4 (Figure S2 in S5). Seven amino acid differences were identified in both pre- and post-vaccine strains, seven amino acid substitutions during pre-vaccination introduction and two amino acid substitutions, T88I (in one strain) and N89S (in one strain) were identified after vaccine introduction (Figure S2 in S5; Table S3 in S6). We performed protein modeling analysis on the post-vaccine strains that showed amino acid differences relative to both the pre-vaccine strains and Rotarix^®^ strain and these were RVA/Human-wt/ZAF/UFS-NGS-MRC-DPRU74/2014/G1P[8] and RVA/Human-wt/ZAF/UFS-NGS-MRC-DPRU83/2011/G1P[8] (Figure S2 in S5). The VP4 structure of strain RVA/Human-wt/ZAF/UFS-NGS-MRC-DPRU74/2014/G1P[8] aligned with the RMSD value of RMSD 0.048 when superposed with the Rotarix^®^ VP4 (Table 3; Figure 4A). Replacement with the isoleucine at position T88I altered the polarity of the residue from polar to nonpolar without affecting the charge [46]. This change in polarity may alter the physical properties of the protein. The mutation did not significantly impact the stability of the protein (−0.297 kcal/mol) (Table 3; Figure 4A). Further free energy change analysis with VP4 structures from five randomly selected pre-vaccine strains demonstrated similar free energy change trends that ranged from −0.504 to −0.322 kcal/mol (Figure S3 in S7).The VP4 structure of strain RVA/Human-wt/ZAF/MRC-DPRU83/2011/G1P[8] had an RMSD value of 0.049 when superposed with Rotarix^®^ VP4, indicating significant alignment. Asparagine is a polar, neutrally charged amino acid, and the replacing amino acid, serine, is also polar with a neutral charge [46]. The replacing amino acid, serine, significantly destabilized the protein structure with +1.166 kcal/mol energy change (Table 3: Figure 4B).When protein modeling analysis was performed with VP4 structures of five randomly selected pre-vaccine P[8] strains, significant free energy change values ranging from +1.128 to +1.352 kcal/mol were also observed, indicating the amino acid substitution had a destabilizing effect on the protein structure (Figure S4 in S7).

#### 3.3.4. Analysis of the VP4 and VP6 Non-Neutralizing Regions

Comparative analysis of the South African post-vaccine P[8] strain outside the known epitope regions with cognate regions in pre-vaccine strains identified five amino acid differences that were conservative in nature (Table S4 in S6). Analysis of the amino acid differences between the VP6 gene sequences of South African pre- and post-vaccine strains identified an amino acid difference Y353H in a 2017 post-vaccine strain, RVA/Human-wt/ZAF/MRC-DPRU15948/2017/G1P[8] (S8). The amino acid mutation Y353H occurred in a conserved region of the VP6 that has been associated with trimerization and single-shelled particle formation [47] (Table S5 in S6).

#### 3.3.5. Analysis of Amino Acid Differences in VP1–VP3 and NSP1–NSP5 Amino Acid Sequences

The number of amino acid differences that were identified after Rotarix^®^ vaccine introduction in South African G1P[8] VP1–VP3 and NSP1–NSP5 amino acid sequences were 12, 6, 7, 11, 3, 4, 5, and 2, respectively (Tables S6–S13 in S6). Most of the amino acid substitutions were conservative (46).

#### 3.3.6. Analyses of Selection Pressure and Recombination

Selection pressure analysis by Mixed-Effects Model of Evolution (MEME), Fast, Unconstrained Bayesian AppRoximation for inferring selection (FUBAR), and Fixed-Effects Likelihood (FEL) showed that most of the codon sites in the 11 gene segments were undergoing purifying selection. With the exception of the VP7 gene segment, several amino acid sites in the rest of the gene segments were identified to be under positive selective pressure (Table 4) Amino acid sites 7 and 245 were identified to be under positive selective pressure by all three analysis tools used (Table 4). GARD found no significant evidence of recombination in all gene segments.

## 4. Discussion

The present study reports whole-genome analysis of G1P[8] RVA strains collected seven years before and seven years after the introduction of the Rotarix^®^ vaccine in South Africa. Whole-genome analysis of South African G1P[8] strains showed that they possessed a Wa-like genotype constellation. These findings are comparable to what has been observed in several countries [11,48,49,50,51]. The prevalent association of G1P[8] genotypes with the Wa-like genetic background has been hypothesized to be due to epidemiological fitness [52].

Of the seven previously described G1 lineages [9], South African pre- and post-vaccine G1 strains in this study were segregated into G1-lineage I and G1-lineage II. Similar observations have been reported in several other studies [11,51,53], highlighting the likelihood of the epidemiological fitness of G1 strains in these two lineages, which has led to their prevalent circulation over time. Of the four P[8] lineages that have been established previously [11], the majority (84.2%) of the South African P[8] strains in this study clustered in P[8] lineage III, an observation comparable to other studies [51,53,54,55]. Additionally, the South African P[8] strains were observed in P[8] lineage I and P[8] lineage IV (also designated as the OP354-like lineage) [56], an observation reported in a study of Indian P[8] strains [57]. Both pre-and post-vaccine strains were found present in this P[8] lineage III, underscoring its global predominance in circulation [9,56,57,58,59,60].

Amino acid differences in the neutralization epitopes of VP7 compared to the cognate region in Rotarix^®^ identified at positions N94S, S123N, K291R, and M217T appeared in both pre- and post-vaccine strains and have been previously reported in Belgium and Brazil [51,61]. Since these amino acid substitutions were present even before the introduction of the vaccine, they can be attributed to the natural RVA evolutionary processes. Amino acid differences in N147D occurred in eight post-vaccine G1 strains resulted in a change from neutral to negatively charged amino acid residue, while T24A occurred in one post-vaccine G1 strain, which resulted in a change from hydrophilic to hydrophobic [46]. The change in charge may affect the protein’s chemical properties, while the shift in polarity suggests possible inaccessibility of the epitope as it becomes more hydrophobic [46]. Differences in VP4 amino acids between the study strains and Rotarix^®^ were observed in epitopes 8–1 and 8–3, similar to those already reported in Belgium, Brazil, and Tunisia [51,61,62]. The polarity changes (hydrophilic to hydrophobic) observed at position T88I only in a post-vaccine strain may affect antibody binding at this region of the 8–4 epitope as the resulting epitope becomes relatively inaccessible due to the hydrophobic effect [46]. The loss of glycosylation site at position N89S only observed in the post-vaccine period in one strain may alter the function of the protein at the 8–4 epitope [63].

To gain further insight into the impact of amino acid substitution occurring in the neutralization epitopes of VP7 and VP4 on the stability of the protein structures, we performed folding free energy change analysis. A protein’s folding free energy change is an essential aspect of the protein’s stability with a direct association with the protein’s function [64]. The N147D substitution in VP7 and N89S substitution in VP8* resulted in a predicted destabilizing effect on the protein structure. Due to this destabilization effect on the protein structure, as indicated by free energy change analysis, we hypothesize that these amino acid substitutions observed during the post-vaccine era may not be favored in the long-term to enhance viral fitness. We did not observe consistently occurring amino acid substitutions in the 11 gene segments in both the antigenic and non-antigenic regions throughout the post-vaccine period when contrasted to the pre-vaccine period. However, there were yearly observations such as L167M (in VP1), R44K (in VP2), I234V (in VP3), V112I (in VP4), T120I (in NSP1) and V73I (in NSP4), which occurred in all eight 2010 strains. Amino acid substitutions: K164R (in VP2), A411V (in VP3), A46T (in VP7), I341V (in NSP1), V254P (in NSP2), and D157E (in NSP5) of the four out of five 2011 strains and K96R (in VP1), V459I (in VP3), V600L (in VP4), and A259T (in NSP1) out of all the eight 2014 and 2015 G1P[8] strains.

Selection pressure analysis demonstrated that most of the codon sites in the 11 genome segments of South African G1P[8] strains were undergoing purifying selection, probably to purge deleterious polymorphisms that arise due to the inherent error-prone nature of the RNA polymerase enzyme [1]. Amino acid sites 7 and 245 in VP3 were identified to be under positive selection by all three selection pressure analysis methods and site 245 fell within the guanine-N7-methyltransferase (N7-MTpase) domain suggested to catalyze methylation during capping of nascent rotavirus transcripts [64]. Methylation capping by VP3 of RVA serves as a critical strategy to evade host antiviral innate immune response [65].

## 5. Conclusions

The study shows that there were no consistently conserved amino acid substitutions occurring throughout during post-RVA vaccine period in the antigenic and non-antigenic regions of South African G1P[8] strains as most of the amino acid substitutions found in the post-vaccine period were already present during the pre-vaccine period. Therefore, Rotarix^®^ did not appear to have an impact on the genetic changes of South African G1P[8] post-vaccine strains. However, continued long-term whole-genome surveillance to monitor any consistently occurring genetic changes during the post-vaccine period, which may hint at vaccine selective pressure, remains essential. This study was limited mainly by the significant differences in the sample size between the pre- and post-vaccine period, although the significant decline in G1P[8] strains in South Africa during post-vaccination period has been previously reported [21].

## Figures and Tables

**Table 1:** Nucleotide identity analysis between South African pre- and post-vaccine G1P[8] strains, pre-vaccine G1P[8] strains with Rotarix^®^, and post-vaccine G1P[8] strains with Rotarix^®^ strain.

Gene Segments/Nucleotide Identity Values in Percentage	VP7	VP4	VP6	VP1	VP2	VP3	NSP1	NSP2	NSP3	NSP4	NSP5
Comparison between pre- and post-vaccine G1P[8] strains	92.1–100	87.8–99.5	89.0–99.4	93.7–99.0	92.9–99.6	89.4–99.1	83.5–99.5	89.1–99.7	94.2–100	91.0–100	92.2–99.8
Comparison between pre-vaccine G1P[8] strains and Rotarix^®^ strain	92.7–100	89.6–91.1	88.2–98.7	94.5–99.1	93.1–99.0	91.2–98.6	83.6–100	88.3–90.9	95.3–100	91.4–98.9	92.2–98.8
Comparison between post-vaccine strains and Rotarix^®^ strain	93.3–100	89.9–99.9	88.9–100	94.5–100	93.0–100	90.9–99.9	84.0–99.9	89.7–100	97.1–100	91.0–100	92.9–100

South African pre- and post-vaccine G1P[8] strains were compared with each other and also compared with the Rotarix^®^ strain. The nucleotide identity values were calculated using the *p*-distance algorithm in MEGA 6 software (S2).

**Table 2:** Possible effects of amino acid mutations in South African post-vaccine G1 neutralization epitopes.

Strain Used for the Protein Modeling	Mutation	No. of Post-Vaccine Strain(s) with the Mutation	Region	Amino Acid Property Change	Superposition Value (RMSD)	Free Energy Change (kcal/mol)	Possible Effect
RVA/Human-wt/ZAF/UFS-NGS-MRC-DPRU4357/2015/G1P[8]	N147D	8	7–2 epitope	Hydrophilic to hydrophilic; Neutral to negative charge	0.020 Å	+0.527	The change in charge may alter the biochemical properties of the epitope. The mutation significantly destabilizes the structure of the protein.
RVA/Human-wt/ZAF/MRC-DPRU1544/2010/G1P[8]	T242A	1	7–1b epitope	Hydrophilic to hydrophobic; Neutral to Neutral charge	0.012 Å	−0.076	The change in polarity may alter the physicochemical property of the epitope

Relative mean square deviation (RMSD) is the superposition value where value of zero indicates absolute similarity. The stability of the protein after mutation was measured in kcal/mol, whereby free energy change of ±0.5 kcal/moL was regarded as significant for either stabilizing/destabilizing effect. Minus (−) value is indicative of stabilizing effect while positive (+) value is indicative of destabilizing effect.

**Table 3:** Possible effects of amino acid mutations in South African post-vaccine VP4 epitopes.

Strain Used for Protein Modeling	Mutation	No. of Strain(s) with the Mutation	Region	Amino ACID Property Change	Superimposition Value (RMSD)	Free Energy Change (kcal/mol)	Possible Effect
RVA/Human-wt/ZAF/UFS-NGS-MRC-DPRU74/2014/G1P[8]	T88I	1	8–4 epitope	Hydrophilic to hydrophobic; Neutral charge to neutral charge	0.048 Å	−0.297	The change in polarity may alter the physicochemical properties of the protein. No significant impact on the stability of the protein structure
RVA/Human-wt/ZAF/UFS-NGS-MRC-DPRU83/2011/G1P[8]	N89S	1	8–4 epitope	Hydrophilic to hydrophilic; Neutral charge to Neutral charge	0.049 Å	+1.166	The loss of the glycosylation site may alter the chemical properties of the protein. The mutation destabilized the protein structure.

Relative mean square deviation (RMSD) is the superimposition value where the value of zero indicates absolute similarity. The stability of the protein after mutation was measured in kcal/moL, whereby the folding energy change of ±0.5 kcal/mol is considered statistically significant for either stabilizing (−)/destabilizing (+) effect.

**Table 4:** Positively selected sites as identified by FEL, FUBAR, and MEME analysis.

Method	Amino Acid Sites in the Gene Segments under Positive Selection										
	VP1	VP2	VP3	VP4	VP6	VP7	NSP1	NSP2	NSP3	NSP4	NSP5
**MEME**	2, 3, 5, 89, 243, 357, 733, 909, 1082	583	**7**, 118, 137, **245**, 310, 728	23, 28, 44, 90, 169, 201, 576, 578, 668, 773, 774, 775	85, 238	-	14, 32, 90, 132, 154, 175, 181, 225, 253, 261, 267, 293, 392, 396, 398, 404, 405, 416, 425	55, 258, 315	204	25, 168	3
**FUBAR**	67, 357	12, 36	**7**, **245**	-	-	-	-	75	308	-	-
**FEL**	3	-	**7**, **245**	-	-	-	-	-	-	-	-

Amino acid sites that were identified to be under positive selection by FEL, MEME, and FUBAR. Amino acid sites in bold and underlined were identified by all the three methods. For MEME and FEL, statistical significance was assessed at *p* ≤ 0.1 while for FUBAR, it was assessed at posterior probability ≥0.9. The dash (–) sign indicates no site was identified. Additional analyses reports are provided in S9.

## Supplementary Materials

The following are available online at https://www.mdpi.com/2076-393X/8/4/609/s1, Supplementary data 1(S1): Whole genome constellation analysis containing Table S1: Whole genotype constellations of 103 sequenced South African G1P[8] rotavirus strains and 68 South African G1P[8] strains acquired from GenBank. Supplementary data 2 (S2): Identity matrices analysis for VP1, VP2, VP3, VP4, VP6, NSP1, NSP2, NSP3, NSP4, and NSP5 nucleotide and deduced amino acid identities among strains calculated by distance matrices using p-distance algorithm in MEGA 6. Supplementary data 3 (S3): Duplicate sequences in the alignments identified using ElimDupes. Supplementary data 4 (S4): Additional phylograms containing Figure S1: Maximum likelihood phylogenetic tree based on the full length sequences of the genome segment 1 encoding VP1 protein, Figure S2: Maximum likelihood phylogenetic tree based on the full length sequences of the genome segment 2 encoding VP2 protein, Figure S3: Maximum likelihood phylogenetic tree based on the full length sequences of the genome segment 3 encoding VP3 protein, Figure S4: Maximum likelihood phylogenetic tree based on the full length sequences of the genome segment 6 encoding VP6 protein, Figure S5: Maximum likelihood phylogenetic tree based on the full length sequences of the genome segment 6 encoding NSP1 protein, Figure S6: Maximum likelihood phylogenetic tree based on the full length sequences of the genome segment 8 encoding NSP2 protein, Figure S7: Maximum likelihood phylogenetic tree based on the full length sequences of the genome segment 7 encoding NSP3 protein, Figure S8: Maximum likelihood phylogenetic tree based on the full length sequences of the genome segment 10 encoding NSP4 protein, Figure S9: Maximum likelihood phylogenetic tree based on the full length sequences of the genome segment 11 encoding NSP5 protein. Supplementary data 5 (S5): Neutralization epitope analysis in VP4 and VP7 containing Figure S1: Alignment of VP7 neutralizing and cytotoxic T-cell lymphocytic epitopes residues of Rotarix^®^ strain against wild type South African G1P[8] strains, Figure S2: Alignment of antigenic residues in VP4 between the P[8] component of Rotarix^®^ and wild type P[8] strains. Supplementary data 6 (S6): Additional tables containing Table S1: Amino acid differences in the VP7 neutralization and cytotoxic T-cell lymphocyte (CTL) epitope regions between Rotarix^®^ and South African G1 strains, Table S2: Amino acid differences in South African post-vaccine VP7 Cytotoxic T-cell Lymphocyte epitope and non-epitope regions, Table S3: Amino acid differences in the VP4 neutralization epitope regions between Rotarix^®^ and South African P[4] strains, Table S4: Amino acid differences in South African post-vaccine P[8] RVA strains, Table S5: Amino acid differences in South African post-vaccine I1 RVA strains, Table S6: Amino acid differences in South African post-vaccine R1 RVA strains, Table S7: Amino acid differences in South African post-vaccine C1 RVA strains, Table S8: Amino acid differences in South African post-vaccine M1 RVA strains, Table S9: Amino acid differences in South African post-vaccine A1 RVA strains, Table S10: Amino acid differences in South African post-vaccine N1 RVA strains, Table S11: Amino acid differences in South African post-vaccine T1 RVA strains, Table S12: Amino acid differences in South African post-vaccine E1 RVA strains, Table S13: Amino acid differences in South African post-vaccine H1 RVA strains. Supplementary data 7(S7): Additional protein modeling analysis containing Figure S1: The VP7 structure models showing the N147D amino acid difference, Figure S2: The VP7 structure models showing the T242A amino acid difference, Figure S3: The VP4 structure models showing the T88I amino acid difference, Figure S4: The VP4 structure models showing the N89S amino acid difference. Supplementary data 8 (S8): Whole genome amino acid analysis, Supplementary data 9: Selection pressure analysis.
