# Supplementary material for: Whole Genome In-Silico Analysis of South African G1P[8] Rotavirus Strains before and after Vaccine Introduction over a Period of 14 Years
Source: Vaccines (Basel). 2020 Oct 14;8(4):609. doi: 10.3390/vaccines8040609 (PMC7712154; doi:10.3390/vaccines8040609)
Supplement: Supplementary file 1 [file vaccines-08-00609-s001.zip › supplementary--R1--AE/Supplementary data 4_Additional phylograms.pdf]

Supplementary data 4: Additional phylograms

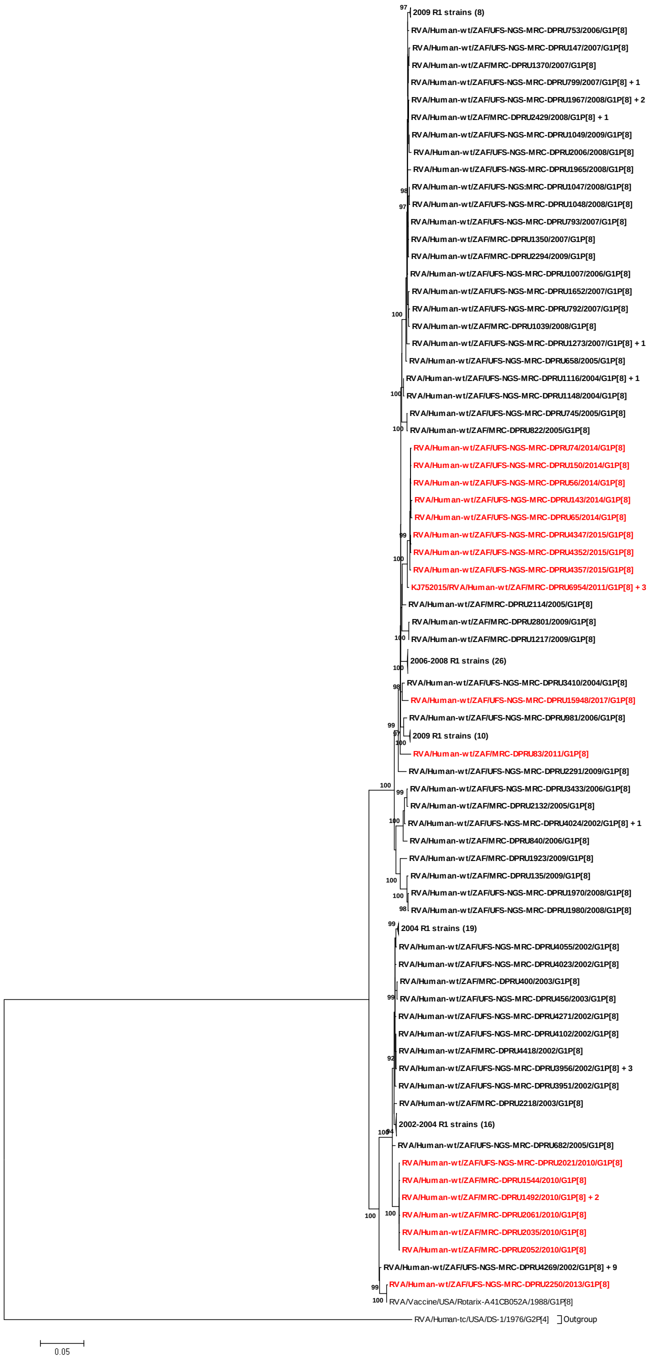

Figure S1: Maximum likelihood phylogenetic tree based on the full-length nucleotide sequences of the genome segment 1 encoding VP1 protein. The T92+G+I evolutionary model was used for phylogenetic inference. South African pre-vaccine R1 strains are highlighted in bold-face while post-vaccine strains are highlighted in bold-red. Adjacent to some sequences is indicated a plus (+) sign followed by the number of identical sequences (S3). The number in brackets denotes the number of compressed strains. Only bootstrap values ≥ 70% are shown adjacent to each branch node. Scale bar indicates the number of nucleotide substitutions per site.

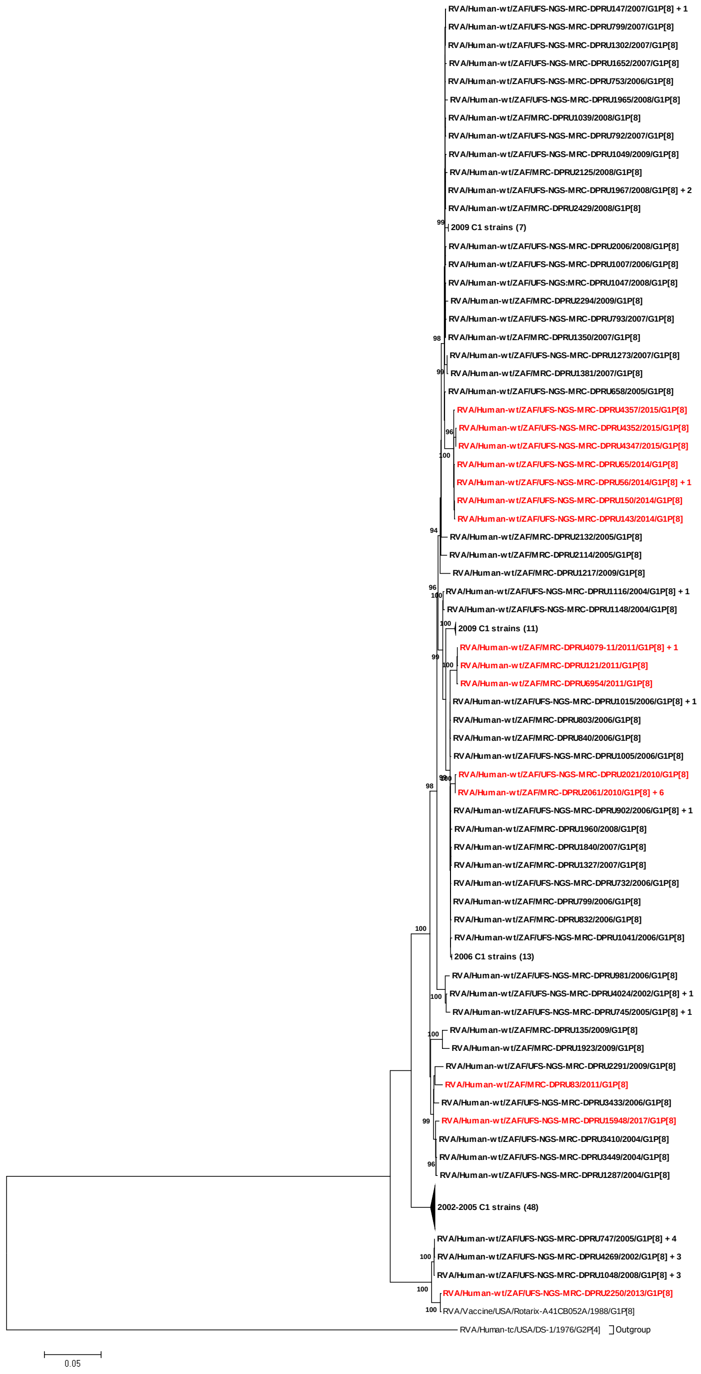

Figure S2: Maximum likelihood phylogenetic tree based on the full-length nucleotide sequences of the genome segment 2 encoding VP2 protein. The T92+G+I evolutionary model was used for phylogenetic inference. South African pre-vaccine C1 strains are highlighted in bold-face while post-vaccine strains are highlighted in bold-red. Adjacent to some sequences is indicated a plus (+) sign followed by the number of identical sequences (S3). The number in brackets denotes the number of compressed strains. Only bootstrap values ≥ 70% are shown adjacent to each branch node. Scale bar indicates the number of nucleotide substitutions per site.

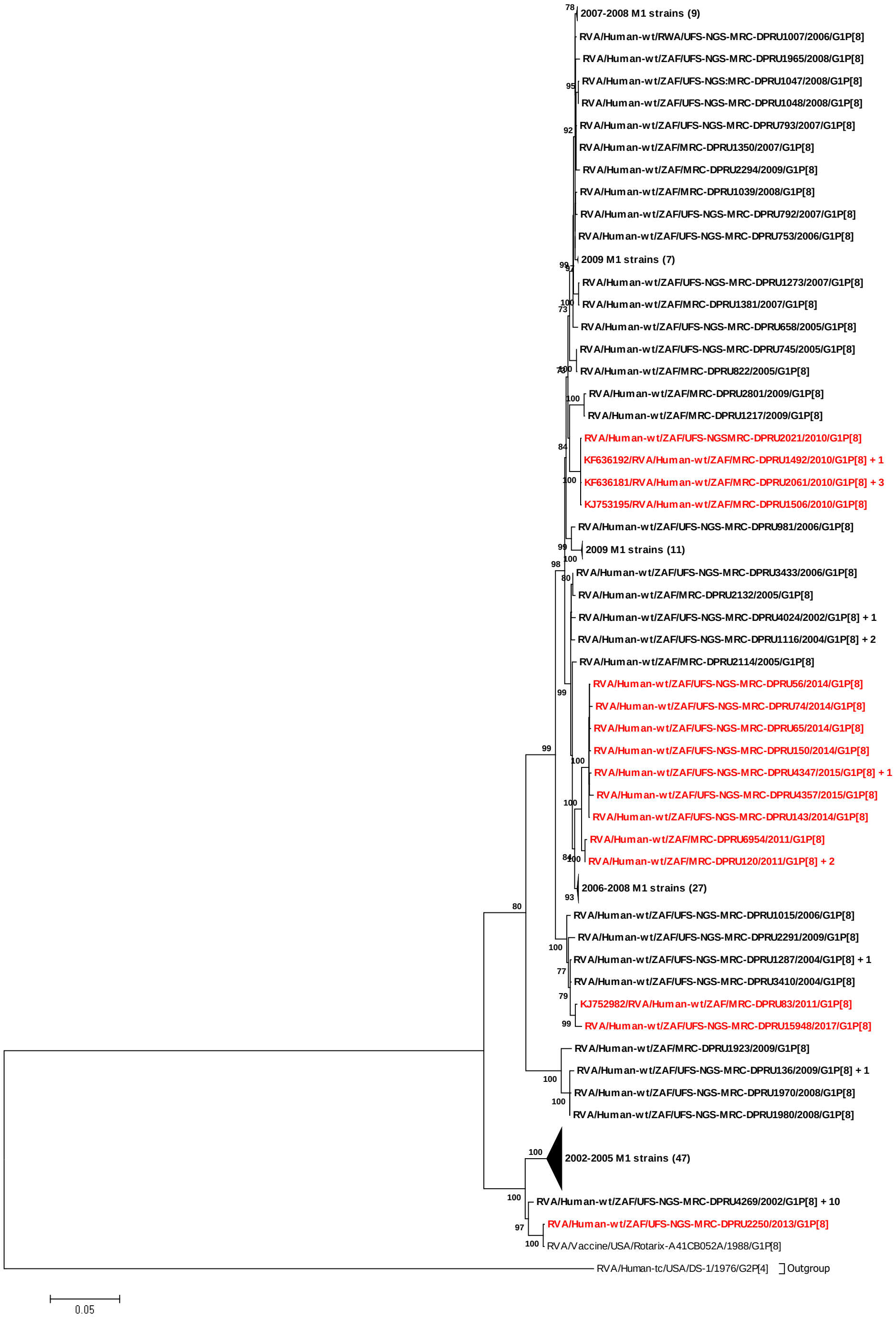

Figure S3: Maximum likelihood phylogenetic tree based on the full-length nucleotide sequences of the genome segment 3 encoding VP3 protein. The GTR+ G evolutionary model was used for phylogenetic inference. South African pre-vaccine M1 strains are highlighted in bold-face while post-vaccine strains are highlighted in bold-red. Adjacent to some sequences is indicated a plus (+) sign followed by the number of identical sequences (S3). The number in brackets denotes the number of compressed strains. Only bootstrap values ≥ 70% are shown adjacent to each branch node. Scale bar indicates the number of nucleotide substitutions per site.

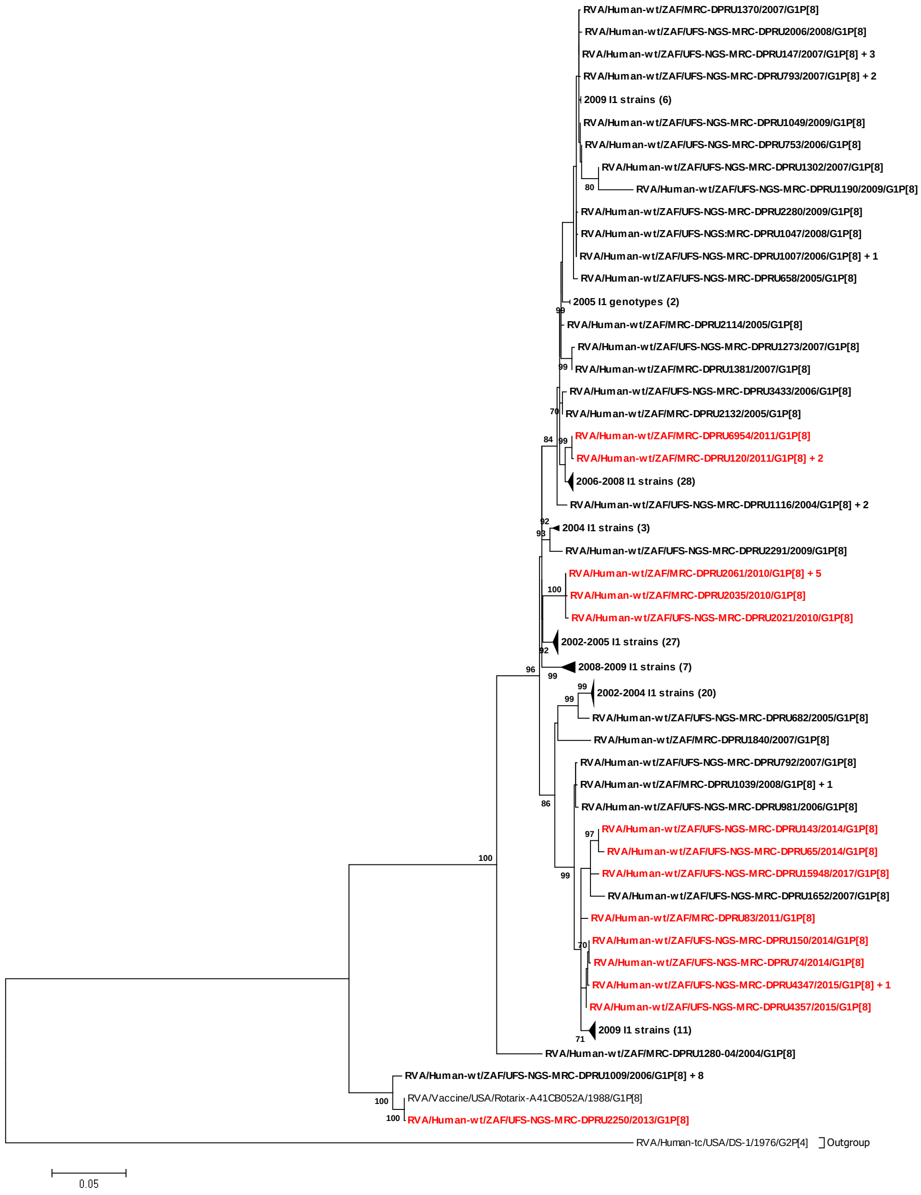

Figure S4: Maximum likelihood phylogenetic tree based on the full-length nucleotide sequences of the genome segment 6 encoding VP6 protein. The T92+G+I evolutionary model was used for phylogenetic inference. South African pre-vaccine I1 strains are highlighted in bold-face while post-vaccine strains are highlighted in bold-red. Adjacent to some sequences is indicated a plus (+) sign followed by the number of identical sequences (S3). The number in brackets denotes the number of compressed strains. Only bootstrap values ≥ 70% are shown adjacent to each branch node. Scale bar indicates the number of nucleotide substitutions per site.

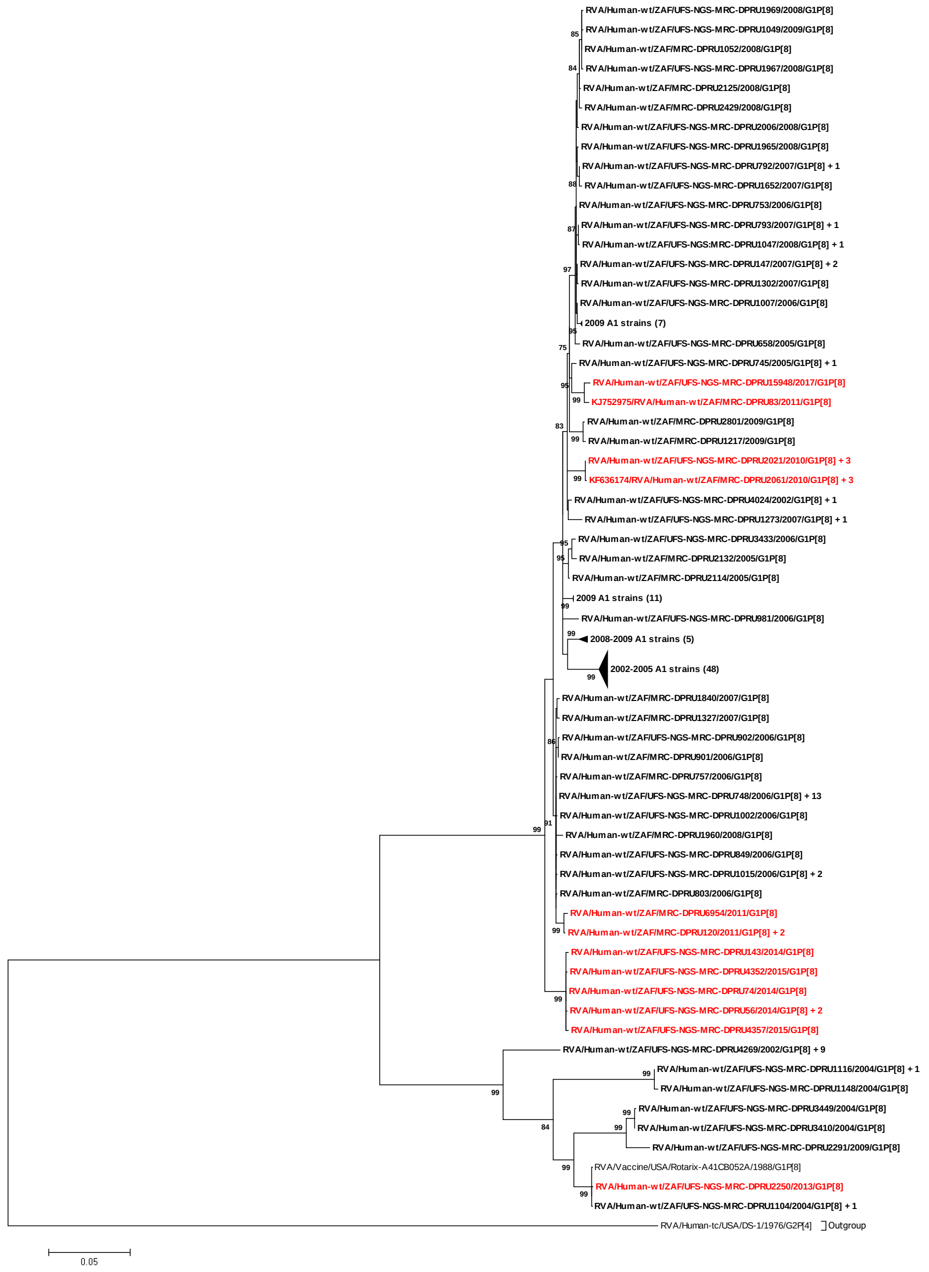

Figure S5: Maximum likelihood phylogenetic tree based on the full-length nucleotide sequences of the genome segment 5 encoding NSP1 protein. The T92 + I evolutionary model was used for phylogenetic inference. South African pre-vaccine A1 strains are highlighted in bold-face while post-vaccine strains are highlighted in bold-red. Adjacent to some sequences is indicated a plus (+) sign followed by the number of identical sequences (S3). The number in brackets denotes the number of compressed strains. Only bootstrap values ≥ 70% are shown adjacent to each branch node. Scale bar indicates the number of nucleotide substitutions per site.

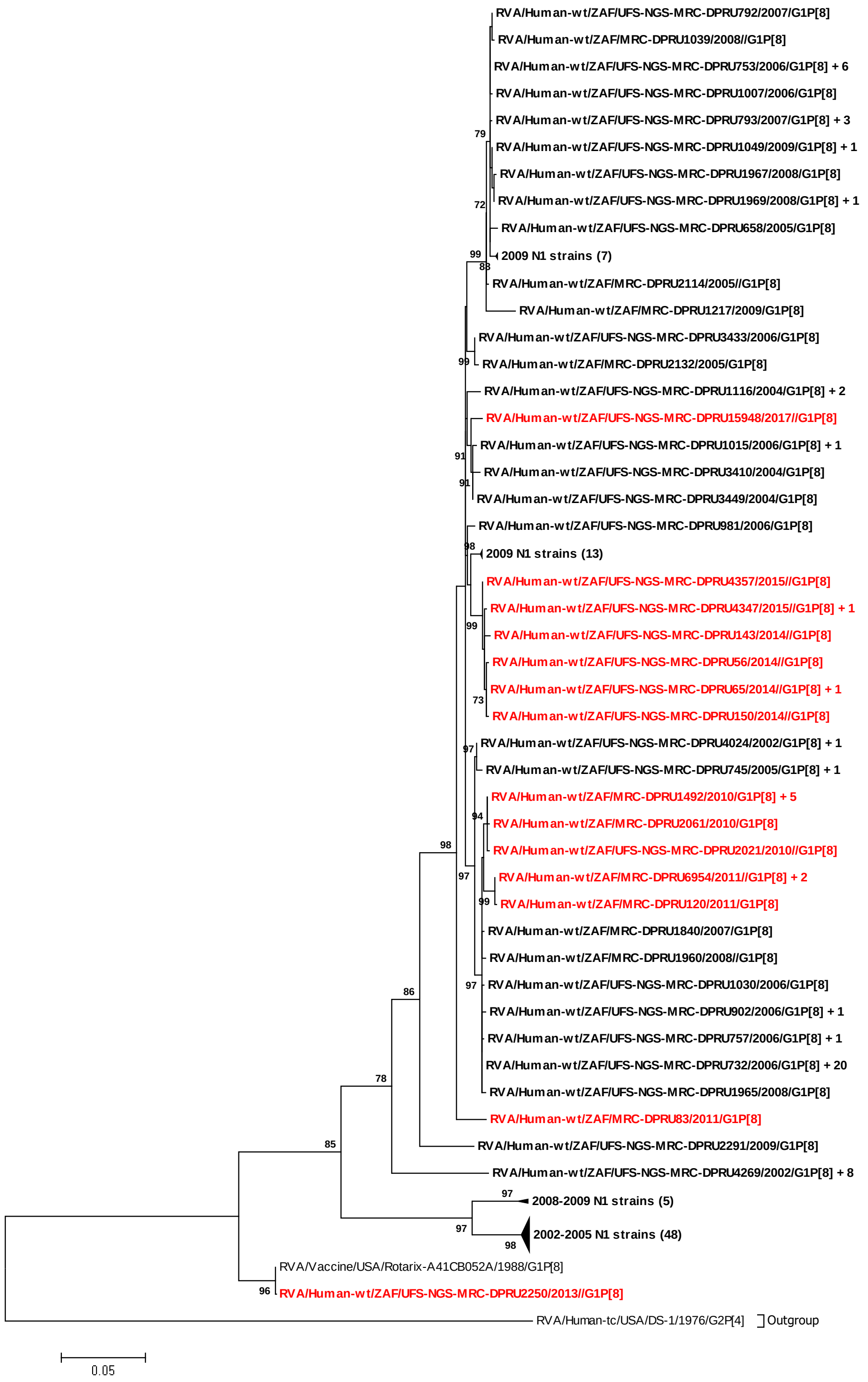

Figure S6: Maximum likelihood phylogenetic tree based on the full-length nucleotide sequences of the genome segment 8 encoding NSP2 protein. The T92 + G evolutionary model was used for phylogenetic inference. South African pre-vaccine N1 strains are highlighted in bold-face while post-vaccine strains are highlighted in bold-red. Adjacent to some sequences is indicated a plus (+) sign followed by the number of identical sequences (S3). The number in brackets denotes the number of compressed strains. Only bootstrap values ≥ 70% are shown adjacent to each branch node. Scale bar indicates the number of nucleotide substitutions per site.

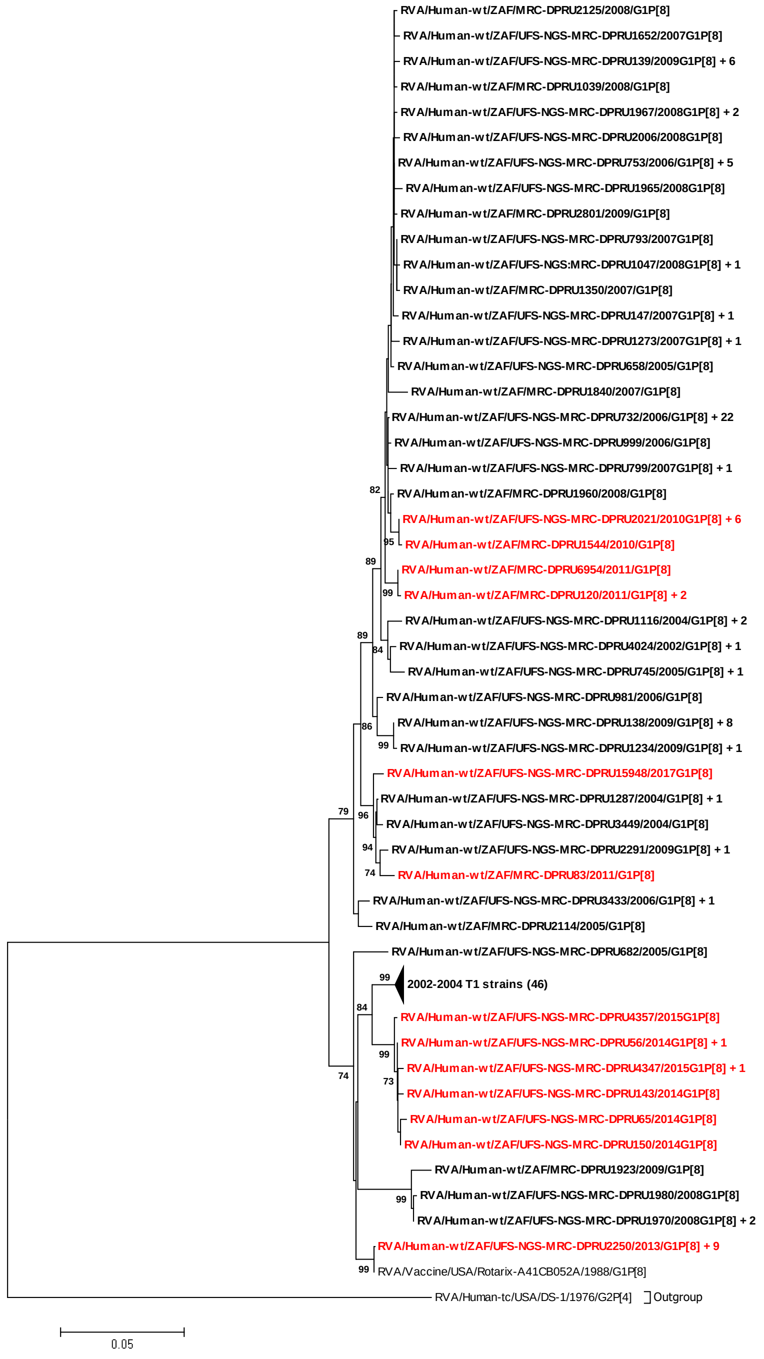

Figure S7: Maximum likelihood phylogenetic tree based on the full-length nucleotide sequences of the genome segment 7 encoding NSP3 protein. The TN93 + I evolutionary model was used for phylogenetic inference. South African pre-vaccine T1 strains are highlighted in bold-face while post-vaccine strains are highlighted in bold-red. Adjacent to some sequences is indicated a plus (+) sign followed by the number of identical sequences (S3). The number in brackets denotes the number of compressed strains. Only bootstrap values ≥ 70% are shown adjacent to each branch node. Scale bar indicates the number of nucleotide substitutions per site.

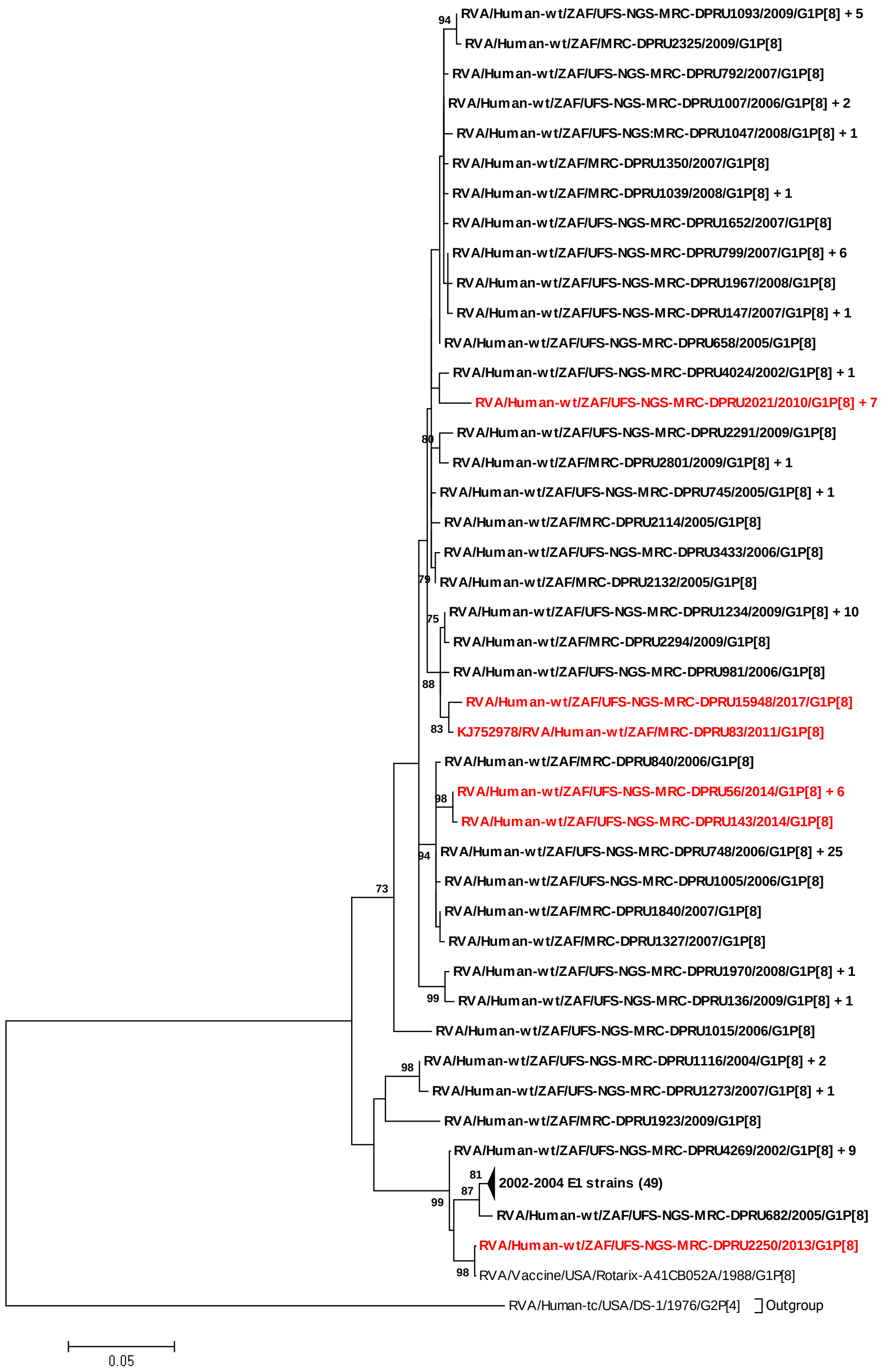

Figure S8: Maximum likelihood phylogenetic tree based on the full-length nucleotide sequences of the genome segment 10 encoding NSP4 protein. The T92 +G evolutionary model was used for phylogenetic inference. South African pre-vaccine E1 strains are highlighted in bold-face while post-vaccine strains are highlighted in bold-red. Adjacent to some sequences is indicated a plus (+) sign followed by the number of identical sequences (S3). The number in brackets denotes the number of compressed strains. Only bootstrap values ≥ 70% are shown adjacent to each branch node. Scale bar indicates the number of nucleotide substitutions per site.

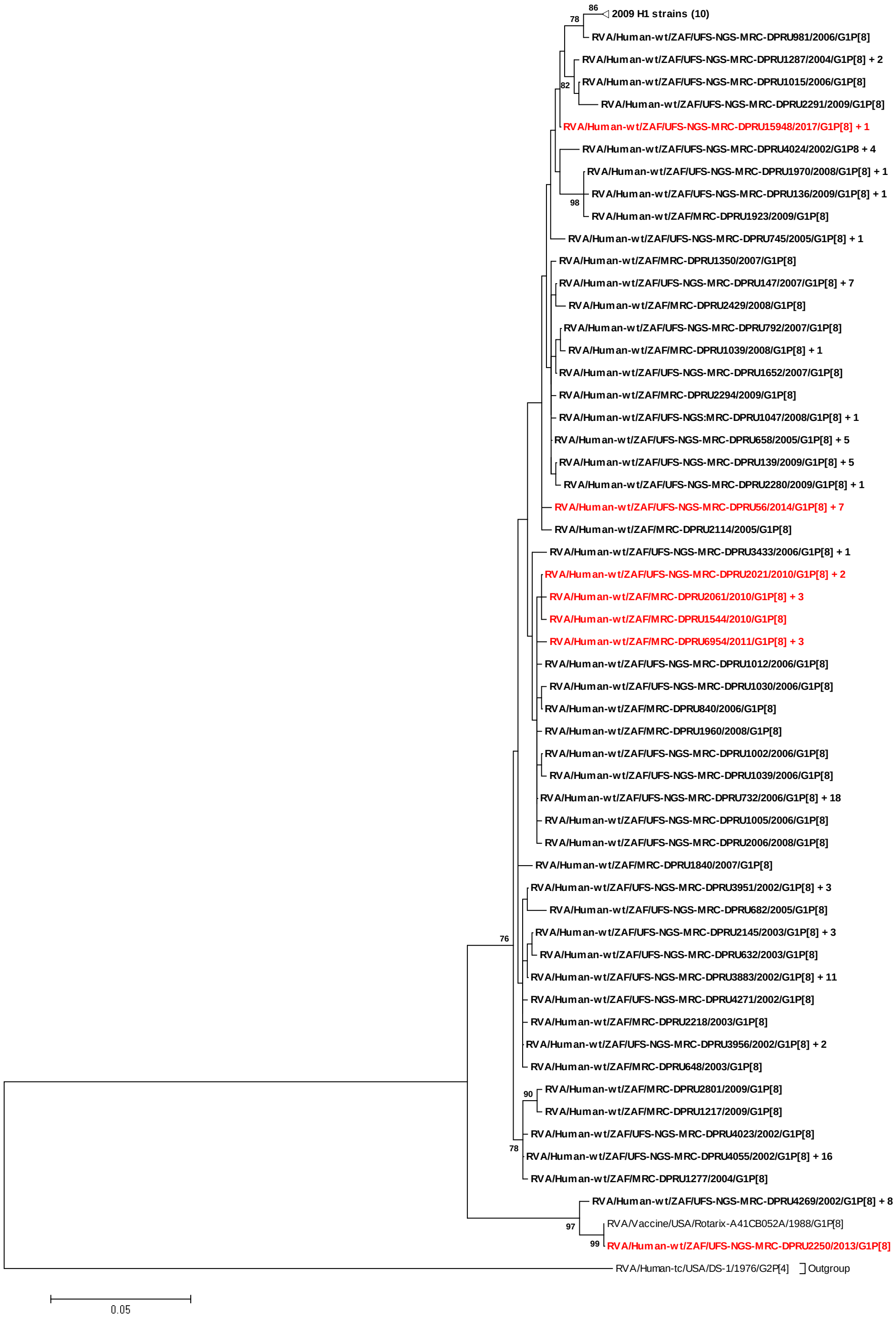

Figure S9: Maximum likelihood phylogenetic tree based on the full-length nucleotide sequences of the genome segment 11 encoding NSP5 protein. The T92 + I evolutionary model was used for phylogenetic inference. South African pre-vaccine H1 strains are highlighted in bold-face while post-vaccine strains are highlighted in bold-red. Adjacent to some sequences is indicated a plus (+) sign followed by the number of identical sequences (S3). The number in brackets denotes the number of compressed strains. Only bootstrap values ≥ 70% are shown adjacent to each branch node. Scale bar indicates the number of nucleotide substitutions per site.
