# Supplementary material for: Whole Genome In-Silico Analysis of South African G1P[8] Rotavirus Strains before and after Vaccine Introduction over a Period of 14 Years
Source: Vaccines (Basel). 2020 Oct 14;8(4):609. doi: 10.3390/vaccines8040609 (PMC7712154; doi:10.3390/vaccines8040609)
Supplement: Supplementary file 1 [file vaccines-08-00609-s001.zip › supplementary--R1--AE/Supplementary data 5_ Neutralization epitopes analysis in VP4 and VP7.pdf]

Supplementary data 5: Neutralization epitopes in VP7 and VP4

		Neutralization epitopes																												Cytotoxic T. lymphotcytes (CTL) epitopes																									
		7-1a														7-1b						7-2									T-cell epitope 16-28 aa													T-cell epitope 40-52 aa												
		•	•	•	•	•	•	•	•	•						•	•		•	•			•		•	•	•	•		•																									
		87	91	94	96	97	98	99	100	104	123	125	129	130	291	201	211	212	213	238	242	143	145	146	147	148	190	217	221	264	16	17	18	19	20	21	22	23	24	25	26	27	28	40	41	42	43	44	45	46	47	48	49	50	51	52
	Vaccine strain																																																							
	RVA/Vaccine/USA/Rotarix-A41CB052A/1988/G1P[8]	T	T	N	G	E	W	K	D	Q	S	V	V	D	K	Q	N	V	D	N	T	K	D	Q	N	L	S	M	N	G	I	I	L	L	N	Y	I	L	K	S	V	T	R	I	Y	V	A	L	F	A	L	T	R	A	Q	N
	South African pre-vaccine strains																																																						
1	RVA/Human-wt/ZAF/UFS-NGS-MRC-DPRU3883/2002/G1P[8]	T	T	N	G	E	W	K	D	Q	S	V	V	D	K	Q	N	V	D	N	T	K	D	Q	N	L	S	M	N	G	I	I	L	L	N	Y	I	L	K	S	V	T	R	I	S	V	A	L	F	A	L	T	R	A	Q	N
2	RVA/Human-wt/ZAF/UFS-NGS-MRC-DPRU3892/2002/G1P[8]	T	T	N	G	E	W	K	D	Q	S	V	V	D	K	Q	N	V	D	N	T	K	D	Q	N	L	S	M	N	G	I	I	L	L	N	Y	I	L	K	S	V	T	R	I	S	V	A	L	F	A	L	T	R	A	Q	N
3	RVA/Human-wt/ZAF/UFS-NGS-MRC-DPRU3951/2002/G1P[8]	T	T	N	G	E	W	K	D	Q	S	V	V	D	K	Q	N	V	D	N	T	K	D	Q	N	L	S	M	N	G	I	I	L	L	N	Y	I	L	K	S	V	T	R	I	S	V	A	L	F	A	L	T	R	A	Q	N
4	RVA/Human-wt/ZAF/UFS-NGS-MRC-DPRU3956/2002/G1P[8]	T	T	N	G	E	W	K	D	Q	S	V	V	D	K	Q	N	V	D	N	T	K	D	Q	N	L	S	M	N	G	I	I	L	L	N	Y	I	L	K	S	V	T	R	I	S	V	A	L	F	A	L	T	R	A	Q	N
5	RVA/Human-wt/ZAF/UFS-NGS-MRC-DPRU4018/2002/G1P[8]	T	T	N	G	E	W	K	D	Q	S	V	V	D	K	Q	N	V	D	N	T	K	D	Q	N	L	S	M	N	G	I	I	L	L	N	Y	I	L	K	S	V	T	R	I	S	V	A	L	F	A	L	T	R	A	Q	N
6	RVA/Human-wt/ZAF/UFS-NGS-MRC-DPRU4019/2002/G1P[8]	T	T	N	G	E	W	K	D	Q	S	V	V	D	K	Q	N	V	D	N	T	K	D	Q	N	L	S	M	N	G	I	I	L	L	N	Y	I	L	K	S	V	T	R	I	S	V	A	L	F	A	L	T	R	A	Q	N
7	RVA/Human-wt/ZAF/UFS-NGS-MRC-DPRU4023/2002/G1P[8]	T	T	N	G	E	W	K	D	Q	S	V	V	D	K	Q	N	V	D	N	T	K	D	Q	N	L	S	M	N	G	I	I	L	L	N	Y	I	L	K	S	V	T	R	I	S	V	A	L	F	A	L	T	R	A	Q	N
8	RVA/Human-wt/ZAF/UFS-NGS-MRC-DPRU4024/2002/G1P[8]	T	T	S	G	E	W	K	D	Q	N	V	V	D	R	Q	N	V	D	N	T	K	D	Q	N	L	S	T	N	G	I	I	L	L	N	Y	I	L	K	S	V	T	R	I	F	V	A	L	F	A	L	T	K	A	Q	N
9	RVA/Human-wt/ZAF/UFS-NGS-MRC-DPRU4026/2002/G1P[8]	T	T	S	G	E	W	K	D	Q	N	V	V	D	R	Q	N	V	D	N	T	K	D	Q	N	L	S	T	N	G	I	I	L	L	N	Y	I	L	K	S	V	T	R	I	F	V	A	L	F	A	L	T	K	A	Q	N
10	RVA/Human-wt/ZAF/UFS-NGS-MRC-DPRU4049/2002/G1P[8]	T	T	N	G	E	W	K	D	Q	S	V	V	D	K	Q	N	V	D	N	T	K	D	Q	N	L	S	M	N	G	I	I	L	L	N	Y	I	L	K	S	V	T	R	I	S	V	A	L	F	A	L	T	R	A	Q	N
11	RVA/Human-wt/ZAF/UFS-NGS-MRC-DPRU4055/2002/G1P[8]	T	T	N	G	E	W	K	D	Q	S	V	V	D	K	Q	N	V	D	N	T	K	D	Q	N	L	S	M	N	G	I	I	L	L	N	Y	I	L	K	S	V	T	R	I	S	V	A	L	F	A	L	T	R	A	Q	N
12	RVA/Human-wt/ZAF/UFS-NGS-MRC-DPRU4102/2002/G1P[8]	T	T	N	G	E	W	K	D	Q	S	V	V	D	K	Q	N	V	D	N	T	K	D	Q	N	L	S	M	N	G	I	I	L	L	N	Y	I	L	K	S	V	T	R	I	S	V	A	L	F	A	L	T	R	A	Q	N
13	RVA/Human-wt/ZAF/UFS-NGS-MRC-DPRU4269/2002/G1P[8]	T	T	N	G	E	W	K	D	Q	S	V	V	D	K	Q	N	V	D	N	T	K	D	Q	N	L	S	M	N	G	I	I	L	L	N	Y	I	L	K	S	V	T	R	I	Y	V	A	L	F	A	L	T	R	A	Q	N
14	RVA/Human-wt/ZAF/UFS-NGS-MRC-DPRU4271/2002/G1P[8]	T	T	N	G	E	W	K	D	Q	S	V	V	D	K	Q	N	V	D	N	T	K	D	Q	N	L	S	M	N	G	I	I	L	L	N	Y	I	L	K	S	V	T	R	I	S	V	A	L	F	A	L	T	R	A	Q	N
15	RVA/Human-wt/ZAF/UFS-NGS-MRC-DPRU456/2003/G1P[8]	T	T	N	G	E	W	K	D	Q	S	V	V	D	K	Q	N	V	D	N	T	K	D	Q	N	L	S	M	N	G	I	I	L	L	N	Y	I	L	K	S	V	T	R	I	S	V	A	L	F	V	L	T	R	A	Q	N
16	RVA/Human-wt/ZAF/UFS-NGS-MRC-DPRU493/2003/G1P[8]	T	T	N	G	E	W	K	D	Q	S	V	V	D	K	Q	N	V	D	N	T	K	D	Q	N	L	S	M	N	G	I	I	L	L	N	Y	I	L	K	S	V	T	R	I	S	V	A	L	F	A	L	T	R	A	Q	N
17	RVA/Human-wt/ZAF/UFS-NGS-MRC-DPRU609/2003/G1P[8]	T	T	N	G	E	W	K	D	Q	S	V	V	D	K	Q	N	V	D	N	T	K	D	Q	N	L	S	M	N	G	I	I	L	L	N	Y	I	L	K	S	V	T	Q	I	S	V	A	L	F	A	L	T	R	A	Q	N
18	RVA/Human-wt/ZAF/UFS-NGS-MRC-DPRU632/2003/G1P[8]	T	T	N	G	E	W	K	D	Q	S	V	V	D	K	Q	N	V	D	N	T	K	D	Q	N	L	S	M	N	G	I	I	L	L	N	Y	I	L	K	S	V	T	R	I	S	V	A	L	F	A	L	T	R	A	Q	N
19	RVA/Human-wt/ZAF/UFS-NGS-MRC-DPRU2117/2003/G1P[8]	T	T	N	G	E	W	K	D	Q	S	V	V	D	K	Q	N	V	D	N	T	K	D	Q	N	L	S	M	N	G	I	I	L	L	N	Y	I	L	K	S	V	T	R	I	Y	V	A	L	F	A	L	T	R	A	Q	N
20	RVA/Human-wt/ZAF/UFS-NGS-MRC-DPRU2145/2003/G1P[8]	T	T	N	G	E	W	K	D	Q	S	V	V	D	K	Q	N	V	D	N	T	K	D	Q	N	L	S	M	N	G	I	I	L	L	N	Y	I	L	K	S	V	T	R	I	S	V	A	L	F	A	L	T	R	A	Q	N
21	RVA/Human-wt/ZAF/UFS-NGS-MRC-DPRU2156/2003/G1P[8]	T	T	N	G	E	W	K	D	Q	S	V	V	D	K	Q	N	V	D	N	T	K	D	Q	N	L	S	M	N	G	I	I	L	L	N	Y	I	L	K	S	V	T	R	I	S	V	A	L	F	A	L	T	R	A	Q	N
22	RVA/Human-wt/ZAF/UFS-NGS-MRC-DPRU1104/2004/G1P[8]	T	T	N	G	E	W	K	D	Q	S	V	V	D	K	Q	N	V	D	N	T	K	D	Q	N	L	S	M	N	G	I	I	L	L	N	Y	I	L	K	S	V	T	R	I	Y	V	A	L	F	A	L	T	R	A	Q	N
23	RVA/Human-wt/ZAF/UFS-NGS-MRC-DPRU1116/2004/G1P[8]	T	T	S	G	E	W	K	D	Q	N	V	V	D	R	Q	N	V	D	N	T	K	D	Q	N	L	S	T	N	G	I	I	L	L	N	Y	I	L	K	S	V	T	R	I	F	V	A	L	F	A	L	T	K	A	Q	N
24	RVA/Human-wt/ZAF/UFS-NGS-MRC-DPRU1131/2004/G1P[8]	T	T	S	G	E	W	K	D	Q	N	V	V	D	R	Q	N	V	D	N	T	K	D	Q	N	L	S	T	N	G	I	I	L	L	N	Y	I	L	K	S	V	T	R	I	F	V	A	L	F	A	L	T	K	A	Q	N
25	RVA/Human-wt/ZAF/UFS-NGS-MRC-DPRU1135/2004/G1P[8]	T	T	N	G	E	W	K	D	Q	S	V	V	D	K	Q	N	V	D	N	T	K	D	Q	N	L	S	M	N	G	I	I	L	L	N	Y	I	L	K	S	V	T	R	I	S	V	A	L	F	A	L	A	R	A	Q	N
26	RVA/Human-wt/ZAF/UFS-NGS-MRC-DPRU1139/2004/G1P[8]	T	T	N	G	E	W	K	D	Q	S	V	V	D	K	Q	N	V	D	N	T	K	D	Q	N	L	S	M	N	G	I	I	L	L	N	Y	I	L	K	S	V	T	R	I	S	V	A	L	F	A	L	A	R	A	Q	N
27	RVA/Human-wt/ZAF/UFS-NGS-MRC-DPRU1145/2004/G1P[8]	T	T	N	G	E	W	K	D	Q	S	V	V	D	K	Q	N	V	D	N	T	K	D	Q	N	L	S	M	N	G	I	I	L	L	N	Y	I	L	K	S	V	T	R	I	S	V	A	L	F	A	L	A	R	A	Q	N
28	RVA/Human-wt/ZAF/UFS-NGS-MRC-DPRU1148/2004/G1P[8]	T	T	S	G	E	W	K	D	Q	N	V	V	D	R	Q	N	V	D	N	T	K	D	Q	N	L	S	T	N	G	I	I	L	L	N	Y	I	L	K	S	V	T	R	I	F	V	A	L	F	A	L	T	K	A	Q	N
29	RVA/Human-wt/ZAF/UFS-NGS-MRC-DPRU1184/2004/G1P[8]	T	T	N	G	E	W	K	D	Q	S	V	V	D	K	Q	N	V	D	N	T	K	D	Q	N	L	S	M	N	G	I	I	L	L	N	Y	I	L	K	S	V	T	R	I	S	V	A	L	F	A	L	A	R	A	Q	N
30	RVA/Human-wt/ZAF/UFS-NGS-MRC-DPRU1192/2004/G1P[8]	T	T	N	G	E	W	K	D	Q	S	V	V	D	K	Q	N	V	D	N	T	K	D	Q	N	L	S	M	N	G	I	I	L	L	N	Y	I	L	K	S	V	T	R	I	S	V	A	L	F	A	L	A	R	A	Q	N
31	RVA/Human-wt/ZAF/UFS-NGS-MRC-DPRU1205/2004/G1P[8]	T	T	N	G	E	W	K	D	Q	S	V	V	D	K	Q	N	V	D	N	T	K	D	Q	N	L	S	M	N	G	I	I	L	L	N	Y	I	L	K	S	V	T	R	I	S	V	A	L	F	A	L	A	R	A	Q	N
32	RVA/Human-wt/ZAF/UFS-NGS-MRC-DPRU1230/2004/G1P[8]	T	T	N	G	E	W	K	D	Q	S	V	V	D	R	Q	N	V	D	N	T	K	D	Q	N	L	S	M	N	G	I	I	L	L	N	Y	I	L	K	S	V	T	R	I	S	V	A	L	F	A	L	T	R	A	Q	N
33	RVA/Human-wt/ZAF/UFS-NGS-MRC-DPRU1287/2004/G1P[8]	T	T	N	G	E	W	K	D	Q	S	V	V	D	K	Q	N	V	D	N	T	K	D	Q	N	L	S	M	N	G	I	I	L	L	N	Y	I	L	K	S	V	T	R	I	S	V	A	L	F	A	L	A	R	A	Q	N
34	RVA/Human-wt/ZAF/UFS-NGS-MRC-DPRU3410/2004/G1P[8]	T	T	N	G	E	W	K	D	Q	S	V	V	D	K	Q	N	V	D	N	T	K	D	Q	N	L	S	M	N	G	I	I	L	L	N	Y	I	L	K	S	V	T	R	I	S	V	A	L	F	A	L	A	R	A	Q	N
35	RVA/Human-wt/ZAFUFS-NGS-MRC-DPRU3449/2004/G1P[8]	T	T	N	G	E	W	K	D	Q	S	V	V	D	K	Q	N	V	D	N	T	K	D	Q	N	L	S	M	N	G	I	I	L	L	N	Y	I	L	K	S	V	T	R	I	S	V	A	L	F	A	L	A	R	A	Q	N
36	RVA/Human-wt/ZAF/UFS-NGS-MRC-DPRU451/2005/G1P[8]	T	T	N	G	E	W	K	D	Q	S	V	V	D	K	Q	N	V	D	N	T	K	D	Q	N	L	S	M	N	G	I	I	L	L	N	Y	I	L	K	S	V	T	R	I	S	V	A	L	F	A	L	A	R	A	Q	N
37	RVA/Human-wt/ZAF/UFS-NGS-MRC-DPRU658/2005/G1P[8]	T	T	S	G	E	W	K	D	Q	N	V	V	D	R	Q	N	V	D	N	T	K	D	Q	N	L	S	T	N	G	I	I	L	L	N	Y	I	L	K	S	V	T	R	I	F	V	A	L	F	A	L	T	K	A	Q	N
38	RVA/Human-wt/ZAF/UFS-NGS-MRC-DPRU682/2005/G1P[8]	T	T	N	G	E	W	K	D	Q	S	V	V	D	K	Q	N	V	D	N	T	K	D	Q	N	L	S	M	N	G	I	I	L	L	N	Y	I	L	K	S	V	T	R	I	S	V	A	L	F	A	L	T	R	A	Q	N
39	RVA/Human-wt/ZAF/UFS-NGS-MRC-DPRU687/2005/G1P[8]	T	T	N	G	E	W	K	D	Q	S	V	V	D	K	Q	N	V	D	N	T	K	D	Q	N	L	S	M	N	G	I	I	L	L	N	Y	I	L	K	S	V	T	R	I	Y	V	A	L	F	A	L	T	R	A	Q	N
40	RVA/Human-wt/ZAF/UFS-NGS-MRC-DPRU745/2005/G1P[8]	T	T	S	G	E	W	K	D	Q	N	V	V	D	K	Q	N	V	D	N	T	K	D	Q	N	L	S	T	N	G	I	I	L	L	N	Y	I	L	K	S	V	T	R	I	F	V	A	L	F	V	L	I	K	A	Q	N
41	RVA/Human-wt/ZAF/UFS-NGS-MRC-DPRU747/2005/G1P[8]	T	T	N	G	E	W	K	D	Q	S	V	V	D	K	Q	N	V	D	N	T	K	D	Q	N	L	S	M	N	G	I	I	L	L	N	Y	I	L	K	S	V	T	R	I	Y	V	A	L	F	A	L	T	R	A	Q	N
42	RVA/Human-wt/ZAF/UFS-NGS-MRC-DPRU732/2006/G1P[8]	T	T	S	G	E	W	K	D	Q	N	V	V	D	R	Q	N	V	D	N	T	K	D	Q	N	L	S	T	N	G	I	I	L	L	N	Y	I	L	K	S	V	T	R	I	F	V	A	L	F	A	L	T	K	A	Q	N
43	RVA/Human-wt/ZAF/UFS-NGS-MRC-DPRU749/2006/G1P[8]	T	T	S	G	E	W	K	D	Q	N	V	V	D	R	Q	N	V	D	N	T	K	D	Q	N	L	S	T	N	G	I	I	L	L	N	Y	I	L	K	S	V	T	R	I	F	V	A	L	F	A	L	T	K	A	Q	N
44	RVA/Human-wt/ZAF/UFS-NGS-MRC-DPRU753/2006/G1P[8]	T	T	S	G	E	W	K	D	Q	N	V	V	D	R	Q	N	V	D	N	T	K	D	Q	N	L	S	T	N	G	I	I	L	L	N	Y	I	L	K	S	V	T	R	I	F	V	A	L	F	A	L	T	K	A	Q	N
45	RVA/Human-wt/ZAF/UFS-NGS-MRC-DPRU757/2006/G1P[8]	T	T	S	G	E	W	K	D	Q	N	V	V	D	R	Q	N	V	D	N	T	K	D	Q	N	L	S	T	N	G	I	I	L	L	N	Y	I	L	K	S	V	T	R	I	F	V	A	L	F	A	L	T	K	A	Q	N
46	RVA/Human-wt/ZAF/UFS-NGS-MRC-DPRU768/2006/G1P[8]	T	T	S	G	E	W	K	D	Q	N	V	V	D	R	Q	N	V	D	N	T	K	D	Q	N	L	S	T	N	G	I	I	L	L	N	Y	I	L	K	S	V	T	R	I	F	V	A	L	F	A	L	T	K	A	Q	N
47	RVA/Human-wt/ZAF/UFS-NGS-MRC-DPRU783/2006/G1P[8]	T	T	S	G	E	W	K	D	Q	N	V	V	D	R	Q	N	V	D	N	T	K	D	Q	N	L	S	T	N	G	I	I	L	L	N	Y	I	L	K	S	V	T	R	I	F	V	A	L	F	A	L	T	K	A	Q	N
48	RVA/Human-wt/ZAF/UFS-NGS-MRC-DPRU849/2006/G1P[8]	T	T	S	G	E	W	K	D	Q	N	V	V	D	R	Q	N	V	D	N	T	K	D	Q	N	L	S	T	N	G	I	I	L	L	N	Y	I	L	K	S	V	T	R	I	F	V	A	L	F	A	L	T	K	A	Q	N
49	RVA/Human-wt/ZAF/UFS-NGS-MRC-DPRU902/2006/G1P[8]	T	T	S	G	E	W	K	D	Q	N	V	V	D	R	Q	N	V	D	N	T	K	D	Q	N	L	S	T	N	G	I	I	L	L	N	Y	I	L	K	S	V	T	R	I	F	V	A	L	F	A	L	T	K	A	Q	N
50	RVA/Human-wt/ZAF/UFS-NGS-MRC-DPRU981/2006/G1P[8]	T	T	S	G	E	W	K	E	Q	N	V	V	D	R	Q	N	V	D	N	T	K	D	Q	N	L	S	T	N	G	I	I	L	L	N	Y	I	L	K	S	V	T	R	I	S	V	A	L	F	A	L	T	K	A	Q	N
51	RVA/Human-wt/ZAF/UFS-NGS-MRC-DPRU999/2006/G1P[8]	T	T	S	G	E	W	K	D	Q	N	V	V	D	R	Q	N	V	D	N	T	K	D	Q	N	L	S	T	N	G	I	I	L	L	N	Y	I	L	K	S	V	T	R	I	F	V	A	L	F	A	L	T	K	A	Q	N
52	RVA/Human-wt/ZAF/UFS-NGS-MRC-DPRU1002/2006/G1P[8]	T	T	S	G	E	W	K	D	Q	N	V	V	D	R	Q	N	V	D	N	T	K	D	Q	N	L	S	T	N	G	I	I	L	L	N	Y	I	L	K	S	V	T	R	I	F	V	A	L	F	A	L	T	K	A	Q	N
53	RVA/Human-wt/ZAF/UFS-NGS-MRC-DPRU1005/2006/G1P[8]	T	T	S	G	E	W	K	D	Q	N	V	V	D	R	Q	N	V	D	N	T	K	D	Q	N	L	S	T	N	G	I	I	L	L	N	Y	I	L	K	S	V	T	R	I	F	V	A	L	F	A	L	T	K	A	Q	N
54	RVA/Human-wt/ZAF/UFS-NGS-MRC-DPRU1006/2006/G1P[8]	T	T	N	G	E	W	K	D	Q	S	V	V	D	K	Q	N	V	D	N	T	K	D	Q	N	L	S	M	N	G	I	I	L	L	N	Y	I	L	K	S	V	T	R	I	Y	V	A	L	F	A	L	T	R	A	Q	N
55	RVA/Human-wt/ZAF/UFS-NGS-MRC-DPRU1007/2006/G1P[8]	T	T	S	G	E	W	K	D	Q	N	V	V	D	R	Q	N	V	D	N	T	K	D	Q	N	L	S	T	N	G	I	I	L	L	N	Y	I	L	K	S	V	T	R	I	F	V	A	L	F	A	L	T	K	A	Q	N
56	RVA/Human-wt/ZAF/UFS-NGS-MRC-DPRU1009/2006/G1P[8]	T	T	N	G	E	W	K	D	Q	S	V	V	D	K	Q	N	V	D	N	T	K	D	Q	N	L	S	M	N	G	I	I	L	L	N	Y	I	L	K	S	V	T	R	I	Y	V	A	L	F	A	L	T	R	A	Q	N
57	RVA/Human-wt/ZAF/UFS-NGS-MRC-DPRU1012/2006/G1P[8]	T	T	S	G	E	W	K	D	Q	N	V	V	D	R	Q	N	V	D	N	T	K	D	Q	N	L	S	T	N	G	I	I	L	L	N	Y	I	L	K	S	V	T	R	I	F	V	A	L	F	A	L	T	K	A	Q	N
58	RVA/Human-wt/ZAF/UFS-NGS-MRC-DPRU1015/2006/G1P[8]	T	T	S	G	E	W	K	D	Q	N	V	V	D	R	Q	N	V	D	N	T	K	D	Q	N	L	S	T	N	G	I	I	L	L	N	Y	I	L	K	S	V	T	R	I	F	V	A	L	F	A	L	T	K	A	Q	N
59	RVA/Human-wt/ZAF/UFS-NGS-MRC-DPRU1019/2006/G1P[8]	T	T	N	G	E	W	K	D	Q	S	V	V	D	K	Q	N	V	D	N	T	K	D	Q	N	L	S	M	N	G	I	I	L	L	N	Y	I	L	K	S	V	T	R	I	Y	V	A	L	F	A	L	T	R	A	Q	N
60	RVA/Human-wt/ZAF/UFS-NGS-MRC-DPRU1030/2006/G1P[8]	T	T	S	G	E	W	K	D	Q	N	V	V	D	R	Q	N	V	D	N	T	K	D	Q	N	L	S	T	N	G	I	I	L	L	N	Y	I	L	K	S	V	T	R	I	F	V	A	L	F	A	L	T	K	A	Q	N
61	RVA/Human-wt/ZAF/UFS-NGS-MRC-DPRU1039/2006/G1P[8]	T	T	S	G	E	W	K	D	Q	N	V	V	D	R	Q	N	V	D	N	T	K	D	Q	N	L	S	T	N	G	I	I	L	L	N	Y	I	L	K	S	V	T	R	I	F	V	A	L	F	A	L	T	K	A	Q	N
62	RVA/Human-wt/ZAF/UFS-NGS-MRC-DPRU1041/2006/G1P[8]	T	T	S	G	E	W	K	D	Q	N	V	V	D	R	Q	N	V	D	N	T	K	D	Q	N	L	S	T	N	G	I	I	L	L	N	Y	I	L	K	S	V	T	R	I	F	V	A	L	F	A	L	T	K	A	Q	N
63	RVA/Human-wt/ZAF/UFS-NGS-MRC-DPRU3433/2006/G1P[8]	T	T	S	G	E	W	K	D	Q	N	V	V	D	R	Q	N	V	D	N	T	K	D	Q	N	L	S	T	N	G	I	I	L	L	N	Y	M	L	K	S	V	T	R	I	F	V	A	L	F	A	L	T	K	A	Q	N
64	RVA/Human-wt/ZAF/UFS-NGS-MRC-DPRU147/2007/G1P[8]	T	T	S	G	E	W	K	D	Q	N	V	V	D	R	Q	N	V	D	N	T	K	D	Q	N	L	S	T	N	G	I	I	L	L	N	Y	I	L	K	S	V	T	R	I	F	V	A	L	F	A	L	T	K	A	Q	N
65	RVA/Human-wt/ZAF/UFS-NGS-MRC-DPRU792/2007/G1P[8]	T	T	S	G	E	W	K	D	Q	N	V	V	D	R	Q	N	V	D	N	T	K	D	Q	N	L	S	T	N	G	I	I	L	L	N	Y	I	L	K	S	V	T	R	I	F	V	A	L	F	A	L	T	K	A	Q	N
66	RVA/Human-wt/ZAF/UFS-NGS-MRC-DPRU793/2007/G1P[8]	T	T	S	G	E	W	K	D	Q	N	V	V	D	R	Q	N	V	D	N	T	K	D	Q	N	L	S	T	N	G	I	I	L	L	N	Y	I	L	K	S	V	T	R	I	F	V	A	L	F	A	L	T	K	A	Q	N
67	RVA/Human-wt/ZAF/UFS-NGS-MRC-DPRU799/2007/G1P[8]	T	T	S	G	E	W	K	D	Q	N	V	V	D	R	Q	N	V	D	N	T	K	D	Q	N	L	S	T	N	G	I	I	L	L	N	Y	I	L	K	S	V	T	R	I	F	V	A	L	F	A	L	T	K	A	Q	N
68	RVA/Human-wt/ZAF/UFS-NGS-MRC-DPRU1273/2007/G1P[8]	T	T	S	G	E	W	K	D	Q	N	V	V	D	R	Q	N	V	D	N	T	K	D	Q	N	L	S	T	N	G	I	I	L	L	N	Y	I	L	K	S	V	T	R	I	F	V	A	L	F	A	L	T	K	A	Q	N
69	RVA/Human-wt/ZAF/UFS-NGS-MRC-DPRU1302/2007/G1P[8]	T	T	S	G	E	W	K	D	Q	N	V	V	D	R	Q	N	V	D	N	T	K	D	Q	N	L	S	T	N	G	I	I	L	L	N	Y	I	L	K	S	V	T	R	I	F	V	A	L	F	A	L	T	K	A	Q	N
70	RVA/Human-wt/ZAF/UFS-NGS-MRC-DPRU1652/2007/G1P[8]	T	T	S	G	E	W	K	D	Q	N	V	V	D	R	Q	N	V	D	N	T	K	D	Q	N	L	S	T	N	G	I	I	L	L	N	Y	I	L	K	S	V	T	R	I	F	V	A	L	F	A	L	T	K	A	Q	N
71	RVA/Human-wt/ZAF/UFS-NGS-MRC-DPRU1042/2008/G1P[8]	T	T	N	G	E	W	K	D	Q	S	V	V	D	K	Q	N	V	D	N	T	K	D	Q	N	L	S	M	N	G	I	I	L	L	N	Y	I	L	K	S	V	T	R	I	Y	V	A	L	F	A	L	T	R	A	Q	N
72	RVA/Human-wt/ZAF/UFS-NGS:MRC-DPRU1047/2008/G1P[8]	T	T	S	G	E	W	K	D	Q	N	V	V	D	R	Q	N	V	D	N	T	K	D	R	N	L	S	T	N	G	I	I	L	L	N	Y	I	L	K	S	V	T	R	I	F	V	A	L	F	A	L	T	K	A	Q	N
73	RVA/Human-wt/ZAF/UFS-NGS-MRC-DPRU1048/2008/G1P[8]	T	T	S	G	E	W	K	D	Q	N	V	V	D	R	Q	N	V	D	N	T	K	D	R	N	L	S	T	N	G	I	I	L	L	N	Y	I	L	K	S	V	T	R	I	F	V	A	L	F	A	L	T	K	A	Q	N
74	RVA/Human-wt/ZAF/UFS-NGS-MRC-DPRU1965/2008/G1P[8]	T	T	S	G	E	W	K	D	Q	N	V	V	D	R	Q	N	V	D	N	T	K	D	Q	N	L	S	T	N	G	I	I	L	L	N	Y	I	L	K	S	V	T	R	I	F	V	T	L	F	A	L	T	K	A	Q	N
75	RVA/Human-wt/ZAF/UFS-NGS-MRC-DPRU1967/2008/G1P[8]	T	T	S	G	E	W	K	D	Q	N	V	V	D	R	Q	N	V	D	N	T	K	D	Q	N	L	S	T	N	G	I	I	L	L	N	Y	I	L	K	S	V	T	R	I	F	V	A	L	F	A	L	T	K	A	Q	N
76	RVA/Human-wt/ZAF/UFS-NGS-MRC-DPRU1969/2008/G1P[8]	T	T	S	G	E	W	K	D	Q	N	V	V	D	R	Q	N	V	D	N	T	K	D	Q	N	L	S	T	N	G	I	I	L	L	N	Y	I	L	K	S	V	T	R	I	F	V	A	L	F	A	L	T	K	A	Q	N
77	RVA/Human-wt/ZAF/UFS-NGS-MRC-DPRU1970/2008/G1P[8]	T	T	S	G	E	W	K	D	Q	N	V	V	D	R	Q	N	V	D	N	T	K	D	Q	N	L	S	T	N	G	I	I	L	L	N	Y	I	L	K	S	V	T	R	I	F	V	A	L	F	A	L	T	K	A	Q	N
78	RVA/Human-wt/ZAF/UFS-NGS-MRC-DPRU1980/2008/G1P[8]	T	T	S	G	E	W	K	D	Q	N	V	V	D	R	Q	N	V	D	N	T	K	D	Q	N	L	S	T	N	G	I	I	L	L	N	Y	I	L	K	S	V	T	R	I	F	V	A	L	F	A	L	T	K	A	Q	N
79	RVA/Human-wt/ZAF/UFS-NGS-MRC-DPRU2006/2008/G1P[8]	T	T	S	G	E	W	K	D	Q	N	V	V	D	R	Q	N	V	D	N	T	K	D	Q	N	L	S	T	N	G	I	I	L	L	N	Y	I	L	K	S	V	T	R	I	F	V	A	L	F	A	L	T	K	A	Q	N
80	RVA/Human-wt/ZAF/UFS-NGS-MRC-DPRU136/2009/G1P[8]	T	T	S	G	E	W	K	D	Q	N	V	V	D	R	Q	N	V	D	N	T	K	D	Q	N	L	S	T	N	G	I	I	L	L	N	Y	I	L	K	S	V	T	R	I	F	V	A	L	F	A	L	T	K	A	Q	N
81	RVA/Human-wt/ZAF/UFS-NGS-MRC-DPRU138/2009/G1P[8]	T	T	N	G	E	W	K	D	Q	S	V	V	D	K	Q	N	V	D	N	T	K	D	Q	N	L	S	M	N	G	I	I	L	L	N	Y	I	L	K	S	V	T	R	I	S	V	A	L	F	A	L	T	R	A	Q	N
82	RVA/Human-wt/ZAF/UFS-NGS-MRC-DPRU139/2009/G1P[8]	T	T	S	G	E	W	K	D	Q	N	V	V	D	R	Q	N	V	D	N	T	K	D	Q	N	L	S	T	N	G	I	I	L	L	N	Y	I	L	K	S	V	T	R	I	F	V	A	L	F	A	L	T	K	A	Q	N
83	RVA/Human-wt/ZAF/UFS-NGS-MRC-DPRU151/2009/G1P[8]	T	T	N	G	E	W	K	D	Q	S	V	V	D	K	Q	N	V	D	N	T	K	D	Q	N	L	S	M	N	G	I	I	L	L	N	Y	I	L	K	S	V	T	R	I	S	V	A	L	F	A	L	T	R	A	Q	N
84	RVA/Human-wt/ZAF/UFS-NGS-MRC-DPRU157/2009/G1P[8]	T	T	N	G	E	W	K	D	Q	S	V	V	D	K	Q	N	V	D	N	T	K	D	Q	N	L	S	M	N	G	I	I	L	L	N	Y	I	L	K	S	V	T	R	I	S	V	A	L	F	A	L	T	R	A	Q	N
85	RVA/Human-wt/ZAF/UFS-NGS-MRC-DPRU1049/2009/G1P[8]	T	T	S	G	E	W	K	D	Q	N	V	V	D	R	Q	N	V	D	N	T	K	D	Q	N	L	S	T	N	G	I	I	L	L	N	Y	I	L	K	S	V	T	R	I	F	V	A	L	F	A	L	T	K	A	Q	N
86	RVA/Human-wt/ZAF/UFS-NGS-MRC-DPRU1093/2009/G1P[8]	T	T	S	G	E	W	K	D	Q	N	V	V	D	R	Q	N	V	D	N	T	K	D	Q	N	L	S	T	N	G	I	I	L	L	N	Y	I	L	K	S	V	T	R	I	F	V	A	L	F	A	L	T	K	A	Q	N
87	RVA/Human-wt/ZAF/UFS-NGS-MRC-DPRU1190/2009/G1P[8]	T	T	S	G	E	W	K	D	Q	N	V	V	D	R	Q	N	V	D	N	T	K	D	Q	N	L	S	T	N	G	I	I	L	L	N	Y	I	L	K	S	V	T	R	I	F	V	A	L	F	A	L	T	K	A	Q	N
88	RVA/Human-wt/ZAF/UFS-NGS-MRC-DPRU1234/2009/G1P[8]	T	T	N	G	E	W	K	D	Q	S	V	V	D	K	Q	N	V	D	N	T	K	D	Q	N	L	S	M	N	G	I	I	L	L	N	Y	I	L	K	S	V	T	R	I	S	V	A	L	F	A	L	T	R	A	Q	N
89	RVA/Human-wt/ZAF/UFS-NGS-MRC-DPRU1271/2009/G1P[8]	T	T	N	G	E	W	K	D	Q	S	V	V	D	K	Q	N	V	D	N	T	K	D	Q	N	L	S	M	N	G	I	I	L	L	N	Y	I	L	K	S	V	T	R	I	S	V	A	L	F	A	L	T	R	A	Q	N
90	RVA/Human-wt/ZAF/UFS-NGS-MRC-DPRU2280/2009/G1P[8]	T	T	S	G	E	W	K	D	Q	N	V	V	D	R	Q	N	V	D	N	T	K	D	Q	N	L	S	T	N	G	I	I	L	L	N	Y	I	L	K	S	V	T	R	I	F	V	A	L	F	A	L	T	K	A	Q	N
91	RVA/Human-wt/ZAF/UFS-NGS-MRC-DPRU2291/2009/G1P[8]	T	N	S	G	E	W	K	D	Q	N	V	V	D	R	Q	N	V	D	N	T	K	D	Q	N	L	S	T	N	G	I	I	L	L	N	Y	I	L	K	S	V	T	R	I	S	I	A	L	F	A	L	T	K	A	Q	N
92	RVA/Human-wt/ZAF/UFS-NGS-MRC-DPRU2299/2009/G1P[8]	T	T	N	G	E	W	K	D	Q	S	V	V	D	K	Q	N	V	D	N	T	K	D	Q	N	L	S	M	N	G	I	I	L	L	N	Y	I	L	K	S	V	T	R	I	S	V	A	L	F	A	L	T	R	A	Q	N
93	RVA/Human-wt/ZAF/MRC-DPRU4418/2002/G1P[8]	T	T	N	G	E	W	K	D	Q	S	V	V	D	K	Q	N	V	D	N	T	K	D	Q	N	L	S	M	N	G	I	I	L	L	N	Y	I	L	K	S	V	T	R	I	S	V	A	L	F	A	L	T	R	A	Q	N
94	RVA/Human-wt/ZAF/MRC-DPRU1192/2002/G1P[8]	T	T	N	G	E	W	K	D	Q	S	V	V	D	K	Q	N	V	D	N	T	K	D	Q	N	L	S	M	N	G	I	I	L	L	N	Y	I	L	K	S	V	T	R	I	S	V	A	L	F	A	L	A	R	A	Q	N
95	RVA/Human-wt/ZAF/MRC-DPRU6113/2002/G1P[8]	T	T	N	G	E	W	K	D	Q	S	V	V	D	K	Q	N	V	D	N	T	K	D	Q	N	L	S	M	N	G	I	I	L	L	N	Y	I	L	K	S	V	T	R	I	S	V	A	L	F	A	L	T	R	A	Q	N
96	RVA/Human-wt/ZAF/MRC-DPRU4381/2002/G1P[8]	T	T	N	G	E	W	K	D	Q	S	V	V	D	K	Q	N	V	D	N	T	K	D	Q	N	L	S	M	N	G	I	I	L	L	N	Y	I	L	K	S	V	T	R	I	S	V	A	L	F	A	L	T	R	A	Q	N
97	RVA/Human-wt/ZAF/MRC-DPRU400/2003/G1P[8]	T	T	N	G	E	W	K	D	Q	S	V	V	D	K	Q	N	V	D	N	T	K	D	Q	N	L	S	M	N	G	I	I	L	L	N	Y	I	L	K	S	V	T	R	I	S	V	A	L	F	A	L	T	R	A	Q	N
98	RVA/Human-wt/ZAF/MRC-DPRU2198/2003/G1P[8]	T	T	N	G	E	W	K	D	Q	S	V	V	D	K	Q	N	V	D	N	T	K	D	Q	N	L	S	M	N	G	I	I	L	L	N	Y	I	L	K	S	V	T	R	I	S	V	A	L	F	A	L	T	R	A	Q	N
99	RVA/Human-wt/ZAF/MRC-DPRU648/2003/G1P[8]	T	T	N	G	E	W	K	D	Q	S	V	V	D	K	Q	N	V	D	N	T	K	D	Q	N	L	S	M	N	G	I	I	L	L	N	Y	I	L	K	S	V	T	R	I	S	V	A	L	F	A	L	T	R	A	Q	N
100	RVA/Human-wt/ZAF/MRC-DPRU451/2003/G1P[8]	T	T	N	G	E	W	K	D	Q	S	V	V	D	K	Q	N	V	D	N	T	K	D	Q	N	L	S	M	N	G	I	I	L	L	N	Y	I	L	K	S	V	T	R	I	S	V	A	L	F	A	L	T	R	A	Q	N
101	RVA/Human-wt/ZAF/MRC-DPRU445/2003/G1P[8]	T	T	N	G	E	W	K	D	Q	S	V	V	D	K	Q	N	V	D	N	T	K	D	Q	N	L	S	M	N	G	I	I	L	L	N	Y	I	L	K	S	V	T	R	I	S	V	A	L	F	A	L	T	R	A	Q	N
102	RVA/Human-wt/ZAF/MRC-DPRU2218/2003/G1P[8]	T	T	N	G	E	W	K	D	Q	S	V	V	D	K	Q	N	V	D	N	T	K	D	Q	N	L	S	M	N	G	I	I	L	L	N	Y	I	L	K	S	V	T	R	I	S	V	A	L	F	A	L	T	R	A	Q	N
103	RVA/Human-wt/ZAF/MRC-DPRU566/2003/G1P[8]	T	T	N	G	E	W	K	D	Q	S	V	V	D	K	Q	N	V	D	N	T	K	D	Q	N	L	S	M	N	G	I	I	L	L	N	Y	I	L	K	S	V	T	R	I	S	V	A	L	F	A	L	T	R	A	Q	N
104	RVA/Human-wt/ZAF/MRC-DPRU539/2003/G1P[8]	T	T	N	G	E	W	K	D	Q	S	V	V	D	K	Q	N	V	D	N	T	K	D	Q	N	L	S	M	N	G	I	I	L	L	N	Y	I	L	K	S	V	T	R	I	S	V	A	L	F	A	L	T	R	A	Q	N
105	RVA/Human-wt/ZAF/MRC-DPRU426/2004/G1P[8]	T	T	N	G	E	W	K	D	Q	S	V	V	D	K	Q	N	V	D	N	T	K	D	Q	N	L	S	M	N	G	I	I	L	L	N	Y	I	L	K	S	V	T	R	I	S	V	A	L	F	A	L	A	R	A	Q	N
106	RVA/Human-wt/ZAF/MRC-DPRU1224/2004/G1P[8]	T	T	N	G	E	W	K	D	Q	S	V	V	D	K	Q	N	V	D	N	T	K	D	Q	N	L	S	M	N	G	I	I	L	L	N	Y	I	L	K	S	V	T	R	I	S	V	A	L	F	A	L	A	R	A	Q	N
107	RVA/Human-wt/ZAF/MRC-DPRU1285/2004/G1P[8]	T	T	N	G	E	W	K	D	Q	S	V	V	D	K	Q	N	V	D	N	T	K	D	Q	N	L	S	M	N	G	I	I	L	L	N	Y	I	L	K	S	V	T	R	I	S	V	A	L	F	A	L	A	R	A	Q	N
108	RVA/Human-wt/ZAF/MRC-DPRU1275/2004/G1P[8]	T	T	N	G	E	W	K	D	Q	S	V	V	D	K	Q	N	V	D	N	T	K	D	Q	N	L	S	M	N	G	I	I	L	L	N	Y	I	L	K	S	V	T	R	I	S	V	A	L	F	A	L	A	R	A	Q	N
109	RVA/Human-wt/ZAF/MRC-DPRU1264/2004/G1P[8]	T	T	N	G	E	W	K	D	Q	S	V	V	D	K	Q	N	V	D	N	T	K	D	Q	N	L	S	M	N	G	I	I	L	L	N	Y	I	L	K	S	V	T	R	I	S	V	A	L	F	A	L	A	R	A	Q	N
110	RVA/Human-wt/ZAF/MRC-DPRU1299/2004/G1P[8]	T	T	N	G	E	W	K	D	Q	S	V	V	D	K	Q	N	V	D	N	T	K	D	Q	N	L	S	M	N	G	I	I	L	L	N	Y	I	L	K	S	V	T	R	I	S	V	A	L	F	A	L	A	R	A	Q	N
111	RVA/Human-wt/ZAF/MRC-DPRU1283/2004/G1P[8]	T	T	N	G	E	W	K	D	Q	S	V	V	D	K	Q	N	V	D	N	T	K	D	Q	N	L	S	M	N	G	I	I	L	L	N	Y	I	L	K	S	V	T	R	I	S	V	A	L	F	A	L	A	R	A	Q	N
112	RVA/Human-wt/ZAF/MRC-DPRU1277/2004/G1P[8]	T	T	N	G	E	W	K	D	Q	S	V	V	D	K	Q	N	V	D	N	T	K	D	Q	N	L	S	M	N	G	I	I	L	L	N	Y	I	L	K	S	V	T	R	I	S	V	A	L	F	A	L	A	R	A	Q	N
113	RVA/Human-wt/ZAF/MRC-DPRU1280/2004/G1P[8]	T	T	N	G	E	W	K	D	Q	S	V	V	D	K	Q	N	V	D	N	T	K	D	Q	N	L	S	M	N	G	I	I	L	L	N	Y	I	L	K	S	V	T	R	I	S	V	A	L	F	A	L	A	R	A	Q	N
114	RVA/Human-wt/ZAF/MRC-DPRU822/2005/G1P[8]	T	T	S	G	E	W	K	D	Q	N	V	V	D	K	Q	N	V	D	N	T	K	D	Q	N	L	S	T	N	G	I	I	L	L	N	Y	I	L	K	S	V	T	R	I	F	V	A	L	F	V	L	I	K	A	Q	N
115	RVA/Human-wt/ZAF/MRC-DPRU2132/2005/G1P[8]	T	T	S	G	E	W	K	D	Q	N	V	V	D	R	Q	N	V	D	N	T	K	D	Q	N	L	L	T	N	G	I	I	L	L	N	Y	M	L	K	S	V	T	R	I	F	V	A	L	F	A	L	T	K	A	Q	N
116	RVA/Human-wt/ZAF/MRC-DPRU2114/2005/G1P[8]	T	T	S	G	E	W	K	D	Q	N	V	V	D	R	Q	N	I	D	N	T	K	D	Q	N	L	S	T	N	G	I	I	L	L	N	Y	I	L	K	S	V	T	R	I	F	V	A	L	F	A	L	T	K	A	Q	N
117	RVA/Human-wt/ZAF/MRC-DPRU799/2006/G1P[8]	T	T	S	G	E	W	K	D	Q	N	V	V	D	R	Q	N	V	D	N	T	K	D	Q	N	L	S	T	N	G	I	I	L	L	N	Y	I	L	K	S	V	T	R	I	F	V	A	L	F	A	L	T	K	A	Q	N
118	RVA/Human-wt/ZAF/MRC-DPRU789/2006/G1P[8]	T	T	S	G	E	W	K	D	Q	N	V	V	D	R	Q	N	V	D	N	T	K	D	Q	N	L	S	T	N	G	I	I	L	L	N	Y	I	L	K	S	V	T	R	I	F	V	A	L	F	A	L	T	K	A	Q	N
119	RVA/Human-wt/ZAF/MRC-DPRU748/2006/G1P[8]	T	T	S	G	E	W	K	D	Q	N	V	V	D	R	Q	N	V	D	N	T	K	D	Q	N	L	S	T	N	G	I	I	L	L	N	Y	I	L	K	S	V	T	R	I	F	V	A	L	F	A	L	T	K	A	Q	N
120	RVA/Human-wt/ZAF/MRC-DPRU839/2006/G1P[8]	T	T	S	G	E	W	K	D	Q	N	V	V	D	R	Q	N	V	D	N	T	K	D	Q	N	L	S	T	N	G	I	I	L	L	N	Y	I	L	K	S	V	T	R	I	F	V	A	L	F	A	L	T	K	A	Q	N
121	RVA/Human-wt/ZAF/MRC-DPRU757/2006/G1P[8]	T	T	S	G	E	W	K	D	Q	N	V	V	D	R	Q	N	V	D	N	T	K	D	Q	N	L	S	T	N	G	I	I	L	L	N	Y	I	L	K	S	V	T	R	I	F	V	A	L	F	A	L	T	K	A	Q	N
122	RVA/Human-wt/ZAF/MRC-DPRU832/2006/G1P[8]	T	T	S	G	E	W	K	D	Q	N	V	V	D	R	Q	N	V	D	N	T	K	D	Q	N	L	S	T	N	G	I	I	L	L	N	Y	I	L	K	S	V	T	R	I	F	V	A	L	F	A	L	T	K	A	Q	N
123	RVA/Human-wt/ZAF/MRC-DPRU901/2006/G1P[8]	T	T	S	G	E	W	K	D	Q	N	V	V	D	R	Q	N	V	D	N	T	K	D	Q	N	L	S	T	N	G	I	I	L	L	N	Y	I	L	K	S	V	T	R	I	F	V	A	L	F	A	L	T	K	A	Q	N
124	RVA/Human-wt/ZAF/MRC-DPRU840/2006/G1P[8]	T	T	S	G	E	W	K	D	Q	N	V	V	D	R	Q	N	V	D	N	T	K	D	Q	N	L	S	T	N	G	I	I	L	L	N	Y	I	L	K	S	V	T	R	I	F	V	A	L	F	A	L	T	K	A	Q	N
125	RVA/Human-wt/ZAF/MRC-DPRU803/2006/G1P[8]	T	T	S	G	E	W	K	D	Q	N	V	V	D	R	Q	N	V	D	N	T	K	D	Q	N	L	S	T	N	G	I	I	L	L	N	Y	I	L	K	S	V	T	R	I	F	V	A	L	F	A	L	T	K	A	Q	N
126	RVA/Human-wt/ZAF/MRC-DPRU1840/2007/G1P[8]	T	T	S	G	E	W	K	D	Q	N	V	V	D	R	Q	N	V	D	N	T	K	D	Q	N	L	S	T	N	G	I	I	L	L	N	Y	I	L	K	S	V	T	R	I	F	V	A	L	F	A	L	T	K	A	Q	N
127	RVA/Human-wt/ZAF/MRC-DPRU1327/2007/G1P[8]	T	T	S	G	E	W	K	D	Q	N	V	V	D	R	Q	N	V	D	N	T	K	D	Q	N	L	S	T	N	G	I	I	L	L	N	Y	I	L	K	S	V	T	R	I	F	V	A	L	F	A	L	T	K	A	Q	N
128	RVA/Human-wt/ZAF/MRC-DPRU1370/2007/G1P[8]	T	T	S	G	E	W	K	D	Q	N	V	V	D	R	Q	N	V	D	N	T	K	D	Q	N	L	S	T	N	G	I	I	L	L	N	Y	I	L	K	S	V	T	R	I	F	V	A	L	F	A	L	T	K	A	Q	N
129	RVA/Human-wt/ZAF/MRC-DPRU1350/2007/G1P[8]	T	T	S	G	E	W	K	D	Q	N	V	V	D	R	Q	N	V	D	N	T	K	D	Q	N	L	S	T	N	G	I	I	L	L	N	Y	I	L	K	S	V	T	R	I	F	V	A	L	F	A	L	T	K	A	Q	N
130	RVA/Human-wt/ZAF/MRC-DPRU1381/2007/G1P[8]	T	T	S	G	E	W	K	D	Q	N	V	V	D	R	Q	N	V	D	N	T	K	D	Q	N	L	S	T	N	G	I	I	L	L	N	Y	I	L	K	S	V	T	R	I	F	V	A	L	F	A	L	T	K	A	Q	N
131	RVA/Human-wt/ZAF/MRC-DPRU1960/2008/G1P[8]	T	T	S	G	E	W	K	D	Q	N	V	V	D	R	Q	N	V	D	N	T	K	D	Q	N	L	S	T	N	G	I	I	L	L	N	Y	I	L	K	S	V	T	R	I	F	V	A	L	F	A	L	T	K	A	Q	N
132	RVA/Human-wt/ZAF/MRC-DPRU1039/2008/G1P[8]	T	T	S	G	E	W	K	D	Q	N	V	V	D	R	Q	N	V	D	N	T	K	D	Q	N	L	S	T	N	G	I	I	L	L	N	Y	I	L	K	S	V	T	R	I	F	V	A	L	F	A	L	T	K	A	Q	N
133	RVA/Human-wt/ZAF/MRC-DPRU1052/2008/G1P[8]	T	T	S	G	E	W	K	D	Q	N	V	V	D	R	Q	N	V	D	N	T	K	D	Q	N	L	S	T	N	G	I	I	L	L	N	Y	I	L	K	S	V	T	R	I	F	V	A	L	F	A	L	T	K	A	Q	N
134	RVA/Human-wt/ZAF/MRC-DPRU2429/2008/G1P[8]	T	T	S	G	E	W	K	D	Q	N	V	V	D	R	Q	N	V	D	N	T	K	D	Q	N	L	S	T	N	G	I	I	L	L	N	Y	I	L	K	S	V	T	R	I	F	V	A	L	F	A	L	T	K	A	Q	N
135	RVA/Human-wt/ZAF/MRC-DPRU2125/2008/G1P[8]	T	T	S	G	E	W	K	D	Q	N	V	V	D	R	Q	N	V	D	N	T	K	D	Q	N	L	S	T	N	G	I	I	L	L	N	Y	I	L	K	S	V	T	R	I	F	V	A	L	F	A	L	T	K	A	Q	N
136	RVA/Human-wt/ZAF/MRC-DPRU1270/2009/G1P[8]	T	T	N	G	E	W	K	D	Q	S	V	V	D	K	Q	N	V	D	N	T	K	D	Q	N	L	S	M	N	G	I	I	L	L	N	Y	I	L	K	S	V	T	R	I	S	V	A	L	F	A	L	T	R	A	Q	N
137	RVA/Human-wt/ZAF/MRC-DPRU1389/2009/G1P[8]	T	T	N	G	E	W	K	D	Q	S	V	V	D	K	Q	N	V	D	N	T	K	D	Q	N	L	S	M	N	G	I	I	L	L	N	Y	I	L	K	S	V	T	R	I	S	V	A	L	F	A	L	T	R	A	Q	N
138	RVA/Human-wt/ZAF/MRC-DPRU2801/2009/G1P[8]	T	T	N	G	E	W	K	D	Q	S	V	V	D	K	Q	N	V	D	N	T	K	D	Q	N	L	S	M	N	G	I	I	L	L	N	Y	I	L	K	S	V	T	R	I	S	V	A	L	F	A	L	T	R	A	Q	N
139	RVA/Human-wt/ZAF/MRC-DPRU150/2009/G1P[8]	T	T	N	G	E	W	K	D	Q	S	V	V	D	K	Q	N	V	D	N	T	K	D	Q	N	L	S	M	N	G	I	I	L	L	N	Y	I	L	K	S	V	T	R	I	S	V	A	L	F	A	L	T	R	A	Q	N
140	RVA/Human-wt/ZAF/MRC-DPRU155/2009/G1P[8]	T	T	N	G	E	W	K	D	Q	S	V	V	D	K	Q	N	V	D	N	T	K	D	Q	N	L	S	M	N	G	I	I	L	L	N	Y	I	L	K	S	V	T	R	I	S	V	A	L	F	A	L	T	R	A	Q	N
141	RVA/Human-wt/ZAF/MRC-DPRU2294/2009/G1P[8]	T	T	N	G	E	W	K	D	Q	S	V	V	D	K	Q	N	V	D	N	T	K	D	Q	N	L	S	M	N	G	I	I	L	L	N	Y	I	L	K	S	V	T	R	I	S	V	A	L	F	A	L	T	R	A	Q	N
142	RVA/Human-wt/ZAF/MRC-DPRU1269/2009/G1P[8]	T	T	N	G	E	W	K	D	Q	S	V	V	D	K	Q	N	V	D	N	T	K	D	Q	N	L	S	M	N	G	I	I	L	L	N	Y	I	L	K	S	V	T	R	I	S	V	A	L	F	A	L	T	R	A	Q	N
143	RVA/Human-wt/ZAF/MRC-DPRU2325/2009/G1P[8]	T	T	S	G	E	W	K	D	Q	N	V	V	D	R	Q	N	V	D	N	T	K	D	Q	N	L	S	T	N	G	I	I	L	L	N	Y	I	L	K	S	V	T	R	I	F	V	A	L	F	A	L	T	K	A	Q	N
144	RVA/Human-wt/ZAF/MRC-DPRU2306/2009/G1P[8]	T	T	S	G	E	W	K	D	Q	N	V	V	D	R	Q	N	V	D	N	T	K	D	Q	N	L	S	T	N	G	I	I	L	L	N	Y	I	L	K	S	V	T	R	I	F	V	A	L	F	A	L	T	K	A	Q	N
145	RVA/Human-wt/ZAF/MRC-DPRU2330/2009/G1P[8]	T	T	S	G	E	W	K	D	Q	N	V	V	D	R	Q	N	V	D	N	T	K	D	Q	N	L	S	T	N	G	I	I	L	L	N	Y	I	L	K	S	V	T	R	I	F	V	A	L	F	A	L	T	K	A	Q	N
146	RVA/Human-wt/ZAF/MRC-DPRU1217/2009/G1P[8]	T	T	S	G	E	W	K	D	Q	N	V	V	D	R	Q	N	V	D	N	T	K	D	Q	N	L	S	T	N	G	I	I	L	L	N	Y	I	L	K	S	V	T	R	I	F	V	A	L	F	A	L	T	K	A	Q	N
147	RVA/Human-wt/ZAF/MRC-DPRU135/2009/G1P[8]	T	T	S	G	E	W	K	D	Q	N	V	V	D	R	Q	N	V	D	N	T	K	D	Q	N	L	S	T	N	G	I	I	L	L	N	Y	I	L	K	S	V	T	R	I	F	V	A	L	F	A	L	T	K	A	Q	N
148	RVA/Human-wt/ZAF/MRC-DPRU1923/2009/G1P[8]	T	T	S	G	E	W	K	D	Q	N	V	V	D	R	Q	N	I	D	N	T	K	D	Q	N	L	S	T	N	G	I	I	L	L	N	Y	I	L	K	S	V	T	R	I	F	V	A	L	F	A	L	T	K	A	Q	N
	South African post-vaccine strains																																																							
149	RVA/Human-wt/ZAF/UFS-NGS-MRC-DPRU2021/2010/G1P[8]	T	T	S	G	E	W	K	D	Q	N	V	V	D	R	Q	N	V	D	N	T	K	D	Q	N	L	S	T	N	G	I	I	L	L	N	Y	I	L	K	S	V	T	R	I	F	V	A	L	F	A	L	T	K	A	Q	N
150	RVA/Human-wt/ZAF/UFS-NGS-MRC-DPRU2250/2013/G1P[8]	T	T	N	G	E	W	K	D	Q	S	V	V	D	K	Q	N	V	D	N	T	K	D	Q	N	L	S	M	N	G	I	I	L	L	N	Y	I	L	K	S	V	T	R	I	Y	V	A	L	F	A	L	T	R	A	Q	N
151	RVA/Human-wt/ZAF/UFS-NGS-MRC-DPRU56/2014/G1P[8]	T	T	N	G	E	W	K	D	Q	S	V	V	D	K	Q	N	V	D	N	T	K	D	Q	D	L	S	M	N	G	I	I	L	L	N	Y	I	L	K	S	V	T	R	I	S	V	A	L	F	A	L	T	R	A	Q	N
152	RVA/Human-wt/ZAF/UFS-NGS-MRC-DPRU65/2014/G1P[8]	T	T	N	G	E	W	K	D	Q	S	V	V	D	K	Q	N	V	D	N	T	K	D	Q	D	L	S	M	N	G	I	I	L	L	N	Y	I	L	K	S	V	T	R	I	S	V	A	L	F	A	L	T	R	A	Q	N
153	RVA/Human-wt/ZAF/UFS-NGS-MRC-DPRU74/2014/G1P[8]	T	T	N	G	E	W	K	D	Q	S	V	V	D	K	Q	N	V	D	N	T	K	D	Q	D	L	S	M	N	G	I	I	L	L	N	Y	I	L	K	S	V	T	R	I	S	V	A	L	F	A	L	T	R	A	Q	N
154	RVA/Human-wt/ZAF/UFS-NGS-MRC-DPRU150/2014/G1P[8]	T	T	N	G	E	W	K	D	Q	S	V	V	D	K	Q	N	V	D	N	T	K	D	Q	D	L	S	M	N	G	I	I	L	L	N	Y	I	L	K	S	V	T	R	I	S	V	A	L	F	A	L	T	R	A	Q	N
155	RVA/Human-wt/ZAF/UFS-NGS-MRC-DPRU143/2014/G1P[8]	T	T	N	G	E	W	K	D	Q	S	V	V	D	K	Q	N	V	D	N	T	K	D	Q	D	L	S	M	N	G	I	I	L	L	N	Y	I	L	K	S	V	T	R	I	S	V	A	L	F	A	L	T	R	A	Q	N
156	RVA/Human-wt/ZAF/UFS-NGS-MRC-DPRU4347/2015/G1P[8]	T	T	N	G	E	W	K	D	Q	S	V	V	D	K	Q	N	V	D	N	T	K	D	Q	D	L	S	M	N	G	I	I	L	L	N	Y	I	L	K	S	V	T	R	I	S	V	V	L	F	A	L	T	R	A	Q	N
157	RVA/Human-wt/ZAF/UFS-NGS-MRC-DPRU4352/2015/G1P[8]	T	T	N	G	E	W	K	D	Q	S	V	V	D	K	Q	N	V	D	N	T	K	D	Q	D	L	S	M	N	G	I	I	L	L	N	Y	I	L	K	S	V	T	R	I	S	V	V	L	F	A	L	T	R	A	Q	N
158	RVA/Human-wt/ZAF/UFS-NGS-MRC-DPRU4357/2015/G1P[8]	T	N	N	G	E	W	K	D	Q	S	V	V	D	K	Q	N	V	D	N	T	K	D	Q	D	L	S	M	N	G	I	I	L	L	N	Y	I	L	K	S	V	T	R	I	S	V	A	L	F	A	L	T	R	A	Q	N
159	RVA/Human-wt/ZAF/UFS-NGS-MRC-DPRU15948/2017/G1P[8]	T	T	N	G	E	W	K	D	Q	S	V	V	D	K	Q	N	V	D	N	T	K	D	Q	N	L	S	M	N	G	I	I	L	L	N	Y	I	L	K	S	V	T	R	I	S	V	A	L	F	A	L	T	R	A	Q	N
160	RVA/Human-wt/ZAF/MRC-DPRU2030/2010/G1P[8]	T	T	S	G	E	W	K	D	Q	N	V	V	D	R	Q	N	V	D	N	T	K	D	Q	N	L	S	T	N	G	I	I	L	L	N	Y	I	L	K	S	V	T	R	I	F	V	A	L	F	A	L	T	K	A	Q	N
161	RVA/Human-wt/ZAF/MRC-DPRU2035/2010/G1P[8]	T	T	S	G	E	W	K	D	Q	N	V	V	D	R	Q	N	V	D	N	T	K	D	Q	N	L	S	T	N	G	I	I	L	L	N	Y	I	L	K	S	V	T	R	I	F	V	A	L	F	A	L	T	K	A	Q	N
162	RVA/Human-wt/ZAF/MRC-DPRU2052/2010/G1P[8]	T	T	S	G	E	W	K	D	Q	N	V	V	D	R	Q	N	V	D	N	T	K	D	Q	N	L	S	T	N	G	I	I	L	L	N	Y	I	L	K	S	V	T	R	I	F	V	A	L	F	A	L	T	K	A	Q	N
163	RVA/Human-wt/ZAF/MRC-DPRU1506/2010/G1P[8]	T	T	S	G	E	W	K	D	Q	N	V	V	D	R	Q	N	V	D	N	T	K	D	Q	N	L	S	T	N	G	I	I	L	L	N	Y	I	L	K	S	V	T	R	I	F	V	A	L	F	A	L	T	K	A	Q	N
164	RVA/Human-wt/ZAF/MRC-DPRU1544/2010/G1P[8]	T	T	S	G	E	W	K	D	Q	N	V	V	D	R	Q	N	V	D	N	A	K	D	Q	N	L	S	T	N	G	I	I	L	L	N	Y	I	L	K	S	V	T	R	I	F	V	A	L	F	A	L	T	K	A	Q	N
165	RVA/Human-wt/ZAF/MRC-DPRU2061/2010/G1P[8]	T	T	S	G	E	W	K	D	Q	N	V	V	D	R	Q	N	V	D	N	T	K	D	Q	N	L	S	T	N	G	I	I	L	L	N	Y	I	L	K	S	V	T	R	I	F	V	A	L	F	A	L	T	K	A	Q	N
166	RVA/Human-wt/ZAF/MRC-DPRU1492/2010/G1P[8]	T	T	S	G	E	W	K	D	Q	N	V	V	D	R	Q	N	V	D	N	T	K	D	Q	N	L	S	T	N	G	I	I	L	L	N	Y	I	L	K	S	V	T	R	I	F	V	A	L	F	A	L	T	K	A	Q	N
167	RVA/Human-wt/ZAF/MRC-DPRU83/2011/G1P[8]	T	T	N	G	E	W	K	D	Q	S	V	V	D	K	Q	N	V	D	N	T	K	D	Q	N	L	S	M	N	G	I	I	L	L	N	Y	I	L	K	S	V	T	R	I	S	V	A	L	F	A	L	T	R	A	Q	N
168	RVA/Human-wt/ZAF/MRC-DPRU6954/2011/G1P[8]	T	T	S	G	E	W	K	D	Q	N	V	V	D	R	Q	N	V	D	N	T	K	D	Q	N	L	S	T	N	G	I	I	L	L	N	Y	I	L	K	S	V	T	R	I	F	V	A	L	F	T	L	T	K	A	Q	N
169	RVA/Human-wt/ZAF/MRC-DPRU120/2011/G1P[8]	T	T	S	G	E	W	K	D	Q	N	V	V	D	R	Q	N	V	D	N	T	K	D	Q	N	L	S	T	N	G	I	I	L	L	N	Y	I	L	K	S	V	T	R	I	F	V	A	L	F	T	L	T	K	A	Q	N
170	RVA/Human-wt/ZAF/MRC-DPRU121/2011/G1P[8]	T	T	S	G	E	W	K	D	Q	N	V	V	D	R	Q	N	V	D	N	T	K	D	Q	N	L	S	T	N	G	I	I	L	L	N	Y	I	L	K	S	V	T	R	I	F	V	A	L	F	T	L	T	K	A	Q	N
171	RVA/Human-wt/ZAF/MRC-DPRU4079-11/2011/G1P[8]	T	T	S	G	E	W	K	D	Q	N	V	V	D	R	Q	N	V	D	N	T	K	D	Q	N	L	S	T	N	G	I	I	L	L	N	Y	I	L	K	S	V	T	R	I	F	V	A	L	F	T	L	T	K	A	Q	N

Figure S1: Alignment of VP7 neutralizing and cytotoxic T-cell lymphocytic epitopes residues of Rotarix® strain against wild type South African G1P[8] strains. Antigenic residues are divided in three epitopes (7-1a, 7-1b, and 7-2). Amino acids that differ with Rotarix® are colored in sky blue. Amino acid changes that have been shown to escape neutralization with monoclonal antibodies are indicated with a black dot. South Africa pre-vaccine G1 genotypes are indicated in boldface while post-vaccine strains are indicated in red.

		8-1											8-2		8-3									8-4			5-1								5-2	5-3	5-4	5-5
		•	•	•	•	•	•			•			•	•		•		•						•	•	•	•	•	•	•	•	•	•	•	•	•	•	•
		100	146	148	150	188	190	192	193	194	195	196	180	183	113	114	115	116	125	131	132	133	135	87	88	89	384	386	388	393	394	398	440	441	434	459	429	306
	Rotarix vaccine strain																																					
	RVA/Vaccine/USA/Rotarix-A41CB052A/1988/G1P[8]	D	S	Q	E	S	T	N	L	N	N	I	T	A	N	P	V	D	S	S	N	D	N	N	T	N	Y	F	I	W	P	G	R	T	P	E	L	R
	South African pre-vaccine strains																																					
1	RVA/Human-wt/ZAF/UFS-NGS-MRC-DPRU3883/2002/G1P[8]	D	S	Q	D	S	T	N	L	N	G	I	T	A	D	P	V	D	N	R	N	D	D	N	T	N	Y	F	I	W	P	G	R	T	P	E	L	R
2	RVA/Human-wt/ZAF/UFS-NGS-MRC-DPRU3956/2002/G1P[8]	D	S	Q	D	S	T	N	L	N	G	I	T	A	D	P	V	D	N	R	N	D	D	N	T	N	Y	F	I	W	P	G	R	T	P	E	L	R
3	RVA/Human-wt/ZAF/UFS-NGS-MRC-DPRU3892/2002/G1P[8]	D	S	Q	D	S	T	N	L	N	G	I	T	A	D	P	V	D	N	R	N	D	D	N	T	N	Y	F	I	W	P	G	R	T	P	E	L	R
4	RVA/Human-wt/ZAF/UFS-NGS-MRC-DPRU4049/2002/G1P[8]	D	S	Q	D	S	T	N	L	N	G	I	T	A	D	P	V	D	N	R	N	D	D	N	T	N	Y	F	I	W	P	G	R	T	P	E	L	R
5	RVA/Human-wt/ZAF/UFS-NGS-MRC-DPRU3951/2002/G1P[8]	D	S	Q	D	S	T	N	L	N	G	I	T	A	D	P	V	D	N	R	N	D	D	N	T	N	Y	F	I	W	P	G	R	T	P	E	L	R
6	RVA/Human-wt/ZAF/UFS-NGS-MRC-DPRU4018/2002/G1P[8]	D	S	Q	D	S	T	N	L	N	G	I	T	A	D	P	V	D	N	R	N	D	D	N	T	N	Y	F	I	W	P	G	R	T	P	E	L	R
7	RVA/Human-wt/ZAF/UFS-NGS-MRC-DPRU4019/2002/G1P[8]	D	S	Q	D	S	T	N	L	N	G	I	T	A	D	P	V	D	N	R	N	D	D	N	T	N	Y	F	I	W	P	G	R	T	P	E	L	R
8	RVA/Human-wt/ZAF/UFS-NGS-MRC-DPRU4055/2002/G1P[8]	D	S	Q	D	S	T	N	L	N	G	I	T	A	D	P	V	D	N	R	N	D	D	N	T	N	Y	F	I	W	P	G	R	T	P	E	L	R
9	RVA/Human-wt/ZAF/UFS-NGS-MRC-DPRU4271/2002/G1P[8]	D	S	Q	D	S	T	N	L	N	G	I	T	A	D	P	V	D	N	R	N	D	D	N	T	N	Y	F	I	W	P	G	R	T	P	E	L	R
10	RVA/Human-wt/ZAF/UFS-NGS-MRC-DPRU4102/2002/G1P[8]	D	S	Q	D	S	T	N	L	N	G	I	T	A	D	P	V	D	N	R	N	D	D	N	T	N	Y	F	I	W	P	G	R	T	P	E	L	R
11	RVA/Human-wt/ZAF/UFS-NGS-MRC-DPRU4023/2002/G1P[8]	D	S	Q	D	S	T	N	L	N	D	I	T	A	D	P	V	D	N	R	N	D	D	N	T	N	Y	F	I	W	P	G	R	T	P	E	L	R
12	RVA/Human-wt/ZAF/UFS-NGS-MRC-DPRU4269/2002/G1P[8]	D	S	Q	D	S	T	N	L	N	G	I	T	A	D	P	V	D	N	R	N	D	D	N	T	N	Y	F	I	W	P	G	R	T	P	E	L	R
13	RVA/Human-wt/ZAF/UFS-NGS-MRC-DPRU4024/2002/G1P[8]	D	S	Q	D	S	T	N	L	N	G	I	T	A	N	P	V	D	N	R	N	D	D	N	T	N	Y	F	I	W	P	G	R	T	P	E	L	R
14	RVA/Human-wt/ZAF/UFS-NGS-MRC-DPRU4026/2002/G1P[8]	D	S	Q	D	S	T	N	L	N	G	I	T	A	N	P	V	D	N	R	N	D	D	N	T	N	Y	F	I	W	P	G	R	T	P	E	L	R
15	RVA/Human-wt/ZAF/UFS-NGS-MRC-DPRU456/2003/G1P[8]	D	S	Q	D	S	T	N	L	N	G	I	T	A	D	P	V	D	N	R	N	D	D	N	T	N	Y	F	I	W	P	G	R	T	P	E	L	R
16	RVA/Human-wt/ZAF/UFS-NGS-MRC-DPRU493/2003/G1P[8]	D	S	Q	D	S	T	N	L	N	G	I	T	A	D	P	V	D	N	R	N	D	D	N	T	N	Y	F	I	W	P	G	R	T	P	E	L	R
17	RVA/Human-wt/ZAF/UFS-NGS-MRC-DPRU2156/2003/G1P[8]	D	S	Q	D	S	T	N	L	N	G	I	T	A	D	P	V	D	N	R	N	D	D	N	T	N	Y	F	I	W	P	G	R	T	P	E	L	R
18	RVA/Human-wt/ZAF/UFS-NGS-MRC-DPRU609/2003/G1P[8]	D	S	Q	D	S	T	N	L	N	G	I	T	A	D	P	V	D	N	R	N	D	D	N	T	N	Y	F	I	W	P	G	R	T	P	E	L	R
19	RVA/Human-wt/ZAF/UFS-NGS-MRC-DPRU632/2003/G1P[8]	D	S	Q	D	S	T	N	L	N	G	I	T	A	D	P	V	D	N	R	N	D	D	N	T	N	Y	F	I	W	P	G	R	T	P	E	L	R
20	RVA/Human-wt/ZAF/UFS-NGS-MRC-DPRU2145/2003/G1P[8]	D	S	Q	D	S	T	N	L	N	G	I	T	A	D	P	V	D	N	R	N	D	D	N	T	N	Y	F	I	W	P	G	R	T	P	E	L	R
21	RVA/Human-wt/ZAF/UFS-NGS-MRC-DPRU2117/2003/G1P[8]	D	S	Q	D	S	T	N	L	N	G	I	T	A	D	P	V	D	N	R	N	D	D	N	T	N	Y	F	I	W	P	G	R	T	P	E	L	R
22	RVA/Human-wt/ZAF/UFS-NGS-MRC-DPRU1230/2004/G1P[8]	D	S	Q	D	S	T	N	L	N	G	I	T	A	D	P	V	D	N	R	N	D	D	N	T	N	Y	F	I	W	P	G	R	T	P	E	L	R
23	RVA/Human-wt/ZAF/UFS-NGS-MRC-DPRU1135/2004/G1P[8]	D	S	Q	D	S	T	N	L	N	G	I	T	A	D	P	V	D	N	R	N	D	D	N	T	N	Y	F	I	W	P	G	R	T	P	E	L	R
24	RVA/Human-wt/ZAF/UFS-NGS-MRC-DPRU1184/2004/G1P[8]	D	S	Q	D	S	T	N	L	N	G	I	T	A	D	P	V	D	N	R	N	D	D	N	T	N	Y	F	I	W	P	G	R	T	P	E	L	R
25	RVA/Human-wt/ZAF/UFS-NGS-MRC-DPRU1192/2004/G1P[8]	D	S	Q	D	S	T	N	L	N	G	I	T	A	D	P	V	D	N	R	N	D	D	N	T	N	Y	F	I	W	P	G	R	T	P	E	L	R
26	RVA/Human-wt/ZAF/UFS-NGS-MRC-DPRU1205/2004/G1P[8]	D	S	Q	D	S	T	N	L	N	G	I	T	A	D	P	V	D	N	R	N	D	D	N	T	N	Y	F	I	W	P	G	R	T	P	E	L	R
27	RVA/Human-wt/ZAF/UFS-NGS-MRC-DPRU1139/2004/G1P[8]	D	S	Q	D	S	T	N	L	N	G	I	T	A	D	P	V	D	N	R	N	D	D	N	T	N	Y	F	I	W	P	G	R	T	P	E	L	R
28	RVA/Human-wt/ZAF/UFS-NGS-MRC-DPRU1145/2004/G1P[8]	D	S	Q	D	S	T	N	L	N	G	I	T	A	D	P	V	D	N	R	N	D	D	N	T	N	Y	F	I	W	P	G	R	T	P	E	L	R
29	RVA/Human-wt/ZAF/UFS-NGS-MRC-DPRU1104/2004/G1P[8]	D	S	Q	D	S	T	N	L	N	G	I	T	A	D	P	V	D	N	R	N	D	D	N	T	N	Y	F	I	W	P	G	R	T	P	E	L	R
30	RVA/Human-wt/ZAF/UFS-NGS-MRC-DPRU1116/2004/G1P[8]	D	S	Q	D	S	T	N	L	N	G	I	T	A	S	P	V	D	N	R	N	D	D	N	T	N	Y	F	I	W	P	G	R	T	P	E	L	R
31	RVA/Human-wt/ZAF/UFS-NGS-MRC-DPRU1131/2004/G1P[8]	D	S	Q	D	S	T	N	L	N	G	I	T	A	S	P	V	D	N	R	N	D	D	N	T	N	Y	F	I	W	P	G	R	T	P	E	L	R
32	RVA/Human-wt/ZAF/UFS-NGS-MRC-DPRU1148/2004/G1P[8]	D	S	Q	D	S	T	N	L	N	G	I	T	A	S	P	V	D	N	R	N	A	D	N	T	N	Y	F	I	W	P	G	R	T	P	E	L	R
33	RVA/Human-wt/ZAF/UFS-NGS-MRC-DPRU1287/2004/G1P[8]	D	S	Q	D	S	T	N	L	N	G	I	T	A	D	P	V	D	N	R	N	D	D	N	T	N	Y	F	I	W	P	G	R	T	P	E	L	R
34	RVA/Human-wt/ZAF/UFS-NGS-MRC-DPRU3410/2004/G1P[8]	D	S	Q	D	S	T	N	L	N	G	I	T	A	D	P	V	D	N	R	N	D	D	N	T	N	Y	F	I	W	P	G	R	T	P	E	L	R
35	RVA/Human-wt/ZAF/UFS-NGS-MRC-DPRU3449/2004/G1P[8]	D	S	Q	D	S	T	N	L	N	G	I	T	A	D	P	V	D	N	R	N	D	D	N	T	N	Y	F	I	W	P	G	R	T	P	E	L	R
36	RVA/Human-wt/ZAF/UFS-NGS-MRC-DPRU451/2005/G1P[8]	D	S	Q	D	S	T	N	L	N	G	I	T	A	D	P	V	D	N	R	N	D	D	N	T	N	Y	F	I	W	P	G	R	T	P	E	L	R
37	RVA/Human-wt/ZAF/UFS-NGS-MRC-DPRU687/2005/G1P[8]	D	S	Q	D	S	T	N	L	N	G	I	T	A	D	P	V	D	N	R	N	D	D	N	T	N	Y	F	I	W	P	G	R	T	P	E	L	R
38	RVA/Human-wt/ZAF/UFS-NGS-MRC-DPRU747/2005/G1P[8]	D	S	Q	D	S	T	N	L	N	G	I	T	A	D	P	V	D	N	R	N	D	D	N	T	N	Y	F	I	W	P	G	R	T	P	E	L	R
39	RVA/Human-wt/ZAF/UFS-NGS-MRC-DPRU745/2005/G1P[8]	D	S	Q	D	S	T	N	L	N	G	I	T	A	N	P	V	D	N	R	N	D	D	N	T	N	Y	F	I	W	P	G	R	T	P	E	L	R
40	RVA/Human-wt/ZAF/UFS-NGS-MRC-DPRU658/2005/G1P[8]	D	S	Q	D	S	T	N	L	N	G	I	T	A	N	P	V	D	N	R	N	D	D	N	T	N	Y	F	I	W	P	G	R	T	P	E	L	R
41	RVA/Human-wt/ZAF/UFS-NGS-MRC-DPRU682/2005/G1P[8]	D	N	Q	E	S	T	D	L	I	S	I	T	A	D	P	V	D	S	R	N	D	N	N	T	N	Y	F	L	W	P	G	R	T	P	D	L	R
42	RVA/Human-wt/ZAF/UFS-NGS-MRC-DPRU1006/2006/G1P[8]	D	S	Q	D	S	T	N	L	N	G	I	T	A	D	P	V	D	N	R	N	D	D	N	T	N	Y	F	I	W	P	G	R	T	P	E	L	R
43	RVA/Human-wt/ZAF/UFS-NGS-MRC-DPRU1009/2006/G1P[8]	D	S	Q	D	S	T	N	L	N	G	I	T	A	D	P	V	D	N	R	N	D	D	N	T	N	Y	F	I	W	P	G	R	T	P	E	L	R
44	RVA/Human-wt/ZAF/UFS-NGS-MRC-DPRU1019/2006/G1P[8]	D	S	Q	D	S	T	N	L	N	G	I	T	A	D	P	V	D	N	R	N	D	D	N	T	N	Y	F	I	W	P	G	R	T	P	E	L	R
45	RVA/Human-wt/ZAF/UFS-NGS-MRC-DPRU1039/2006/G1P[8]	D	S	Q	D	S	T	N	L	N	G	I	T	A	D	P	V	D	N	R	N	D	D	N	T	N	Y	F	I	W	P	G	R	T	P	E	L	R
46	RVA/Human-wt/ZAF/UFS-NGS-MRC-DPRU1007/2006/G1P[8]	D	S	Q	D	S	T	N	L	N	G	I	T	A	N	P	V	D	N	R	N	D	D	N	T	N	Y	F	I	W	P	G	R	T	P	E	L	R
47	RVA/Human-wt/ZAF/UFS-NGS-MRC-DPRU753/2006/G1P[8]	D	S	Q	D	S	T	N	L	N	G	I	T	A	N	P	V	D	N	R	N	D	D	N	T	N	Y	F	I	W	P	G	R	T	P	E	L	R
48	RVA/Human-wt/ZAF/UFS-NGS-MRC-DPRU732/2006/G1P[8]	D	S	Q	D	S	T	N	L	N	G	I	T	A	N	P	V	D	N	R	N	D	D	N	T	N	Y	F	I	W	P	G	R	T	P	E	L	R
49	RVA/Human-wt/ZAF/UFS-NGS-MRC-DPRU757/2006/G1P[8]	D	S	Q	D	S	T	N	L	N	G	I	T	A	N	P	V	D	N	R	N	D	D	N	T	N	Y	F	I	W	P	G	R	T	P	E	L	R
50	RVA/Human-wt/ZAF/UFS-NGS-MRC-DPRU768/2006/G1P[8]	D	S	Q	D	S	T	N	L	N	G	I	T	A	N	P	V	D	N	R	N	D	D	N	T	N	Y	F	I	W	P	G	R	T	P	E	L	R
51	RVA/Human-wt/ZAF/UFS-NGS-MRC-DPRU1005/2006/G1P[8]	D	S	Q	D	S	T	N	L	N	G	I	T	A	N	P	V	D	N	R	N	D	D	N	T	N	Y	F	I	W	P	G	R	T	P	E	L	R
52	RVA/Human-wt/ZAF/UFS-NGS-MRC-DPRU748/2006/G1P[8]	D	S	Q	D	S	T	N	L	N	G	I	T	A	N	P	V	D	N	R	N	D	D	D	T	N	Y	F	I	W	P	G	R	T	P	E	L	R
53	RVA/Human-wt/ZAF/UFS-NGS-MRC-DPRU901/2006/G1P[8]	D	S	Q	D	S	T	N	L	N	G	I	T	A	N	P	V	D	N	R	N	D	D	N	T	N	Y	F	I	W	P	G	R	T	P	E	L	R
54	RVA/Human-wt/ZAF/UFS-NGS-MRC-DPRU1012/2006/G1P[8]	D	S	Q	D	S	T	N	L	N	G	I	T	A	N	P	V	D	N	R	N	D	D	N	T	N	Y	F	I	W	P	G	R	T	P	E	L	R
55	RVA/Human-wt/ZAF/UFS-NGS-MRC-DPRU849/2006/G1P[8]	D	S	Q	D	S	T	N	L	N	G	I	T	A	N	P	V	D	N	R	N	D	D	N	T	N	Y	F	I	W	P	G	R	T	P	E	L	R
56	RVA/Human-wt/ZAF/UFS-NGS-MRC-DPRU783/2006/G1P[8]	D	S	Q	D	S	T	N	L	N	G	I	T	A	N	P	V	D	N	R	N	D	D	N	T	N	Y	F	I	W	P	G	R	T	P	E	L	R
57	RVA/Human-wt/ZAF/UFS-NGS-MRC-DPRU999/2006/G1P[8]	D	S	Q	D	S	T	N	L	N	G	I	T	A	N	P	V	D	N	R	N	D	D	N	T	N	Y	F	I	W	P	G	R	T	P	E	L	R
58	RVA/Human-wt/ZAF/UFS-NGS-MRC-DPRU1002/2006/G1P[8]	D	S	Q	D	S	T	N	L	N	G	I	T	A	N	P	V	D	N	R	N	D	D	N	T	N	Y	F	I	W	P	G	R	T	P	E	L	R
59	RVA/Human-wt/ZAF/UFS-NGS-MRC-DPRU1041/2006/G1P[8]	D	S	Q	D	S	T	N	L	N	G	I	T	A	N	P	V	D	N	R	N	D	D	N	T	N	Y	F	I	W	P	G	R	T	P	E	L	R
60	RVA/Human-wt/ZAF/UFS-NGS-MRC-DPRU1030/2006/G1P[8]	D	S	Q	D	S	T	N	L	N	G	I	T	A	N	P	V	D	N	R	N	D	D	N	T	N	Y	F	I	W	P	G	R	T	P	E	L	R
61	RVA/Human-wt/ZAF/UFS-NGS-MRC-DPRU1015/2006/G1P[8]	D	S	Q	D	S	T	N	L	N	G	I	T	A	D	P	V	D	N	R	N	D	D	N	T	N	Y	F	I	W	P	G	R	T	P	E	L	R
62	RVA/Human-wt/ZAF/UFS-NGS-MRC-DPRU981/2006/G1P[8]	D	S	Q	D	S	T	N	L	N	G	I	T	A	D	P	V	D	N	R	N	D	D	N	T	N	Y	F	I	W	P	G	R	T	P	E	L	R
63	RVA/Human-wt/ZAF/UFS-NGS-MRC-DPRU3433/2006/G1P[8]	D	S	Q	E	S	T	D	L	T	S	I	T	A	D	P	V	D	S	R	N	D	N	N	T	N	Y	F	L	W	P	G	R	T	P	D	L	R
64	RVA/Human-wt/ZAF/UFS-NGS-MRC-DPRU147/2007/G1P[8]	D	S	Q	D	S	T	N	L	N	G	I	T	A	N	P	V	D	N	R	N	D	D	N	T	N	Y	F	I	W	P	G	R	T	P	E	L	R
65	RVA/Human-wt/ZAF/UFS-NGS-MRC-DPRU1652/2007/G1P[8]	D	S	Q	D	S	T	N	L	N	G	I	T	A	N	P	V	D	N	R	N	D	D	N	T	N	Y	F	I	W	P	G	R	T	P	E	L	R
66	RVA/Human-wt/ZAF/UFS-NGS-MRC-DPRU1302/2007/G1P[8]	D	S	Q	D	S	T	N	L	N	G	I	T	A	N	P	V	D	N	R	N	D	D	N	T	N	Y	F	I	W	P	G	R	T	P	E	L	R
67	RVA/Human-wt/ZAF/UFS-NGS-MRC-DPRU792/2007/G1P[8]	D	S	Q	D	S	T	N	L	N	G	I	T	A	N	P	V	D	N	R	N	D	N	N	T	N	Y	F	I	W	P	G	R	T	P	E	L	R
68	RVA/Human-wt/ZAF/UFS-NGS-MRC-DPRU793/2007/G1P[8]	D	S	Q	D	S	T	N	L	N	G	I	T	A	N	P	V	D	N	R	N	D	D	N	T	N	Y	F	I	W	P	G	R	T	P	E	L	R
69	RVA/Human-wt/ZAF/UFS-NGS-MRC-DPRU1273/2007/G1P[8]	D	S	Q	D	S	T	N	L	N	G	I	T	A	N	P	V	D	N	R	N	D	D	N	T	N	Y	F	I	W	P	G	R	T	P	E	L	R
70	RVA/Human-wt/ZAF/UFS-NGS-MRC-DPRU799/2007/G1P[8]	D	S	Q	D	S	T	N	L	N	G	I	T	A	N	P	V	D	N	R	N	D	D	N	T	N	Y	F	I	W	P	G	R	T	P	E	L	R
71	RVA/Human-wt/ZAF/UFS-NGS-MRC-DPRU1042/2008/G1P[8]	D	S	Q	D	S	T	N	L	N	G	I	T	A	D	P	V	D	N	R	N	D	D	N	T	N	Y	F	I	W	P	G	R	T	P	E	L	R
72	RVA/Human-wt/ZAF/UFS-NGS-MRC-DPRU1967/2008/G1P[8]	D	S	Q	D	S	T	N	L	N	G	I	T	A	N	P	V	D	N	R	N	D	D	N	T	N	Y	F	I	W	P	G	R	T	P	E	L	R
73	RVA/Human-wt/ZAF/UFS-NGS:MRC-DPRU1047/2008/G1P[8]	D	S	Q	D	S	T	N	L	N	G	I	T	A	N	P	V	D	N	R	N	D	D	N	T	N	Y	F	I	W	P	G	R	T	P	E	L	R
74	RVA/Human-wt/ZAF/UFS-NGS-MRC-DPRU1965/2008/G1P[8]	D	S	Q	D	S	T	N	L	N	G	I	T	A	N	P	V	D	N	R	N	D	D	N	T	N	Y	F	I	W	P	G	R	T	P	E	L	R
75	RVA/Human-wt/ZAF/UFS-NGS-MRC-DPRU1969/2008/G1P[8]	D	S	Q	D	S	T	N	L	N	G	I	T	A	N	P	V	D	N	R	N	D	D	N	T	N	Y	F	I	W	P	G	R	T	P	E	L	R
76	RVA/Human-wt/ZAF/UFS-NGS-MRC-DPRU1048/2008/G1P[8]	D	S	Q	D	S	T	N	L	N	G	I	T	A	N	P	V	D	N	R	N	D	D	N	T	N	Y	F	I	W	P	G	R	T	P	E	L	R
77	RVA/Human-wt/ZAF/UFS-NGS-MRC-DPRU2006/2008/G1P[8]	D	S	Q	D	S	T	N	L	N	G	I	T	A	N	P	V	D	N	R	N	D	D	N	T	N	Y	F	I	W	P	G	R	T	P	E	L	R
78	RVA/Human-wt/ZAF/UFS-NGS-MRC-DPRU1970/2008/G1P[8]	D	S	Q	D	S	T	N	L	N	G	I	T	A	D	P	V	D	N	R	N	D	D	N	T	N	Y	F	I	W	P	G	R	T	P	E	L	R
79	RVA/Human-wt/ZAF/UFS-NGS-MRC-DPRU1980/2008/G1P[8]	D	S	Q	D	S	T	N	L	N	G	I	T	A	D	P	V	D	N	R	N	D	D	N	T	N	Y	F	I	W	P	G	R	T	P	E	L	R
80	RVA/Human-wt/ZAF/UFS-NGS-MRC-DPRU2280/2009/G1P[8]	D	S	Q	D	S	T	N	L	N	G	I	T	A	N	P	V	D	N	R	N	D	D	N	T	N	Y	F	I	W	P	G	R	T	P	E	L	R
81	RVA/Human-wt/ZAF/UFS-NGS-MRC-DPRU1049/2009/G1P[8]	D	S	Q	D	S	T	N	L	N	G	I	T	A	N	P	V	D	N	R	N	D	D	N	T	N	Y	F	I	W	P	G	R	T	P	E	L	R
82	RVA/Human-wt/ZAF/UFS-NGS-MRC-DPRU139/2009/G1P[8]	D	S	Q	D	S	T	N	L	N	G	I	T	A	N	P	V	D	N	R	N	D	D	N	T	N	Y	F	I	W	P	G	R	T	P	E	L	R
83	RVA/Human-wt/ZAF/UFS-NGS-MRC-DPRU1190/2009/G1P[8]	D	S	Q	D	S	T	N	L	N	G	I	T	A	N	P	V	D	N	R	N	D	D	N	T	N	Y	F	I	W	P	G	R	T	P	E	L	R
84	RVA/Human-wt/ZAF/UFS-NGS-MRC-DPRU1093/2009/G1P[8]	D	S	Q	D	S	T	N	L	N	G	I	T	A	N	P	V	D	N	R	N	D	D	N	T	N	Y	F	I	W	P	G	R	T	P	E	L	R
85	RVA/Human-wt/ZAF/UFS-NGS-MRC-DPRU138/2009/G1P[8]	D	G	Q	D	S	T	N	L	N	G	I	T	A	D	P	V	D	N	R	N	D	D	N	T	N	Y	F	I	W	P	G	R	T	P	E	L	R
86	RVA/Human-wt/ZAF/UFS-NGS-MRC-DPRU157/2009/G1P[8]	D	G	Q	D	S	T	N	L	N	G	I	T	A	D	P	V	D	N	R	N	D	D	N	T	N	Y	F	I	W	P	G	R	T	P	E	L	R
87	RVA/Human-wt/ZAF/UFS-NGS-MRC-DPRU150/2009/G1P[8]	D	G	Q	D	S	T	N	L	N	G	I	T	A	D	P	V	D	N	R	N	D	D	N	T	N	Y	F	I	W	P	G	R	T	P	E	L	R
88	RVA/Human-wt/ZAF/UFS-NGS-MRC-DPRU1234/2009/G1P[8]	D	G	Q	D	S	T	N	L	N	G	I	T	A	D	P	V	D	N	R	N	D	D	N	T	N	Y	F	I	W	P	G	R	T	P	E	L	R
89	RVA/Human-wt/ZAF/UFS-NGS-MRC-DPRU2299/2009/G1P[8]	D	G	Q	D	S	T	N	L	N	G	I	T	A	D	P	V	D	N	R	N	D	D	N	T	N	Y	F	I	W	P	G	R	T	P	E	L	R
90	RVA/Human-wt/ZAF/UFS-NGS-MRC-DPRU136/2009/G1P[8]	D	S	Q	D	S	T	N	L	N	G	I	T	A	D	P	V	D	N	R	N	D	D	N	T	N	Y	F	I	W	P	G	R	T	P	E	L	R
91	RVA/Human-wt/ZAF/UFS-NGS-MRC-DPRU1270/2009/G1P[8]	D	G	Q	D	S	T	N	L	N	G	I	T	A	D	P	V	D	N	R	N	D	D	N	T	N	Y	F	I	W	P	G	R	T	P	E	L	R
92	RVA/Human-wt/ZAF/UFS-NGS-MRC-DPRU2291/2009/G1P[8]	D	S	Q	D	S	T	N	L	N	G	I	T	A	D	P	V	D	N	R	N	D	D	N	T	N	Y	F	I	W	P	G	R	T	P	E	L	R
93	RVA/Human-wt/ZAF/MRC-DPRU6113/2002/G1P[8]	D	S	Q	D	S	T	N	L	N	G	I	T	A	D	P	V	D	N	R	N	D	D	N	T	N	Y	F	I	W	P	G	R	T	P	E	L	R
94	RVA/Human-wt/ZAF/MRC-DPRU4381/2002/G1P[8]	D	S	Q	D	S	T	N	L	N	G	I	T	A	D	P	V	D	N	R	N	D	D	N	T	N	Y	F	I	W	P	G	R	T	P	E	L	R
95	RVA/Human-wt/ZAF/MRC-DPRU4418/2002/G1P[8]	D	S	Q	D	S	T	N	L	N	G	I	T	A	D	P	V	D	N	R	N	D	D	N	T	N	Y	F	I	W	P	G	R	T	P	E	L	R
96	RVA/Human-wt/ZAF/MRC-DPRU1192/2002/G1P[8]	D	S	Q	D	S	T	N	L	N	G	I	T	A	D	P	V	D	N	R	N	D	D	N	T	N	Y	F	I	W	P	G	R	T	P	E	L	R
97	RVA/Human-wt/ZAF/MRC-DPRU566/2003/G1P[8]	D	S	Q	D	S	T	N	L	N	G	I	T	A	D	P	V	D	N	R	N	D	D	N	T	N	Y	F	I	W	P	G	R	T	P	E	L	R
98	RVA/Human-wt/ZAF/MRC-DPRU451/2003/G1P[8]	D	S	Q	D	S	T	N	L	N	G	I	T	A	D	P	V	D	N	R	N	D	D	N	T	N	Y	F	I	W	P	G	R	T	P	E	L	R
99	RVA/Human-wt/ZAF/MRC-DPRU445/2003/G1P[8]	D	S	Q	D	S	T	N	L	N	G	I	T	A	D	P	V	D	N	R	N	D	D	N	T	N	Y	F	I	W	P	G	R	T	P	E	L	R
100	RVA/Human-wt/ZAF/MRC-DPRU400/2003/G1P[8]	D	S	Q	D	S	T	N	L	N	G	I	T	A	D	P	V	D	N	R	N	D	D	N	T	N	Y	F	I	W	P	G	R	T	P	E	L	R
101	RVA/Human-wt/ZAF/MRC-DPRU2198/2003/G1P[8]	D	S	Q	D	S	T	N	L	N	G	I	T	A	D	P	V	D	N	R	N	D	D	N	T	N	Y	F	I	W	P	G	R	T	P	E	L	R
102	RVA/Human-wt/ZAF/MRC-DPRU2218/2003/G1P[8]	D	S	Q	D	S	T	N	L	N	G	I	T	A	N	P	V	D	N	R	N	D	D	N	T	N	F	F	I	W	P	G	R	T	P	E	L	R
103	RVA/Human-wt/ZAF/MRC-DPRU539/2003/G1P[8]	D	S	Q	D	S	T	N	L	N	G	I	T	A	D	P	V	D	N	R	N	D	D	N	T	N	Y	F	I	W	P	G	R	T	P	E	L	R
104	RVA/Human-wt/ZAF/MRC-DPRU648/2003/G1P[8]	D	S	Q	D	S	T	N	L	N	G	I	T	A	D	P	V	D	N	R	N	D	D	N	T	N	Y	F	I	W	P	G	R	T	P	E	L	R
105	RVA/Human-wt/ZAF/MRC-DPRU426/2004/G1P[8]	D	S	Q	D	S	T	N	L	N	G	I	T	A	D	P	V	D	N	R	N	D	D	N	T	N	Y	F	I	W	P	G	R	T	P	E	L	R
106	RVA/Human-wt/ZAF/MRC-DPRU1224/2004/G1P[8]	D	S	Q	D	S	T	N	L	N	G	I	T	A	D	P	V	D	N	R	N	D	D	N	T	N	Y	F	I	W	P	G	R	T	P	E	L	R
107	RVA/Human-wt/ZAF/MRC-DPRU1285/2004/G1P[8]	D	S	Q	D	S	T	N	L	N	G	I	T	A	D	P	V	D	N	R	N	D	D	N	T	N	Y	F	I	W	P	G	R	T	P	E	L	R
108	RVA/Human-wt/ZAF/MRC-DPRU1275/2004/G1P[8]	D	S	Q	D	S	T	N	L	N	G	I	T	A	D	P	V	D	N	R	N	D	D	N	T	N	Y	F	I	W	P	G	R	T	P	E	L	R
109	RVA/Human-wt/ZAF/MRC-DPRU1264/2004/G1P[8]	D	S	Q	D	S	T	N	L	N	G	I	T	A	D	P	V	D	N	R	N	D	D	N	T	N	Y	F	I	W	P	G	R	T	P	E	L	R
110	RVA/Human-wt/ZAF/MRC-DPRU1299/2004/G1P[8]	D	S	Q	D	S	T	N	L	N	G	I	T	A	D	P	V	D	N	R	N	D	D	N	T	N	Y	F	I	W	P	G	R	T	P	E	L	R
111	RVA/Human-wt/ZAF/MRC-DPRU1280-04/2004/G1P[8]	D	S	Q	D	S	T	N	L	N	G	I	T	A	D	P	V	D	N	R	N	D	D	N	T	N	Y	F	I	W	P	G	R	T	P	E	L	R
112	RVA/Human-wt/ZAF/MRC-DPRU1277/2004/G1P[8]	D	S	Q	D	S	T	N	L	N	G	I	T	A	D	P	V	D	N	R	N	D	D	N	T	N	Y	F	I	W	P	G	R	T	P	E	L	R
113	RVA/Human-wt/ZAF/MRC-DPRU1283/2004/G1P[8]	D	S	Q	D	S	T	N	L	N	G	I	T	A	D	P	V	D	N	R	N	D	D	N	T	N	Y	F	I	W	P	G	R	T	P	E	L	R
114	RVA/Human-wt/ZAF/MRC-DPRU822/2005/G1P[8]	D	S	Q	D	S	T	N	L	N	G	I	T	A	N	P	V	D	N	R	N	D	D	N	T	N	Y	F	I	W	P	G	R	T	P	E	L	R
115	RVA/Human-wt/ZAF/MRC-DPRU2132/2005/G1P[8]	D	S	Q	E	S	T	D	L	T	S	I	T	A	D	P	V	D	S	R	N	D	N	N	T	N	Y	F	L	W	P	G	R	T	P	D	L	R
116	RVA/Human-wt/ZAF/MRC-DPRU2114/2005/G1P[8]	D	S	Q	E	S	T	D	L	T	S	I	T	A	D	P	V	D	S	R	N	D	N	N	T	N	Y	F	L	W	P	G	R	T	P	D	L	R
117	RVA/Human-wt/ZAF/MRC-DPRU799/2006/G1P[8]	D	S	Q	D	S	T	N	L	N	G	I	T	A	N	P	V	D	N	R	N	D	D	N	T	N	Y	F	I	W	P	G	R	T	P	E	L	R
118	RVA/Human-wt/ZAF/MRC-DPRU757/2006/G1P[8]	D	S	Q	D	S	T	N	L	N	G	I	T	A	N	P	V	D	N	R	N	D	D	N	T	N	Y	F	I	W	P	G	R	T	P	E	L	R
119	RVA/Human-wt/ZAF/MRC-DPRU832/2006/G1P[8]	D	S	Q	D	S	T	N	L	N	G	I	T	A	N	P	V	D	N	R	N	D	D	N	T	N	Y	F	I	W	P	G	R	T	P	E	L	R
120	RVA/Human-wt/ZAF/MRC-DPRU789/2006/G1P[8]	D	S	Q	D	S	T	N	L	N	G	I	T	A	N	P	V	D	N	R	N	D	D	N	T	N	Y	F	I	W	P	G	R	T	P	E	L	R
121	RVA/Human-wt/ZAF/MRC-DPRU803/2006/G1P[8]	D	S	Q	D	S	T	N	L	N	G	I	T	A	N	P	V	D	N	R	N	D	D	N	T	N	Y	F	I	W	P	G	R	T	P	E	L	R
122	RVA/Human-wt/ZAF/MRC-DPRU840/2006/G1P[8]	D	S	Q	D	S	T	N	L	N	G	I	T	A	N	P	V	D	N	R	N	D	D	N	T	N	Y	F	I	W	P	G	R	T	P	E	L	R
123	RVA/Human-wt/ZAF/MRC-DPRU748/2006/G1P[8]	D	S	Q	D	S	T	N	L	N	G	I	T	A	N	P	V	D	N	R	N	D	D	D	T	N	Y	F	I	W	P	G	R	T	P	E	L	R
124	RVA/Human-wt/ZAF/MRC-DPRU839/2006/G1P[8]	D	S	Q	D	S	T	N	L	N	G	I	T	A	N	P	V	D	N	R	N	D	D	D	T	N	Y	F	I	W	P	G	R	T	P	E	L	R
125	RVA/Human-wt/ZAF/MRC-DPRU901/2006/G1P[8]	D	S	Q	D	S	T	N	L	N	G	I	T	A	N	P	V	D	N	R	N	D	D	N	T	N	Y	F	I	W	P	G	R	T	P	E	L	R
126	RVA/Human-wt/ZAF/MRC-DPRU1370/2007/G1P[8]	D	S	Q	D	S	T	N	L	N	G	I	T	A	N	P	V	D	N	R	N	D	D	N	T	N	Y	F	I	W	P	G	R	T	P	E	L	R
127	RVA/Human-wt/ZAF/MRC-DPRU1350/2007/G1P[8]	D	S	Q	D	S	T	N	L	N	G	I	T	A	N	P	V	D	N	R	N	D	D	N	T	N	Y	F	I	W	P	G	R	T	P	E	L	R
128	RVA/Human-wt/ZAF/MRC-DPRU1381/2007/G1P[8]	D	S	Q	D	S	T	N	L	N	G	I	T	A	N	P	V	D	N	R	N	D	D	N	T	N	Y	F	I	W	P	G	R	T	P	E	L	R
129	RVA/Human-wt/ZAF/MRC-DPRU1327/2007/G1P[8]	D	S	Q	D	S	T	N	L	N	G	I	T	A	N	P	V	D	N	R	N	D	D	N	T	N	Y	F	I	W	P	G	R	T	P	E	L	R
130	RVA/Human-wt/ZAF/MRC-DPRU1840/2007/G1P[8]	D	S	Q	E	S	T	D	L	T	S	I	T	A	D	P	V	D	S	R	N	D	N	N	T	N	Y	F	L	W	P	G	R	T	P	D	L	R
131	RVA/Human-wt/ZAF/MRC-DPRU1052/2008/G1P[8]	D	S	Q	D	S	T	N	L	N	G	I	T	A	N	P	V	D	N	R	N	D	D	N	T	N	Y	F	I	W	P	G	R	T	P	E	L	R
132	RVA/Human-wt/ZAF/MRC-DPRU2125/2008/G1P[8]	D	S	Q	D	S	T	N	L	N	G	I	T	A	N	P	V	D	N	R	N	D	D	N	T	N	Y	F	I	W	P	G	R	T	P	E	L	R
133	RVA/Human-wt/ZAF/MRC-DPRU2429/2008/G1P[8]	D	S	Q	D	S	T	N	L	N	G	I	T	A	N	P	V	D	N	R	N	D	D	N	T	N	Y	F	I	W	P	G	R	T	P	E	L	R
134	RVA/Human-wt/ZAF/MRC-DPRU1039/2008/G1P[8]	D	S	Q	D	S	T	N	L	N	G	I	T	A	N	P	V	D	N	R	N	D	D	N	T	N	Y	F	I	W	P	G	R	T	P	E	L	R
135	RVA/Human-wt/ZAF/MRC-DPRU1960/2008/G1P[8]	D	S	Q	D	S	T	N	L	N	G	I	T	A	N	P	V	D	N	R	N	D	D	N	T	N	Y	F	I	W	P	G	R	T	P	E	L	R
136	RVA/Human-wt/ZAF/MRC-DPRU1217/2009/G1P[8]	D	S	Q	D	S	T	N	L	N	S	I	T	A	D	P	V	D	N	R	N	D	D	N	T	N	Y	F	I	W	P	G	R	T	P	E	L	R
137	RVA/Human-wt/ZAF/MRC-DPRU2330/2009/G1P[8]	D	S	Q	D	S	T	N	L	N	G	I	T	A	N	P	V	D	N	R	N	D	D	N	T	N	Y	F	I	W	P	G	R	T	P	E	L	R
138	RVA/Human-wt/ZAF/MRC-DPRU2325/2009/G1P[8]	D	S	Q	D	S	T	N	L	N	G	I	T	A	N	P	V	D	N	R	N	D	D	N	T	N	Y	F	I	W	P	G	R	T	P	E	L	R
139	RVA/Human-wt/ZAF/MRC-DPRU2306/2009/G1P[8]	D	S	Q	D	S	T	N	L	N	G	I	T	A	N	P	V	D	N	R	N	D	D	N	T	N	Y	F	I	W	P	G	R	T	P	E	L	R
140	RVA/Human-wt/ZAF/MRC-DPRU1389/2009/G1P[8]	D	G	Q	D	S	T	N	L	N	G	I	T	A	D	P	V	D	N	R	N	D	D	N	T	N	Y	F	I	W	P	G	R	T	P	E	L	R
141	RVA/Human-wt/ZAF/MRC-DPRU1270/2009/G1P[8]	D	G	Q	D	S	T	N	L	N	G	I	T	A	D	P	V	D	N	R	N	D	D	N	T	N	Y	F	I	W	P	G	R	T	P	E	L	R
142	RVA/Human-wt/ZAF/MRC-DPRU2294/2009/G1P[8]	D	G	Q	D	S	T	N	L	N	G	I	T	A	D	P	V	D	N	R	N	D	D	N	T	N	Y	F	I	W	P	G	R	T	P	E	L	R
143	RVA/Human-wt/ZAF/MRC-DPRU1269/2009/G1P[8]	D	G	Q	D	S	T	N	L	N	G	I	T	A	D	P	V	D	N	R	N	D	D	N	T	N	Y	F	I	W	P	G	R	T	P	E	L	R
144	RVA/Human-wt/ZAF/MRC-DPRU2801/2009/G1P[8]	D	G	Q	D	S	T	N	L	N	G	I	T	A	D	P	V	D	N	R	N	D	D	N	T	N	Y	F	I	W	P	G	R	T	P	E	L	R
145	RVA/Human-wt/ZAF/MRC-DPRU150/2009/G1P[8]	D	G	Q	D	S	T	N	L	N	G	I	T	A	D	P	V	D	N	R	N	D	D	N	T	N	Y	F	I	W	P	G	R	T	P	E	L	R
146	RVA/Human-wt/ZAF/MRC-DPRU155/2009/G1P[8]	D	G	Q	D	S	T	N	L	N	G	I	T	A	D	P	V	D	N	R	N	D	D	N	T	N	Y	F	I	W	P	G	R	T	P	E	L	R
147	RVA/Human-wt/ZAF/MRC-DPRU135/2009/G1P[8]	D	S	Q	D	S	T	N	L	N	G	I	T	A	D	P	V	D	N	R	N	D	D	N	T	N	Y	F	I	W	P	G	R	T	P	E	L	R
148	RVA/Human-wt/ZAF/MRC-DPRU1923/2009/G1P[8]	D	S	Q	D	S	T	N	L	N	G	I	T	A	D	P	V	D	N	R	N	D	D	N	T	N	Y	F	I	W	P	G	R	T	P	E	L	R
	South African post-vaccine strains																																					
149	RVA/Human-wt/RWA/UFS-NGS-MRC-DPRU2021/2010/G1P[8]	D	S	Q	D	S	T	N	L	N	G	I	T	A	D	P	V	D	N	R	N	D	D	N	T	N	Y	F	I	W	P	G	R	T	P	E	L	R
150	RVA/Human-wt/ZAF/UFS-NGS-MRC-DPRU2250/2013/G1P[8]	D	S	Q	E	S	T	N	L	N	N	I	T	A	N	P	V	D	S	S	N	D	N	N	T	N	Y	F	I	W	P	G	R	T	P	E	L	R
151	RVA/Human-wt/ZAF/UFS-NGS-MRC-DPRU56/2014/G1P[8]	D	S	Q	D	S	T	N	L	N	G	I	T	A	N	P	V	D	N	R	N	D	D	N	T	N	Y	F	I	W	P	G	R	T	P	E	L	R
152	RVA/Human-wt/ZAF/UFS-NGS-MRC-DPRU65/2014/G1P[8]	D	S	Q	D	S	T	N	L	N	G	I	T	A	N	P	V	D	N	R	N	D	D	N	T	N	Y	F	I	W	P	G	R	T	P	E	L	R
153	RVA/Human-wt/ZAF/UFS-NGS-MRC-DPRU74/2014/G1P[8]	D	S	Q	D	S	T	N	L	N	G	I	T	A	N	P	V	D	N	R	N	D	D	N	I	N	Y	F	I	W	P	G	R	T	P	E	L	R
154	RVA/Human-wt/ZAF/UFS-NGS-MRC-DPRU143/2014/G1P[8]	D	S	Q	D	S	T	N	L	N	G	I	T	A	N	P	V	D	N	R	N	D	D	N	T	N	Y	F	I	W	P	G	R	T	P	E	L	R
155	RVA/Human-wt/ZAF/UFS-NGS-MRC-DPRU150/2014/G1P[8]	D	S	Q	D	S	T	N	L	N	G	I	T	A	N	P	V	D	N	R	N	D	D	N	T	N	Y	F	I	W	P	G	R	T	P	E	L	R
156	RVA/Human-wt/ZAF/UFS-NGS-MRC-DPRU4347/2015/G1P[8]	D	S	Q	D	S	T	N	L	N	G	I	T	A	N	P	V	D	N	R	N	D	D	N	T	N	Y	F	I	W	P	G	R	T	P	E	L	R
157	RVA/Human-wt/ZAF/UFS-NGS-MRC-DPRU4352/2015/G1P[8]	D	S	Q	D	S	T	N	L	N	G	I	T	A	N	P	V	D	N	R	N	D	D	N	T	N	Y	F	I	W	P	G	R	T	P	E	L	R
158	RVA/Human-wt/ZAF/UFS-NGS-MRC-DPRU4357/2015/G1P[8]	D	S	Q	D	S	T	N	L	N	G	I	T	A	N	P	V	D	N	R	N	D	D	N	T	N	Y	F	I	W	P	G	R	T	P	E	L	R
159	RVA/Human-wt/ZAF/UFS-NGS-MRC-DPRU15948/2017/G1P[8]	D	S	Q	D	S	T	N	L	N	D	I	T	A	D	P	V	D	N	R	N	D	D	N	T	N	Y	F	I	W	P	G	R	T	P	E	L	R
160	RVA/Human-wt/ZAF/MRC-DPRU2061/2010/G1P[8]	D	S	Q	D	S	T	N	L	N	G	I	T	A	D	P	V	D	N	R	N	D	D	N	T	N	Y	F	I	W	P	G	R	T	P	E	L	R
161	RVA/Human-wt/ZAF/MRC-DPRU2035/2010/G1P[8]	D	S	Q	D	S	T	N	L	N	G	I	T	A	D	P	V	D	N	R	N	D	D	N	T	N	Y	F	I	W	P	G	R	T	P	E	L	R
162	RVA/Human-wt/ZAF/MRC-DPRU2030/2010/G1P[8]	D	S	Q	D	S	T	N	L	N	G	I	T	A	D	P	V	D	N	R	N	D	D	N	T	N	Y	F	I	W	P	G	R	T	P	E	L	R
163	RVA/Human-wt/ZAF/MRC-DPRU1544/2010/G1P[8]	D	S	Q	D	S	T	N	L	N	G	I	T	A	D	P	V	D	N	R	N	D	D	N	T	N	Y	F	I	W	P	G	R	T	P	E	L	R
164	RVA/Human-wt/ZAF/MRC-DPRU2052/2010/G1P[8]	D	S	Q	D	S	T	N	L	N	G	I	T	A	D	P	V	D	N	R	N	D	D	N	T	N	Y	F	I	W	P	G	R	T	P	E	L	R
165	RVA/Human-wt/ZAF/MRC-DPRU1492/2010/G1P[8]	D	S	Q	D	S	T	N	L	N	G	I	T	A	D	P	V	D	N	R	N	D	D	N	T	N	Y	F	I	W	P	G	R	T	P	E	L	R
166	RVA/Human-wt/ZAF/MRC-DPRU1506/2010/G1P[8]	D	S	Q	D	S	T	N	L	N	G	I	T	A	D	P	V	D	N	R	N	D	D	N	T	N	Y	F	I	W	P	G	R	T	P	E	L	R
167	RVA/Human-wt/ZAF/MRC-DPRU83/2011/G1P[8]	D	S	Q	D	S	T	N	L	N	D	I	T	A	D	P	V	D	N	R	N	D	D	N	T	S	Y	F	I	W	P	G	R	T	P	E	L	R
168	RVA/Human-wt/ZAF/MRC-DPRU6954/2011/G1P[8]	D	S	Q	D	S	T	N	L	N	G	I	T	A	D	P	V	D	N	R	N	D	D	N	T	N	Y	F	I	W	P	G	R	T	P	E	L	R
169	RVA/Human-wt/ZAF/MRC-DPRU120/2011/G1P[8]	D	S	Q	D	S	T	N	L	N	G	I	T	A	D	P	V	D	N	R	N	D	D	N	T	N	Y	F	I	W	P	G	R	T	P	E	L	R
170	RVA/Human-wt/ZAF/MRC-DPRU4079/2011/G1P[8]	D	S	Q	D	S	T	N	L	N	G	I	T	A	D	P	V	D	N	R	N	D	D	N	T	N	Y	F	I	W	P	G	R	T	P	E	L	R
171	RVA/Human-wt/ZAF/MRC-DPRU121/2011/G1P[8]	D	S	Q	D	S	T	N	L	N	G	I	T	A	D	P	V	D	N	R	N	D	D	N	T	N	Y	F	I	W	P	G	R	T	P	E	L	R

Figure S2: Alignment of antigenic residues in VP4 between the P[8] component of Rotarix® and wild type P[8] strains. Antigenic residues are divided into four antigenic epitopes in VP8* (8-1, 8-2, 8-3 and 8-4) and five antigenic epitopes in VP5* (5-1, 5-2, 5-3, 5-4 and 5-5). Amino acid changes that have been shown to escape neutralization with monoclonal antibodies are indicated with a black dot. Amino acids residues colored in sky blue differ from Rotarix®.
