# Supplementary material for: Whole Genome In-Silico Analysis of South African G1P[8] Rotavirus Strains before and after Vaccine Introduction over a Period of 14 Years
Source: Vaccines (Basel). 2020 Oct 14;8(4):609. doi: 10.3390/vaccines8040609 (PMC7712154; doi:10.3390/vaccines8040609)
Supplement: Supplementary file 1 [file vaccines-08-00609-s001.zip › supplementary--R1--AE/Supplementary data 6_Additional tables.pdf]

Supplementary data 6: Additional tables

Table S1: Amino acid differences in the VP7 neutralization and cytotoxic T-cell lymphocyte (CTL) epitope regions between Rotarix® and South African G1 strains

Amino acid difference occurrence period	Amino acid difference and number of strains	Region in the gene segment
**Both pre-vaccine and post-vaccine strains**	T91N (2), N94S (85), S123N (88), K291R (87)	7-1a epitope
	M217T (88)	7-2 epitope
	I22M (2), R28Q (1), A46V (3), T48A (20),T48I (4)	Cytotoxic T-cell lymphocyte epitope
**Only in pre-vaccine strains**	V212I (2)	7-1b epitope
	Q146R (2), S190L (1)	7-2 epitope
**Only in post-vaccine strains**	N147D (8)	7-2 epitope
	T242A (1)	7-1b epitope
	A43V(2), A46T (4)	Cytotoxic T-cell lymphocyte epitope

The table indicates amino acid differences in the VP7 neutralization and CTL epitope regions that were identified when South African G1 strains were compared with cognate regions in the Rotarix® VP7 strain. The number of strain(s) bearing the amino acid difference is indicated in brackets.

Table S2: Amino acid differences in the VP4 neutralization epitope regions between Rotarix® and South African P[4] strains

Amino acid difference occurrence period	Amino acid difference and number of strains	Region in the gene segment
**Both pre- vaccine and post vaccine strains**	E150D (97), N195D (3), N195G (162), N195S (5), N113D (102)	8-1 epitope
	S125N (165), S131R (170)	8-1 epitope
**Only in pre-vaccine strains**	N113 (3), S146N (1), S146G (13), N192D (5) N194I (4)	8-1 epitope
	I388L (4)	5-1 epitope
	E459D (5)	5-3 epitope
**Only in post-vaccine strains**	T88I (1), N89S (1)	8-4 epitope

The table indicates amino acid differences in the VP4 neutralization regions that were identified when South African P[8] strains were compared with cognate regions in the Rotarix® P[8]. The number of strain(s) bearing the amino acid difference is indicated in brackets.

Table S3: Amino acid differences in South African post-vaccine VP7 Cytotoxic T Lymphocyte epitope and non-epitope regions

No of strain(s)	Amino acid difference	Region	Amino acid property change	Possible effect
**All 2010 strains**	S37I	Signal domain (H2 hydrophobic domain)	Hydrophilic to hydrophobic; Neutral charge to neutral charge	The change in polarity charge may alter the chemical properties of the protein
**Four 2011 strains**	A46T	Signal domain (H2 hydrophobic domain)/ CTL epitope region	Hydrophobic to hydrophilic Neutral charge to neutral charge	The change in polarity may alter the chemical properties of the protein
**One 2011 strain**	E222K	β-jelly roll domain/ antigenic site C	Hydrophilic to hydrophilic Negative charge to positive charge	The change in charge may alter the chemical properties of the protein. The folding energy is significant for stabilizing effect on the protein structure
**One 2014 strain**	A68D	Extension region of N-terminal	Hydrophobic to hydrophilic Neutral charge to negative charge	The change in charge may alter the physicochemical properties of the protein
**Two 2015 strains**	A43V	Signal domain (H2 hydrophobic domain)/CTL epitope region	Hydrophobic to hydrophobic; Neutral charge to Neutral charge	The biochemical property of the region may be conserved as the amino acid property is not altered.

The table indicates the amino acid differences that were observed in the VP7 cytotoxic T lymphocyte epitopes at positions 43 (in two post-vaccine G1 strains) and 46 (in four post-vaccine G1 strains) during post-vaccine period. Also the figure shows the amino acid differences observed in non-neutralizing regions of South African post-vaccine G1 strains that were not present during pre-vaccine period.

Table S4: Amino acid differences in South African post-vaccine P[8] RVA strains

No of strain(s)	Amino acid difference(s)	Region	Amino acid property change	Possible effect
**All 2010 eight strains**	V112I	VP8*	Hydrophobic to hydrophobic; Neutral to neutral charge	No change in the physicochemical properties
**One 2015 strain**	V444A	VP5*	Hydrophobic to hydrophobic Neutral charge to neutral charge	No change in the physicochemical properties
**Two strains (one in 2010 and 2017)**	T498I	VP5*	Hydrophilic to hydrophobic Neutral charge to neutral charge	Possible change in the chemical properties
**All the 2014 and 2015 strains**	V600L	VP5*	Hydrophobic to hydrophobic Neutral charge to neutral charge	No change in the physicochemical properties
	K616R		Hydrophilic to hydrophilic Positive charge to positive charge	No change in the physicochemical properties

The table indicates the amino acid differences that were observed in the VP4 non-neutralization epitope sites at positions 43 (in two post-vaccine G1 strains) and 46 (in four post-vaccine G1 strains) during post-vaccine period. The asterisk indicates that VP5* and VP8* are proteolytic cleavage fragments of the VP4 protein.

Table S5: Amino acid differences in South African post-vaccine I1 RVA strains

Strain	Amino acid difference(s)	Region	Amino acid property change	Possible effect
**Strain RVA/Human-wt/ZAF/UFS-NGS-MRC-DPRU15948/2017/G1P[8]**	Y353H	Non-epitope	Hydrophilic to hydrophilic; Neutral charge to positive (10%)/neutral charge (90%)	The change in charge and destabilization effect might alter physicochemical properties of the protein

The table indicates the only amino acid difference that was observed in one post-vaccine I1 strain when comparison of South African pre and post-vaccine strains was performed.

Table S6: Amino acid differences in South African post-vaccine R1 RVA strains

No of strains(s)	Amino acid difference(s)	Region	Amino acid property change	Possible effect
**All the 2010 strains**	L167M	N-terminal domain	Hydrophobic to hydrophobic; Neutral charge to neutral charge	The polarity change may alter biochemical properties of the protein
	T352N	Polymerase domain (Finger subdomain)	Hydrophilic to hydrophilic; Neutral charge to Neutral charge	
	A1028S	C-terminal domain	Hydrophobic to hydrophilic Neutral charge to neutral charge	
**Four of the 2011 strains**	K164R	N-terminal domain	Hydrophilic to hydrophilic Positive charge to positive charge	The change in charge may alter biochemical properties of the protein
	I279V,V301A	N-terminal domain	Hydrophobic to hydrophobic Neutral charge to neutral charge	
	K898T	C-terminal domain	Hydrophilic to hydrophilic Positive charge to neutral charge	
**All the 2014 and 2015 strains**	K96R	N-terminal domain	Hydrophilic to hydrophobic Positive charge to positive charge	The change in charge and polarity may alter biochemical properties of the protein
	K164R			
	D286N		Hydrophilic to hydrophilic Negative charge to neutral charge	
**Strain RVA/Human-wt/ZAF/MRC-DPRU15948/2017/G1P[8]**	I231T	N-terminal domain	Hydrophobic to hydrophilic Neutral charge to neutral charge	The polarity change may alter biochemical properties of the protein
	P969L	C-terminal domain	Hydrophobic to hydrophobic Neutral charge to neutral charge	

The table indicates the amino acid difference that were observed during post-vaccine period in different South African VP1 RVA when compared to cognate regions in pre-vaccine strains.

Table S7: Amino acid differences in South African post-vaccine C1 RVA strains

No of strain (s)	Amino acid difference(s)	Region	Amino acid property change	Possible effect
**All the 2010 strains**	R44K	Nucleic acid binding domain	Hydrophilic to hydrophilic; Positive charge to positive charge	No effect on the physicochemical properties of the protein
**Four of the 2011 strains**	N30E	Nucleic acid binding domain	Hydrophilic to hydrophilic Neutral charge to negative charge	The mutations may not alter the biochemical properties of the protein
	T32N		Hydrophilic to hydrophilic Neutral charge to neutral charge	
**Strain RVA/Human-wt/ZAF/MRC-DPRU83/2011/G1P[8]**	P14L	Nucleic acid binding domain	Hydrophobic to hydrophobic Neutral charge to neutral charge	The mutations may not alter the biochemical properties of the protein
	N510S	Principal domain	Hydrophilic to hydrophilic Neutral to neutral	
**Two 2015 strains**	P669S	Principal domain	Hydrophobic to hydrophilic Neutral charge to neutral charge	The change in polarity may alter the biochemical properties of the protein

The table indicates the amino acid differences that were observed during the post-vaccine period in different South African VP2 RVA when compared to cognate regions in pre-vaccine strains.

Table S8: Amino acid differences in South African post-vaccine M1 RVA strains

No of strain(s)	Mutation(s)	Region(s)	Amino acid property change	Possible effect
**All the 2010 strains**	I234V	N7-Methyltransferase	Hydrophobic to hydrophobic; Neutral charge to neutral charge	The mutation may not alter the biochemical properties of the protein
**Four of the 2011 strains**	A411V and M762	2'-O—Methyltransferase domain and phosphodiesterase domains, respectively	Hydrophobic to hydrophobic Neutral charge to neutral charge	The mutation may not alter the biochemical properties of the protein
**All the 2014 and 2015 strains**	V459I and M762	N7-Methyltransferase and phosphodiesterase domains, respectively	Hydrophobic to hydrophobic Neutral charge to neutral charge	The mutation may not alter the biochemical properties of the protein
**Strain RVA/Human-wt/ZAF/MRC-DPRU15948/2017/G1P[8]**	K204R	N7-Methyltransferase domain	Hydrophilic to hydrophilic Positive charge to positive charge	The mutation may potentially not alter the biochemical properties of the protein
	N804S	Phosphodiesterase domain	Hydrophilic to hydrophilic Neutral charge to neutral charge	

The table indicates the amino acid differences that were observed during the post-vaccine period in different South African VP3 RVA when compared to cognate regions in pre-vaccine strains.

Table S9: Amino acid differences in South African post-vaccine A1 RVA strains

No. of strain(s)	Amino acid difference(s)	Region	Amino acid property change	Possible effect
**All the 2010 strains**	T120I	Cytoskeleton binding domain	Hydrophilic to hydrophobic; Neutral charge to neutral charge	The change in polarity may alter the biochemical properties
	V266I	Undefined region	Hydrophobic to hydrophobic; Neutral charge to Neutral charge	
**Four of the 2011 strains**	V189I,V266I, I278V	Undefined region	Hydrophobic to hydrophobic Neutral charge to neutral charge	The mutation may not alter the biochemical properties
	I341V	IRF-binding domain		
	D381Y	IRF-binding domain	Hydrophilic to hydrophilic Negative charge to neutral charge	
	Y445H	IRF-binding domain	Hydrophilic to hydrophilic Neutral to positive (10%)/ neutral (90%)	
**One 2013 strain**	Q132R	RING domain	Hydrophilic to hydrophilic Neutral charge to positive charge	The change in charge may alter the biochemical properties
**All 2014 strain and one 2015 strain RVA/Human-wt/ZAF/MRC-DPRU4357/2015/G1P[8]**	R13K	RING domain	Hydrophobic to hydrophilic Positive charge to positive charge	The change in polarity may alter the biochemical properties
	A259T /A298V	Undefined region	Hydrophobic to hydrophilic Neutral charge to neutral charge	

The table indicates the amino acid differences that were observed during the post-vaccine period in different South African NSP1 RVA when compared to cognate regions in pre-vaccine strains.

Table S10: Amino acid differences in South African post-vaccine N1 RVA strains

No of strain(s)	Amino acid difference(s)	Region	Amino acid property change	Possible effect
**2010 strain RVA/Human-wt/ZAF/MRC-DPRU2061/2010/G1P[8]**	E73K	N terminal domain	Hydrophilic to hydrophilic; Negative charge to positive charge	The change in charge may alter the biochemical properties
**Four 2011 strains**	V254P	C terminal domain	Hydrophobic to hydrophobic Neutral charge to neutral charge	The mutation may not alter the biochemical properties
**2014 Strain RVA/Human-wt/ZAF/MRC-DPRU143/2014/G1P[8]**	P299S	C terminal domain	Hydrophobic to hydrophilic Neutral charge to neutral charge	The change in charge may alter the functionality of the protein

The table indicates the amino acid differences that were observed during the post-vaccine period in different South African NSP2 RVA when compared to cognate regions in pre-vaccine strains.

Table S11: Amino acid differences in South African post-vaccine T1 RVA strains

No of strain(s)	Amino acid difference (s)	Region	Amino acid property change	Possible effect
**Two 2015 strains**	S169F	Dimerization domain	Hydrophilic to hydrophobic; Neutral charge to neutral charge	The change in polarity may alter the biochemical properties
	N215S	C-terminal domain	Hydrophilic to hydrophilic; Neutral charge to neutral charge	
**2014 strain RVA/Human-wt/ZAF/MRC-DPRU65/2014/G1P[8**	Y204F	Dimerization domain	Hydrophilic to hydrophobic; Neutral charge to neutral charge	The change in polarity may alter the biochemical properties
	K256R	C-terminal domain	Hydrophilic to hydrophilic Positive charge to positive charge	

The table indicates the amino acid differences that were observed during the post-vaccine period in different South African NSP3 RVA when compared to cognate regions in pre-vaccine strains.

Table S12: Amino acid differences in South African post-vaccine E1 RVA strains

No of strain(s)	Amino acid difference (s)	Region	Amino acid property change	Possible effect
**All 2010 strains**	V73I	N terminal hydrophobic domain 3 (H3)	Hydrophobic to hydrophobic; Neutral charge to neutral charge	The mutation may not alter the biochemical properties
**Strain RVA/Human-wt/ZAF/MRC-DPRU83/2011/G1P[8]**	D157E	Tubulin binding domain/AS-I	Hydrophilic to hydrophilic; Negative charge to negative charge	The mutation may not alter the biochemical properties
**Strain RVA/Human-wt/ZAF/MRC-DPRU15948/2017/G1P[8]**	V13I	N-terminal hydrophobic domain 1(H1)/AS-IV	Hydrophobic to hydrophobic; Neutral charge to neutral charge	The mutation may not alter the biochemical properties
	D157E	Tubulin binding domain/AS-I	Hydrophilic to hydrophilic; Negative charge to negative charge	The mutation may not alter the biochemical properties
	E167G	DLP-binding region/AS-I	Hydrophilic to hydrophobic Negative to neutral charge	The change in polarity may alter the biochemical properties

The table indicates the amino acid differences that were observed during the post-vaccine period in different South African NSP3 RVA when compared to cognate regions in pre-vaccine strains.

Table S13: Amino acid differences in South African post-vaccine H1 RVA strains

No of strain(s)	Amino acid difference(s)	Region	Amino acid property change	Possible effect
**Four 2010 strains**	S22L	N-terminal binding domain	Hydrophilic to hydrophobic; Neutral charge to neutral charge	The change in polarity may alter the biochemical properties
**Four 2011 strains**	D157E	C-terminal binding domain	Hydrophilic to hydrophilic; Positive charge to negative charge	The change in charge may alter the biochemical properties

The table indicates the amino acid differences that were observed during the post-vaccine period in different South African NSP3 RVA when compared to cognate regions in pre-vaccine strains.
