# Supplementary material for: Whole Genome In-Silico Analysis of South African G1P[8] Rotavirus Strains before and after Vaccine Introduction over a Period of 14 Years
Source: Vaccines (Basel). 2020 Oct 14;8(4):609. doi: 10.3390/vaccines8040609 (PMC7712154; doi:10.3390/vaccines8040609)
Supplement: Supplementary file 1 [file vaccines-08-00609-s001.zip › supplementary--R1--AE/Supplementary data 7_Additional protein modeling analysis.pdf]

Supplementary data 7: Additional protein modeling analysis

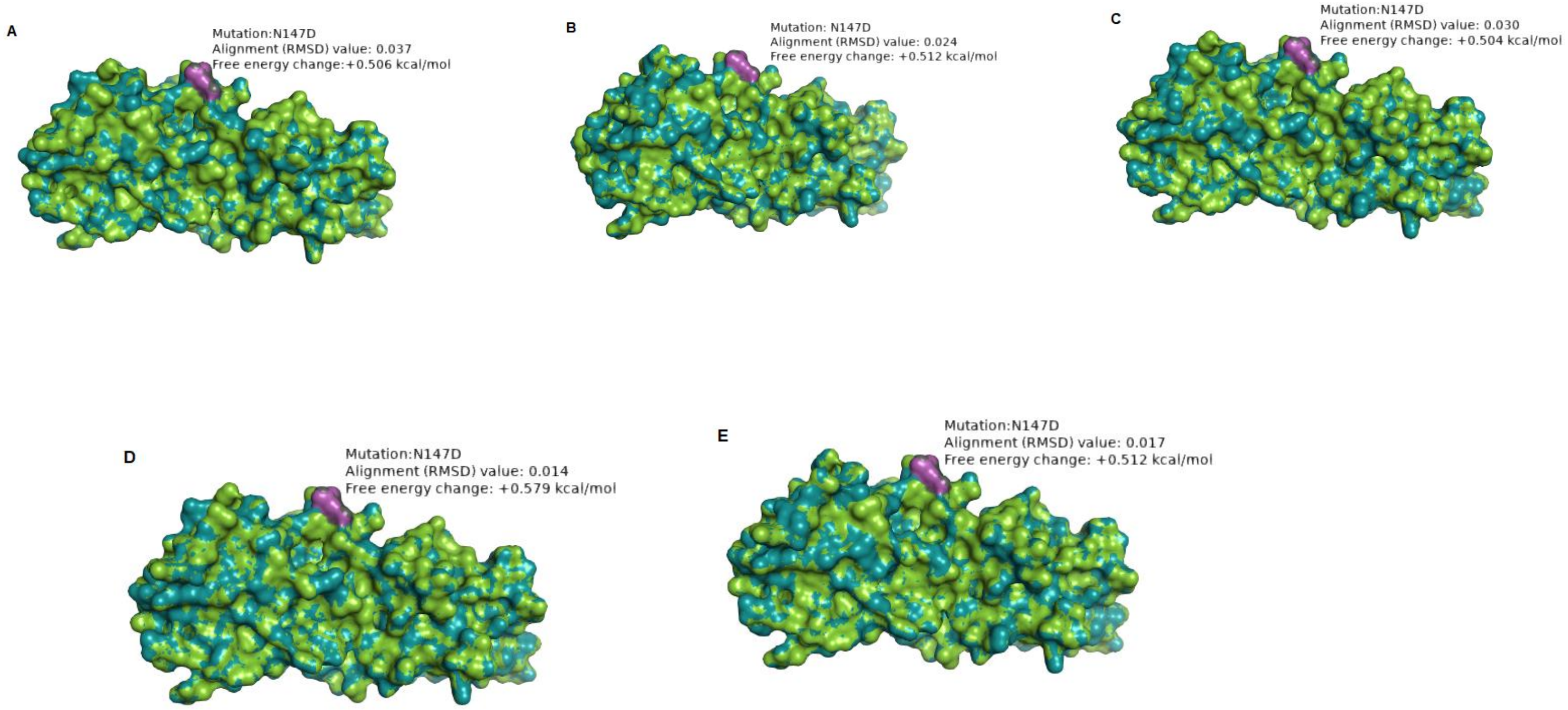

Figure S1: The VP7 structure models showing the N147D amino acid difference. The structure of the post-vaccine study strain, RVA/Human-wt/ZAF/UFS-NGS-MRC-DPRU4357/2015/G1P[8, is in green while for pre-vaccine study strains is in deep-teal. Five pre-vaccine strains were randomly selected for the protein modeling analysis. The amino acid residues are represented in dark gray and magenta to examine whether the replacing amino acid alters the conformation of the protein structure. The amino acid residue highlighted in dark gray represents the post-vaccine strain RVA/Human-wt/ZAF/UFS-NGS-MRC-DPRU4357/2015/G1P[8] while magenta represents the pre-vaccine strains . Relative Mean Square Deviation (RMSD) is the superimposition value where value of zero indicates absolute similarity. The stability of the protein after mutation was measured in kcal/mol whereby folding energy change of ±0.5 kcal/mol is regarded as statistically significant for either stabilizing (-)/destabilizing (+) effect.

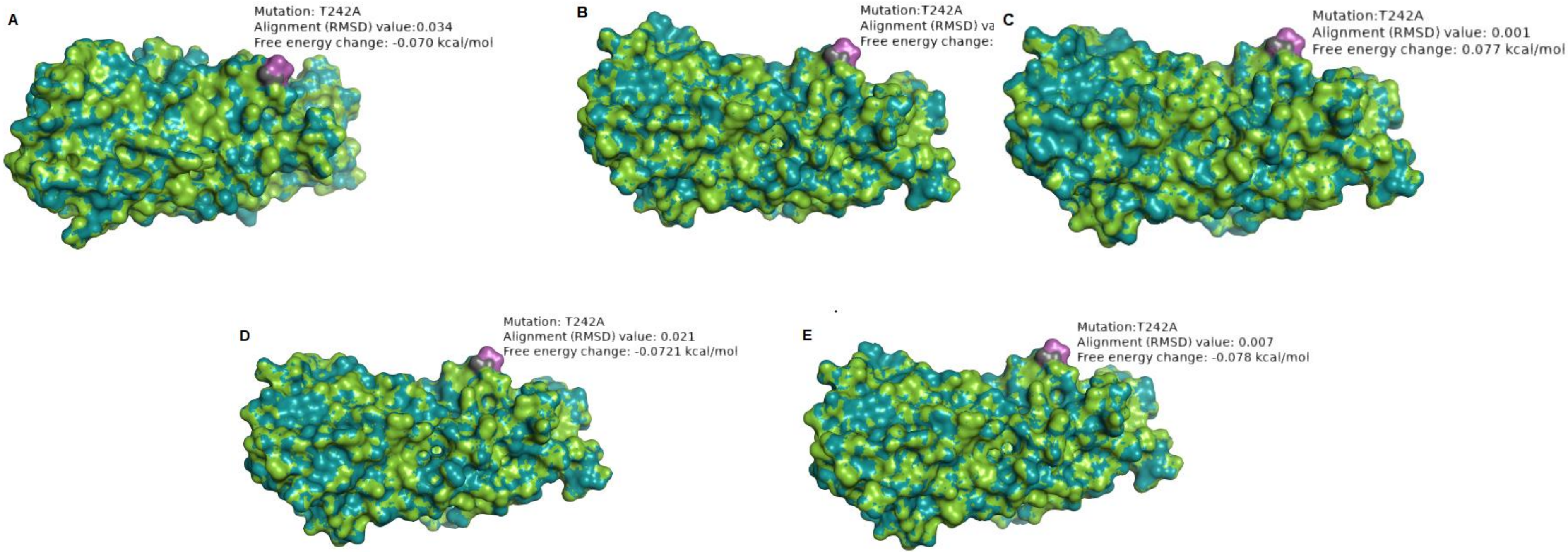

Figure S2: The VP7 structure models showing the T242A amino acid difference. The structure of the post-vaccine study strain is in green while for pre-vaccine study strains is in deep-teal. Five pre-vaccine strains were randomly selected for the protein modeling analysis. The amino acid residues are represented in dark gray and magenta to examine whether the replacing amino acid alters the conformation of the protein structure. The amino acid residue highlighted in dark gray represents the post-vaccine strain RVA/Human-wt/ZAF/MRC-DPRU1544/2010/G1P[8] while magenta represents the pre-vaccine strains . Relative Mean Square Deviation (RMSD) is the superimposition value where value of zero indicates absolute similarity. The stability of the protein after mutation was measured in kcal/mol whereby folding energy change of ±0.5 kcal/mol is regarded as statistically significant for either stabilizing (-)/destabilizing (+) effect.

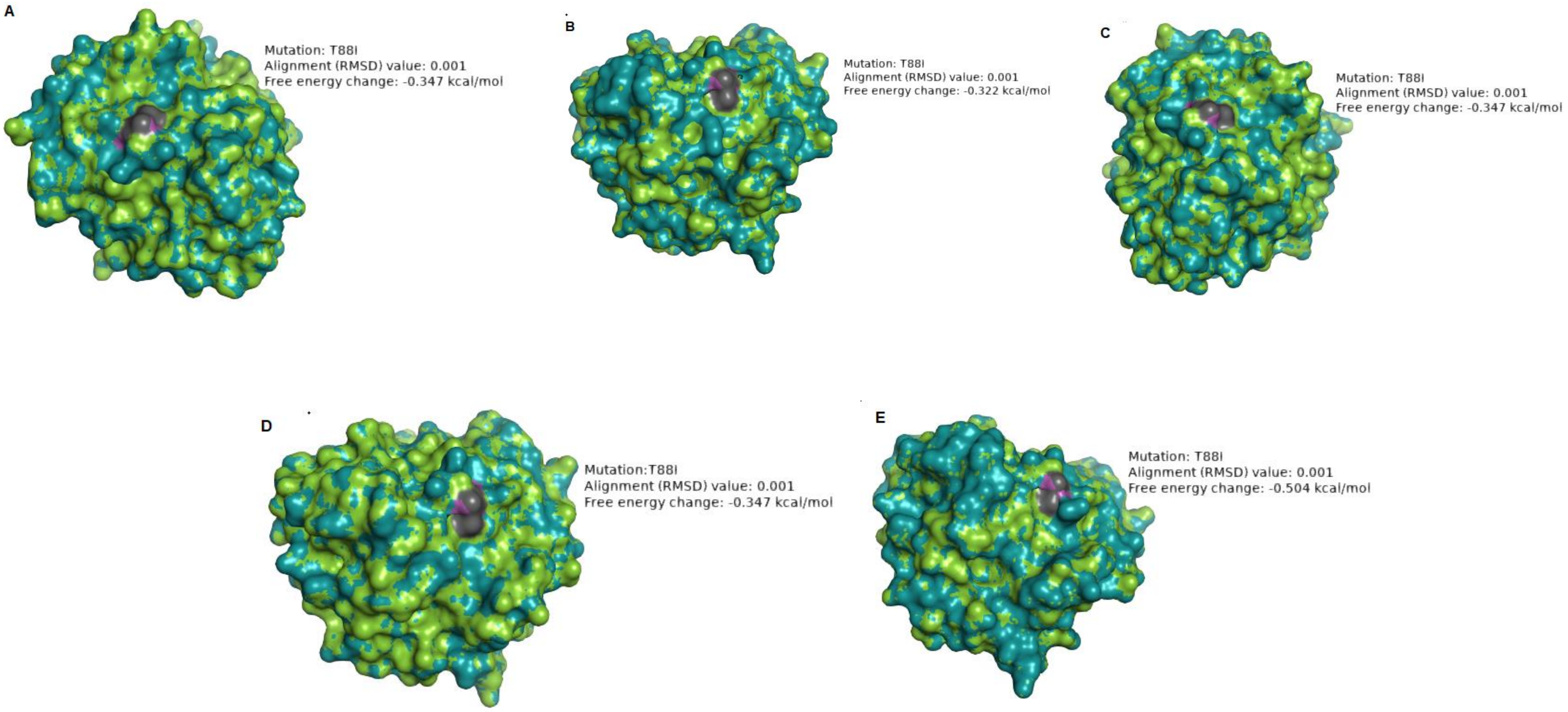

Figure S3: The VP4 structure models showing the T88I amino acid difference. The structure of the post-vaccine study strain RVA/Human-wt/ZAF/UFS-NGS-MRC-DPRU74/2014/G1P[8] is in green while for pre-vaccine study strains is in deep-teal. Five pre-vaccine strains were randomly selected for the protein modeling analysis. The amino acid residues are represented in dark gray and magenta to examine whether the replacing amino acid alters the conformation of the protein structure. The amino acid residue highlighted in dark gray represents the post-vaccine strain RVA/Human-wt/ZAF/UFS-NGS-MRC-DPRU74/2014/G1P[8] while magenta represents the pre-vaccine strains. Relative Mean Square Deviation (RMSD) is the superimposition value where value of zero indicates absolute similarity. The stability of the protein after mutation was measured in kcal/mol whereby folding energy change of ±0.5 kcal/mol is regarded as statistically significant for either stabilizing (-)/destabilizing (+) effect

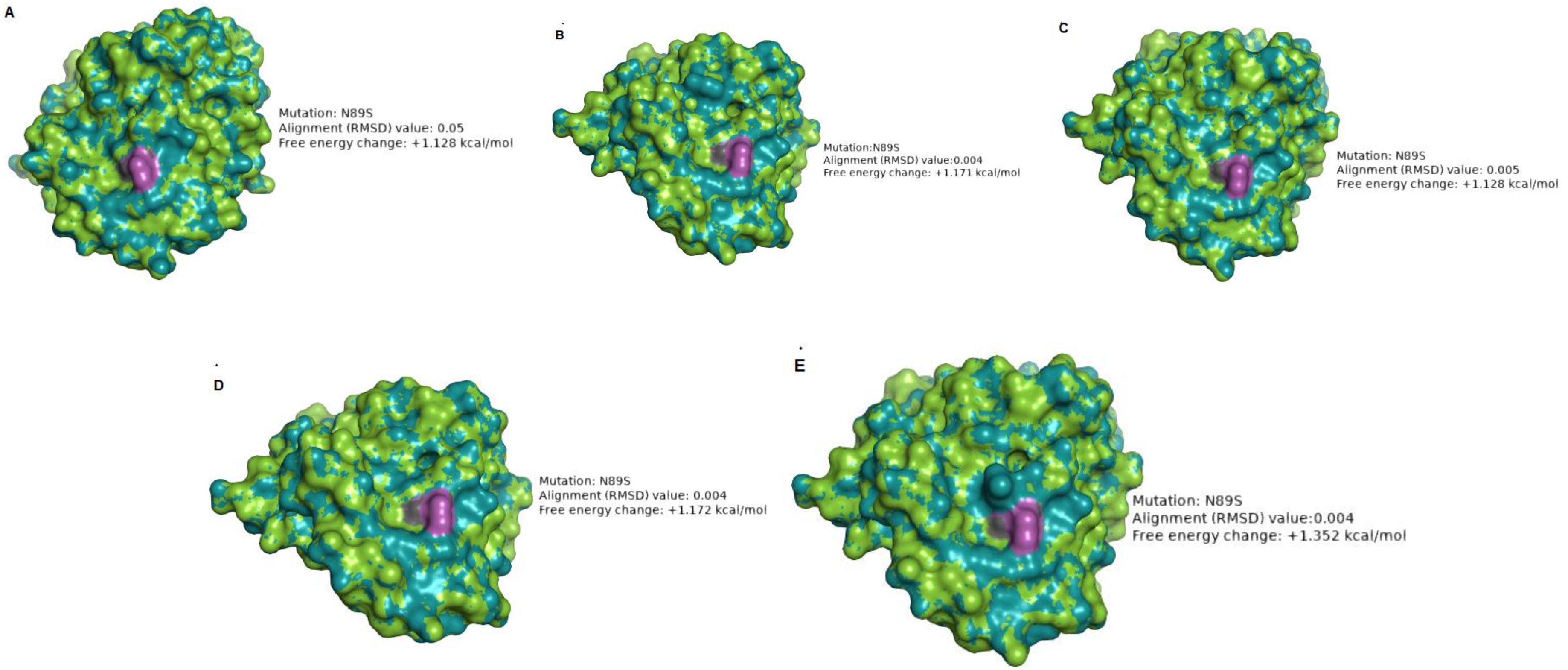

Figure S4: The VP4 structure models showing the N89S amino acid difference. The structure of the post-vaccine study strain RVA/Human-wt/ZAF/UFS-NGS-MRC-DPRU83/2011/G1P[8] is in green while for pre-vaccine study strains is in deep-teal. Five pre-vaccine strains were randomly selected for the protein modeling analysis. The amino acid residues are represented in dark gray and magenta to examine whether the replacing amino acid alters the conformation of the protein structure. The amino acid residue highlighted in dark gray represents the post-vaccine strain RVA/Human-wt/ZAF/UFS-NGS-MRC-DPRU83/2011/G1P[8] while magenta represents the pre-vaccine strains. Relative Mean Square Deviation (RMSD) is the superimposition value where value of zero indicates absolute similarity. The stability of the protein after mutation was measured in kcal/mol whereby folding energy change of ±0.5 kcal/mol is regarded as statistically significant for either stabilizing (-)/destabilizing (+) effect.
