# Supplementary material for: Whole Genome In-Silico Analysis of South African G1P[8] Rotavirus Strains before and after Vaccine Introduction over a Period of 14 Years
Source: Vaccines (Basel). 2020 Oct 14;8(4):609. doi: 10.3390/vaccines8040609 (PMC7712154; doi:10.3390/vaccines8040609)
Supplement: Supplementary file 1 [file vaccines-08-00609-s001.zip › supplementary--R1--AE/supplementary-S1--XML.pdf]

Table S1. Whole genotype constellations of 103 sequenced South African G1P[8] rotavirus strains and 68 South African G1P[8] strains acquired from GenBank

	Gene segment	VP7	VP7 Lineage	VP4	VP4 Lineage	VP6	VP1	VP2	VP3	NSP1	NSP2	NSP3	NSP4	NSP5	Accession codes in GenBank (MT854335-MT855467)
	**Base pair size for full length sequences**	1062		2359		1356	3302	2717	2591	1567	1059	1074	750	664	
	**Base pair size for open reading frame (ORF) sequences**	978		2325		1191	3264	2682	2505	1458	951	930	525	591	
	**Genotype constellations**														
	**Sequenced for this study**														
	**Pre-vaccine strains**														
1	RVA/Human-wt/ZAF/UFS-NGS-MRC-DPRU3883/2002/G1P[8]	G1	II	P[8]	III	I1	R1	C1	M1	A1	N1	T1	E1	H1	
2	RVA/Human-wt/ZAF/UFS-NGS-MRC-DPRU3892/2002/G1P[8]	G1	II	P[8]	III	I1	R1	C1	M1	A1	N1	T1	E1	H1	
3	RVA/Human-wt/ZAF/UFS-NGS-MRC-DPRU3951/2002/G1P[8]	G1	II	P[8]	III	I1	R1	C1	M1	A1	N1	T1	E1	H1	
4	RVA/Human-wt/ZAF/UFS-NGS-MRC-DPRU3956/2002/G1P[8]	G1	II	P[8]	III	I1	R1	C1	M1	A1	N1	T1	E1	H1	
5	RVA/Human-wt/ZAF/UFS-NGS-MRC-DPRU4018/2002/G1P[8]	G1	II	P[8]	III	I1	R1	C1	M1	A1	N1	T1	E1	H1	
6	RVA/Human-wt/ZAF/UFS-NGS-MRC-DPRU4019/2002/G1P[8]	G1	II	P[8]	III	I1	R1	C1	M1	A1	N1	T1	E1	H1	
7	RVA/Human-wt/ZAF/UFS-NGS-MRC-DPRU4023/2002/G1P[8]	G1	II	P[8]	III	I1	R1	C1	M1	A1	N1	T1	E1	H1	
8	RVA/Human-wt/ZAF/UFS-NGS-MRC-DPRU4024/2002/G1P[8]	G1	I	P[8]	III	I1	R1	C1	M1	A1	N1	T1	E1	H1	
9	RVA/Human-wt/ZAF/UFS-NGS-MRC-DPRU4026/2002/G1P[8]	G1	II	P[8]	III	I1	R1	C1	M1	A1	N1	T1	E1	H1	
10	RVA/Human-wt/ZAF/UFS-NGS-MRC-DPRU4049/2002/G1P[8]	G1	II	P[8]	III	I1	R1	C1	M1	A1	N1	T1	E1	H1	
11	RVA/Human-wt/ZAF/UFS-NGS-MRC-DPRU4055/2002/G1P[8]	G1	II	P[8]	III	I1	R1	C1	M1	A1	N1	T1	E1	H1	
12	RVA/Human-wt/ZAF/UFS-NGS-MRC-DPRU4102/2002/G1P[8]	G1	II	P[8]	III	I1	R1	C1	M1	A1	N1	T1	E1	H1	
13	RVA/Human-wt/ZAF/UFS-NGS-MRC-DPRU4269/2002/G1P[8]	G1	II	P[8]	III	I1	R1	C1	M1	A1	N1	T1	E1	H1	
14	RVA/Human-wt/ZAF/UFS-NGS-MRC-DPRU4271/2002/G1P[8]	G1	II	P[8]	III	I1	R1	C1	M1	A1	N1	T1	E1	H1	
15	RVA/Human-wt/ZAF/UFS-NGS-MRC-DPRU456/2003/G1P[8]	G1	II	P[8]	III	I1	R1	C1	M1	A1	N1	T1	E1	H1	
16	RVA/Human-wt/ZAF/UFS-NGS-MRC-DPRU493/2003/G1P[8]	G1	II	P[8]	III	I1	R1	C1	M1	A1	N1	T1	E1	H1	
17	RVA/Human-wt/ZAF/UFS-NGS-MRC-DPRU609/2003/G1P[8]	G1	II	P[8]	III	I1	R1	C1	M1	A1	N1	T1	E1	H1	
18	RVA/Human-wt/ZAF/UFS-NGS-MRC-DPRU632/2003/G1P[8]	G1	II	P[8]	III	I1	R1	C1	M1	A1	N1	T1	E1	H1	
19	RVA/Human-wt/ZAF/UFS-NGS-MRC-DPRU2117/2003/G1P[8]	G1	II	P[8]	III	I1	R1	C1	M1	A1	N1	T1	E1	H1	
20	RVA/Human-wt/ZAF/UFS-NGS-MRC-DPRU2145/2003/G1P[8]	G1	II	P[8]	III	I1	R1	C1	M1	A1	N1	T1	E1	H1	
21	RVA/Human-wt/ZAF/UFS-NGS-MRC-DPRU2156/2003/G1P[8]	G1	II	P[8]	III	I1	R1	C1	M1	A1	N1	T1	E1	H1	
22	RVA/Human-wt/ZAF/UFS-NGS-MRC-DPRU1104/2004/G1P[8]	G1	II	P[8]	III	I1	R1	C1	M1	A1	N1	T1	E1	H1	
23	RVA/Human-wt/ZAF/UFS-NGS-MRC-DPRU1116/2004/G1P[8]	G1	I	P[8]	III	I1	R1	C1	M1	A1	N1	T1	E1	H1	
24	RVA/Human-wt/ZAF/UFS-NGS-MRC-DPRU1131/2004/G1P[8]	G1	II	P[8]	III	I1	R1	C1	M1	A1	N1	T1	E1	H1	
25	RVA/Human-wt/ZAF/UFS-NGS-MRC-DPRU1135/2004/G1P[8]	G1	II	P[8]	III	I1	R1	C1	M1	A1	N1	T1	E1	H1	
26	RVA/Human-wt/ZAF/UFS-NGS-MRC-DPRU1139/2004/G1P[8]	G1	II	P[8]	III	I1	R1	C1	M1	A1	N1	T1	E1	H1	
27	RVA/Human-wt/ZAF/UFS-NGS-MRC-DPRU1145/2004/G1P[8]	G1	II	P[8]	III	I1	R1	C1	M1	A1	N1	T1	E1	H1	
28	RVA/Human-wt/ZAF/UFS-NGS-MRC-DPRU1148/2004/G1P[8]	G1	II	P[8]	III	I1	R1	C1	M1	A1	N1	T1	E1	H1	
29	RVA/Human-wt/ZAF/UFS-NGS-MRC-DPRU1184/2004/G1P[8]	G1	II	P[8]	III	I1	R1	C1	M1	A1	N1	T1	E1	H1	
30	RVA/Human-wt/ZAF/UFS-NGS-MRC-DPRU1192/2004/G1P[8]	G1	II	P[8]	III	I1	R1	C1	M1	A1	N1	T1	E1	H1	
31	RVA/Human-wt/ZAF/UFS-NGS-MRC-DPRU1205/2004/G1P[8]	G1	II	P[8]	III	I1	R1	C1	M1	A1	N1	T1	E1	H1	
32	RVA/Human-wt/ZAF/UFS-NGS-MRC-DPRU1230/2004/G1P[8]	G1	II	P[8]	III	I1	R1	C1	M1	A1	N1	T1	E1	H1	
33	RVA/Human-wt/ZAF/UFS-NGS-MRC-DPRU1287/2004/G1P[8]	G1	II	P[8]	III	I1	R1	C1	M1	A1	N1	T1	E1	H1	
34	RVA/Human-wt/ZAF/UFS-NGS-MRC-DPRU3410/2004/G1P[8]	G1	II	P[8]	III	I1	R1	C1	M1	A1	N1	T1	E1	H1	
35	RVA/Human-wt/ZAF/UFS-NGS-MRC-DPRU3449/2004/G1P[8]	G1	II	P[8]	III	I1	R1	C1	M1	A1	N1	T1	E1	H1	
36	RVA/Human-wt/ZAF/UFS-NGS-MRC-DPRU451/2005/G1P[8]	G1	II	P[8]	III	I1	R1	C1	M1	A1	N1	T1	E1	H1	
37	RVA/Human-wt/ZAF/UFS-NGS-MRC-DPRU658/2005/G1P[8]	G1	I	P[8]	III	I1	R1	C1	M1	A1	N1	T1	E1	H1	
38	RVA/Human-wt/ZAF/UFS-NGS-MRC-DPRU682/2005/G1P[8]	G1	II	P[8]	IV	I1	R1	C1	M1	A1	N1	T1	E1	H1	
39	RVA/Human-wt/ZAF/UFS-NGS-MRC-DPRU687/2005/G1P[8]	G1	II	P[8]	III	I1	R1	C1	M1	A1	N1	T1	E1	H1	
40	RVA/Human-wt/ZAF/UFS-NGS-MRC-DPRU745/2005/G1P[8]	G1	I	P[8]	III	I1	R1	C1	M1	A1	N1	T1	E1	H1	
41	RVA/Human-wt/ZAF/UFS-NGS-MRC-DPRU747/2005/G1P[8]	G1	II	P[8]	III	I1	R1	C1	M1	A1	N1	T1	E1	H1	
42	RVA/Human-wt/ZAF/UFS-NGS-MRC-DPRU732/2006/G1P[8]	G1	I	P[8]	III	I1	R1	C1	M1	A1	N1	T1	E1	H1	
43	RVA/Human-wt/ZAF/UFS-NGS-MRC-DPRU749/2006/G1P[8]	G1	I	P[8]	III	I1	R1	C1	M1	A1	N1	T1	E1	H1	
44	RVA/Human-wt/ZAF/UFS-NGS-MRC-DPRU753/2006/G1P[8]	G1	I	P[8]	III	I1	R1	C1	M1	A1	N1	T1	E1	H1	
45	RVA/Human-wt/ZAF/UFS-NGS-MRC-DPRU757/2006/G1P[8]	G1	I	P[8]	III	I1	R1	C1	M1	A1	N1	T1	E1	H1	
46	RVA/Human-wt/ZAF/UFS-NGS-MRC-DPRU768/2006/G1P[8]	G1	I	P[8]	III	I1	R1	C1	M1	A1	N1	T1	E1	H1	
47	RVA/Human-wt/ZAF/UFS-NGS-MRC-DPRU783/2006/G1P[8]	G1	I	P[8]	III	I1	R1	C1	M1	A1	N1	T1	E1	H1	
48	RVA/Human-wt/ZAF/UFS-NGS-MRC-DPRU849/2006/G1P[8]	G1	I	P[8]	III	I1	R1	C1	M1	A1	N1	T1	E1	H1	
49	RVA/Human-wt/ZAF/UFS-NGS-MRC-DPRU902/2006/G1P[8]	G1	I	P[8]	III	I1	R1	C1	M1	A1	N1	T1	E1	H1	
50	RVA/Human-wt/ZAF/UFS-NGS-MRC-DPRU981/2006/G1P[8]	G1	I	P[8]	III	I1	R1	C1	M1	A1	N1	T1	E1	H1	
51	RVA/Human-wt/ZAF/UFS-NGS-MRC-DPRU999/2006/G1P[8]	G1	I	P[8]	III	I1	R1	C1	M1	A1	N1	T1	E1	H1	
52	RVA/Human-wt/ZAF/UFS-NGS-MRC-DPRU1002/2006/G1P[8]	G1	I	P[8]	III	I1	R1	C1	M1	A1	N1	T1	E1	H1	
53	RVA/Human-wt/ZAF/UFS-NGS-MRC-DPRU1005/2006/G1P[8]	G1	I	P[8]	III	I1	R1	C1	M1	A1	N1	T1	E1	H1	
54	RVA/Human-wt/ZAF/UFS-NGS-MRC-DPRU1006/2006/G1P[8]	G1	II	P[8]	III	I1	R1	C1	M1	A1	N1	T1	E1	H1	
55	RVA/Human-wt/ZAF/UFS-NGS-MRC-DPRU1007/2006/G1P[8]	G1	I	P[8]	III	I1	R1	C1	M1	A1	N1	T1	E1	H1	
56	RVA/Human-wt/ZAF/UFS-NGS-MRC-DPRU1009/2006/G1P[8]	G1	II	P[8]	III	I1	R1	C1	M1	A1	N1	T1	E1	H1	
57	RVA/Human-wt/ZAF/UFS-NGS-MRC-DPRU1012/2006/G1P[8]	G1	I	P[8]	III	I1	R1	C1	M1	A1	N1	T1	E1	H1	
58	RVA/Human-wt/ZAF/UFS-NGS-MRC-DPRU1015/2006/G1P[8]	G1	I	P[8]	III	I1	R1	C1	M1	A1	N1	T1	E1	H1	
59	RVA/Human-wt/ZAF/UFS-NGS-MRC-DPRU1019/2006/G1P[8]	G1	II	P[8]	III	I1	R1	C1	M1	A1	N1	T1	E1	H1	
60	RVA/Human-wt/ZAF/UFS-NGS-MRC-DPRU1030/2006/G1P[8]	G1	I	P[8]	III	I1	R1	C1	M1	A1	N1	T1	E1	H1	
61	RVA/Human-wt/ZAF/UFS-NGS-MRC-DPRU1039/2006/G1P[8]	G1	I	P[8]	III	I1	R1	C1	M1	A1	N1	T1	E1	H1	
62	RVA/Human-wt/ZAF/UFS-NGS-MRC-DPRU1041/2006/G1P[8]	G1	I	P[8]	III	I1	R1	C1	M1	A1	N1	T1	E1	H1	
63	RVA/Human-wt/ZAF/UFS-NGS-MRC-DPRU3433/2006/G1P[8]	G1	I	P[8]	IV	I1	R1	C1	M1	A1	N1	T1	E1	H1	
64	RVA/Human-wt/ZAF/UFS-NGS-MRC-DPRU147/2007/G1P[8]	G1	I	P[8]	III	I1	R1	C1	M1	A1	N1	T1	E1	H1	
65	RVA/Human-wt/ZAF/UFS-NGS-MRC-DPRU792/2007/G1P[8]	G1	I	P[8]	III	I1	R1	C1	M1	A1	N1	T1	E1	H1	
66	RVA/Human-wt/ZAF/UFS-NGS-MRC-DPRU793/2007/G1P[8]	G1	I	P[8]	III	I1	R1	C1	M1	A1	N1	T1	E1	H1	
67	RVA/Human-wt/ZAF/UFS-NGS-MRC-DPRU799/2007/G1P[8]	G1	I	P[8]	III	I1	R1	C1	M1	A1	N1	T1	E1	H1	
68	RVA/Human-wt/ZAF/UFS-NGS-MRC-DPRU1273/2007/G1P[8]	G1	I	P[8]	III	I1	R1	C1	M1	A1	N1	T1	E1	H1	
69	RVA/Human-wt/ZAF/UFS-NGS-MRC-DPRU1302/2007/G1P[8]	G1	II	P[8]	III	I1	R1	C1	M1	A1	N1	T1	E1	H1	
70	RVA/Human-wt/ZAF/UFS-NGS-MRC-DPRU1652/2007/G1P[8]	G1	II	P[8]	III	I1	R1	C1	M1	A1	N1	T1	E1	H1	
71	RVA/Human-wt/ZAF/UFS-NGS-MRC-DPRU1042/2008/G1P[8]	G1	II	P[8]	III	I1	R1	C1	M1	A1	N1	T1	E1	H1	
72	RVA/Human-wt/ZAF/UFS-NGS-MRC-DPRU1047/2008/G1P[8]	G1	I	P[8]	III	I1	R1	C1	M1	A1	N1	T1	E1	H1	
73	RVA/Human-wt/ZAF/UFS-NGS-MRC-DPRU1048/2008/G1P[8]	G1	I	P[8]	III	I1	R1	C1	M1	A1	N1	T1	E1	H1	
74	RVA/Human-wt/ZAF/UFS-NGS-MRC-DPRU1965/2008/G1P[8]	G1	I	P[8]	III	I1	R1	C1	M1	A1	N1	T1	E1	H1	
75	RVA/Human-wt/ZAF/UFS-NGS-MRC-DPRU1967/2008/G1P[8]	G1	I	P[8]	III	I1	R1	C1	M1	A1	N1	T1	E1	H1	
76	RVA/Human-wt/ZAF/UFS-NGS-MRC-DPRU1969/2008/G1P[8]	G1	I	P[8]	III	I1	R1	C1	M1	A1	N1	T1	E1	H1	
77	RVA/Human-wt/ZAF/UFS-NGS-MRC-DPRU1970/2008/G1P[8]	G1	I	P[8]	III	I1	R1	C1	M1	A1	N1	T1	E1	H1	
78	RVA/Human-wt/ZAF/UFS-NGS-MRC-DPRU1980/2008/G1P[8]	G1	II	P[8]	III	I1	R1	C1	M1	A1	N1	T1	E1	H1	
79	RVA/Human-wt/ZAF/UFS-NGS-MRC-DPRU2006/2008/G1P[8]	G1	I	P[8]	III	I1	R1	C1	M1	A1	N1	T1	E1	H1	
80	RVA/Human-wt/ZAF/UFS-NGS-MRC-DPRU136/2009/G1P[8]	G1	I	P[8]	III	I1	R1	C1	M1	A1	N1	T1	E1	H1	
81	RVA/Human-wt/ZAF/UFS-NGS-MRC-DPRU138/2009/G1P[8]	G1	II	P[8]	III	I1	R1	C1	M1	A1	N1	T1	E1	H1	
82	RVA/Human-wt/ZAF/UFS-NGS-MRC-DPRU139/2009/G1P[8]	G1	I	P[8]	III	I1	R1	C1	M1	A1	N1	T1	E1	H1	
83	RVA/Human-wt/ZAF/UFS-NGS-MRC-DPRU151/2009/G1P[8]	G1	II	P[8]	III	I1	R1	C1	M1	A1	N1	T1	E1	H1	
84	RVA/Human-wt/ZAF/UFS-NGS-MRC-DPRU157/2009/G1P[8]	G1	II	P[8]	III	I1	R1	C1	M1	A1	N1	T1	E1	H1	
85	RVA/Human-wt/ZAF/UFS-NGS-MRC-DPRU1049/2009/G1P[8]	G1	I	P[8]	III	I1	R1	C1	M1	A1	N1	T1	E1	H1	
86	RVA/Human-wt/ZAF/UFS-NGS-MRC-DPRU1093/2009/G1P[8]	G1	I	P[8]	III	I1	R1	C1	M1	A1	N1	T1	E1	H1	
87	RVA/Human-wt/ZAF/UFS-NGS-MRC-DPRU1190/2009/G1P[8]	G1	I	P[8]	III	I1	R1	C1	M1	A1	N1	T1	E1	H1	
88	RVA/Human-wt/ZAF/UFS-NGS-MRC-DPRU1234/2009/G1P[8]	G1	II	P[8]	III	I1	R1	C1	M1	A1	N1	T1	E1	H1	
89	RVA/Human-wt/ZAF/UFS-NGS-MRC-DPRU1271/2009/G1P[8]	G1	II	P[8]	III	I1	R1	C1	M1	A1	N1	T1	E1	H1	
90	RVA/Human-wt/ZAF/UFS-NGS-MRC-DPRU2280/2009/G1P[8]	G1	I	P[8]	III	I1	R1	C1	M1	A1	N1	T1	E1	H1	
91	RVA/Human-wt/ZAF/UFS-NGS-MRC-DPRU2291/2009/G1P[8]	G1	I	P[8]	III	I1	R1	C1	M1	A1	N1	T1	E1	H1	
92	RVA/Human-wt/ZAF/UFS-NGS-MRC-DPRU2299/2009/G1P[8]	G1	II	P[8]	III	I1	R1	C1	M1	A1	N1	T1	E1	H1	
	**Post-vaccine strains**														
93	RVA/Human-wt/ZAF/UFS-NGS-MRC-DPRU2021/2010/G1P[8]	G1	I	P[8]	III	I1	R1	C1	M1	A1	N1	T1	E1	H1	
94	RVA/Human-wt/ZAF/UFS-NGS-MRC-DPRU2250/2013/G1P[8]	G1	II	P[8]	I	I1	R1	C1	M1	A1	N1	T1	E1	H1	
95	RVA/Human-wt/ZAF/UFS-NGS-MRC-DPRU56/2014/G1P[8]	G1	II	P[8]	III	I1	R1	C1	M1	A1	N1	T1	E1	H1	
96	RVA/Human-wt/ZAF/UFS-NGS-MRC-DPRU65/2014/G1P[8]	G1	II	P[8]	III	I1	R1	C1	M1	A1	N1	T1	E1	H1	
97	RVA/Human-wt/ZAF/UFS-NGS-MRC-DPRU74/2014/G1P[8]	G1	II	P[8]	III	I1	R1	C1	M1	A1	N1	T1	E1	H1	

98	RVA/Human-wt/ZAF/UFS-NGS-MRC-DPRU143/2014/G1P[8]	G1	II	P[8]	III	I1	R1	C1	M1	A1	N1	T1	E1	H1	
99	RVA/Human-wt/ZAF/UFS-NGS-MRC-DPRU150/2014/G1P[8]	G1	II	P[8]	III	I1	R1	C1	M1	A1	N1	T1	E1	H1	
100	RVA/Human-wt/ZAF/UFS-NGS-MRC-DPRU4347/2015/G1P[8]	G1	II	P[8]	III	I1	R1	C1	M1	A1	N1	T1	E1	H1	
101	RVA/Human-wt/ZAF/UFS-NGS-MRC-DPRU4352/2015/G1P[8]	G1	II	P[8]	III	I1	R1	C1	M1	A1	N1	T1	E1	H1	
102	RVA/Human-wt/ZAF/UFS-NGS-MRC-DPRU4357/2015/G1P[8]	G1	II	P[8]	III	I1	R1	C1	M1	A1	N1	T1	E1	H1	
103	RVA/Human-wt/ZAF/UFS-NGS-MRC-DPRU15948/2017/G1P[8]	G1	II	P[8]	III	I1	R1	C1	M1	A1	N1	T1	E1	H1	
	**Acquired from GenBank**														
	**Pre-vaccine strains**														
104	RVA/Human-wt/ZAF/MRC-DPRU4418/2002/G1P[8]	G1	II	P[8]	III	I1	R1	C1	M1	A1	N1	T1	E1	H1	KJ752974
105	RVA/Human-wt/ZAF/MRC-DPRU1192/2002/G1P[8]	G1	II	P[8]	III	I1	R1	C1	M1	A1	N1	T1	E1	H1	KP752554
106	RVA/Human-wt/ZAF/MRC-DPRU6113/2002/G1P[8]	G1	II	P[8]	III	I1	R1	C1	M1	A1	N1	T1	E1	H1	KJ751795
107	RVA/Human-wt/ZAF/MRC-DPRU4381/2002/G1P[8]	G1	II	P[8]	III	I1	R1	C1	M1	A1	N1	T1	E1	H1	KJ752963
108	RVA/Human-wt/ZAF/MRC-DPRU400/2003/G1P[8]	G1	II	P[8]	III	I1	R1	C1	M1	A1	N1	T1	E1	H1	KJ752421
109	RVA/Human-wt/ZAF/MRC-DPRU2198/2003/G1P[8]	G1	II	P[8]	III	I1	R1	C1	M1	A1	N1	T1	E1	H1	KJ752333
110	RVA/Human-wt/ZAF/MRC-DPRU648/2003/G1P[8]	G1	II	P[8]	III	I1	R1	C1	M1	A1	N1	T1	E1	H1	KJ752524
111	RVA/Human-wt/ZAF/MRC-DPRU451/2003/G1P[8]	G1	II	P[8]	III	I1	R1	C1	M1	A1	N1	T1	E1	H1	KJ753506
112	RVA/Human-wt/ZAF/MRC-DPRU445/2003/G1P[8]	G1	II	P[8]	III	I1	R1	C1	M1	A1	N1	T1	E1	H1	KJ753770
113	RVA/Human-wt/ZAF/MRC-DPRU2218/2003/G1P[8]	G1	II	P[8]	III	I1	R1	C1	M1	A1	N1	T1	E1	H1	KJ752808
114	RVA/Human-wt/ZAF/MRC-DPRU566/2003/G1P[8]	G1	II	P[8]	III	I1	R1	C1	M1	A1	N1	T1	E1	H1	KJ751685
115	RVA/Human-wt/ZAF/MRC-DPRU539/2003/G1P[8]	G1	II	P[8]	III	I1	R1	C1	M1	A1	N1	T1	E1	H1	KJ753846
116	RVA/Human-wt/ZAF/MRC-DPRU426/2004/G1P[8]	G1	II	P[8]	III	I1	R1	C1	M1	A1	N1	T1	E1	H1	KJ752568
117	RVA/Human-wt/ZAF/MRC-DPRU1224/2004/G1P[8]	G1	II	P[8]	III	I1	R1	C1	M1	A1	N1	T1	E1	H1	KJ752874
118	RVA/Human-wt/ZAF/MRC-DPRU1285/2004/G1P[8]	G1	II	P[8]	III	I1	R1	C1	M1	A1	N1	T1	E1	H1	KJ753098
119	RVA/Human-wt/ZAF/MRC-DPRU1275/2004/G1P[8]	G1	II	P[8]	III	I1	R1	C1	M1	A1	N1	T1	E1	H1	KJ753242
120	RVA/Human-wt/ZAF/MRC-DPRU1264/2004/G1P[8]	G1	II	P[8]	III	I1	R1	C1	M1	A1	N1	T1	E1	H1	KJ753308
121	RVA/Human-wt/ZAF/MRC-DPRU1299/2004/G1P[8]	G1	II	P[8]	III	I1	R1	C1	M1	A1	N1	T1	E1	H1	KJ753587
122	RVA/Human-wt/ZAF/MRC-DPRU1283/2004/G1P[8]	G1	II	P[8]	III	I1	R1	C1	M1	A1	N1	T1	E1	H1	KJ753621
123	RVA/Human-wt/ZAF/MRC-DPRU1277/2004/G1P[8]	G1	II	P[8]	III	I1	R1	C1	M1	A1	N1	T1	E1	H1	KJ753679
124	RVA/Human-wt/ZAF/MRC-DPRU1280/2004/G1P[8]	G1	II	P[8]	III	I1	R1	C1	M1	A1	N1	T1	E1	H1	KJ753781
125	RVA/Human-wt/ZAF/MRC-DPRU822/2005/G1P[8]	G1	I	P[8]	III	I1	R1	C1	M1	A1	N1	T1	E1	H1	KJ751976
126	RVA/Human-wt/ZAF/MRC-DPRU2132/2005/G1P[8]	G1	I	P[8]	IV	I1	R1	C1	M1	A1	N1	T1	E1	H1	KJ753163
127	RVA/Human-wt/ZAF/MRC-DPRU2114/2005/G1P[8]	G1	I	P[8]	IV	I1	R1	C1	M1	A1	N1	T1	E1	H1	KJ752078
128	RVA/Human-wt/ZAF/MRC-DPRU799/2006/G1P[8]	G1	I	P[8]	III	I1	R1	C1	M1	A1	N1	T1	E1	H1	KJ752177
129	RVA/Human-wt/ZAF/MRC-DPRU789/2006/G1P[8]	G1	I	P[8]	III	I1	R1	C1	M1	A1	N1	T1	E1	H1	KJ752311
130	RVA/Human-wt/ZAF/MRC-DPRU748/2006/G1P[8]	G1	I	P[8]	III	I1	R1	C1	M1	A1	N1	T1	E1	H1	KJ753462
131	RVA/Human-wt/ZAF/MRC-DPRU839/2006/G1P[8]	G1	I	P[8]	III	I1	R1	C1	M1	A1	N1	T1	E1	H1	KJ753565
132	RVA/Human-wt/ZAF/MRC-DPRU757/2006/G1P[8]	G1	I	P[8]	III	I1	R1	C1	M1	A1	N1	T1	E1	H1	KP752839
133	RVA/Human-wt/ZAF/MRC-DPRU832/2006/G1P[8]	G1	I	P[8]	III	I1	R1	C1	M1	A1	N1	T1	E1	H1	KP752998
134	RVA/Human-wt/ZAF/MRC-DPRU901/2006/G1P[8]	G1	I	P[8]	III	I1	R1	C1	M1	A1	N1	T1	E1	H1	KP752587
135	RVA/Human-wt/ZAF/MRC-DPRU840/2006/G1P[8]	G1	I	P[8]	III	I1	R1	C1	M1	A1	N1	T1	E1	H1	KJ753152
136	RVA/Human-wt/ZAF/MRC-DPRU803/2006/G1P[8]	G1	I	P[8]	III	I1	R1	C1	M1	A1	N1	T1	E1	H1	KJ752895
137	RVA/Human-wt/ZAF/MRC-DPRU1840/2007/G1P[8]	G1	I	P[8]	IV	I1	R1	C1	M1	A1	N1	T1	E1	H1	KJ753384
138	RVA/Human-wt/ZAF/MRC-DPRU1327/2007/G1P[8]	G1	I	P[8]	III	I1	R1	C1	M1	A1	N1	T1	E1	H1	KJ753220
139	RVA/Human-wt/ZAF/MRC-DPRU1370/2007/G1P[8]	G1	I	P[8]	III	I1	R1	C1	M1	A1	N1	T1	E1	H1	KJ753065
140	RVA/Human-wt/ZAF/MRC-DPRU1350/2007/G1P[8]	G1	II	P[8]	III	I1	R1	C1	M1	A1	N1	T1	E1	H1	KJ753805
141	RVA/Human-wt/ZAF/MRC-DPRU1381/2007/G1P[8]	G1	I	P[8]	III	I1	R1	C1	M1	A1	N1	T1	E1	H1	KJ752700
142	RVA/Human-wt/ZAF/MRC-DPRU1960/2008/G1P[8]	G1	I	P[8]	III	I1	R1	C1	M1	A1	N1	T1	E1	H1	KJ751905
143	RVA/Human-wt/ZAF/MRC-DPRU1039/2008/G1P[8]	G1	I	P[8]	III	I1	R1	C1	M1	A1	N1	T1	E1	H1	KJ752199
144	RVA/Human-wt/ZAF/MRC-DPRU1052/2008/G1P[8]	G1	I	P[8]	III	I1	R1	C1	M1	A1	N1	T1	E1	H1	KJ752278
145	RVA/Human-wt/ZAF/MRC-DPRU2429/2008/G1P[8]	G1	I	P[8]	III	I1	R1	C1	M1	A1	N1	T1	E1	H1	KJ752841
146	RVA/Human-wt/ZAF/MRC-DPRU2125/2008/G1P[8]	G1	I	P[8]	III	I1	R1	C1	M1	A1	N1	T1	E1	H1	KP752893
147	RVA/Human-wt/ZAF/MRC-DPRU1270/2009/G1P[8]	G1	II	P[8]	III	I1	R1	C1	M1	A1	N1	T1	E1	H1	KJ751696
148	RVA/Human-wt/ZAF/MRC-DPRU1389/2009/G1P[8]	G1	II	P[8]	III	I1	R1	C1	M1	A1	N1	T1	E1	H1	KJ751839
149	RVA/Human-wt/ZAF/MRC-DPRU2801/2009/G1P[8]	G1	II	P[8]	III	I1	R1	C1	M1	A1	N1	T1	E1	H1	KJ753209
150	RVA/Human-wt/ZAF/MRC-DPRU150/2009/G1P[8]	G1	II	P[8]	III	I1	R1	C1	M1	A1	N1	T1	E1	H1	KJ753231
151	RVA/Human-wt/ZAF/MRC-DPRU155/2009/G1P[8]	G1	II	P[8]	III	I1	R1	C1	M1	A1	N1	T1	E1	H1	KJ753743
152	RVA/Human-wt/ZAF/MRC-DPRU2294/2009/G1P[8]	G1	II	P[8]	III	I1	R1	C1	M1	A1	N1	T1	E1	H1	KJ751751
153	RVA/Human-wt/ZAF/MRC-DPRU1269/2009/G1P[8]	G1	II	P[8]	III	I1	R1	C1	M1	A1	N1	T1	E1	H1	KJ753754
154	RVA/Human-wt/ZAF/MRC-DPRU2325/2009/G1P[8]	G1	I	P[8]	III	I1	R1	C1	M1	A1	N1	T1	E1	H1	KF636162
155	RVA/Human-wt/ZAF/MRC-DPRU2306/2009/G1P[8]	G1	I	P[8]	III	I1	R1	C1	M1	A1	N1	T1	E1	H1	KF636173
156	RVA/Human-wt/ZAF/MRC-DPRU2330/2009/G1P[8]	G1	I	P[8]	III	I1	R1	C1	M1	A1	N1	T1	E1	H1	KF636272
157	RVA/Human-wt/ZAF/MRC-DPRU1217/2009/G1P[8]	G1	I	P[8]	III	I1	R1	C1	M1	A1	N1	T1	E1	H1	KJ753451
158	RVA/Human-wt/ZAF/MRC-DPRU135/2009/G1P[8]	G1	I	P[8]	III	I1	R1	C1	M1	A1	N1	T1	E1	H1	KJ753087
159	RVA/Human-wt/ZAF/MRC-DPRU1923/2009/G1P[8]	G1	I	P[8]	III	I1	R1	C1	M1	A1	N1	T1	E1	H1	KP752987
	**Post-vaccine strains**														
160	RVA/Human-wt/ZAF/MRC-DPRU2030/2010/G1P[8]	G1	I	P[8]	III	I1	R1	C1	M1	A1	N1	T1	E1	H1	KF636206
161	RVA/Human-wt/ZAF/MRC-DPRU2035/2010/G1P[8]	G1	I	P[8]	III	I1	R1	C1	M1	A1	N1	T1	E1	H1	KF636239
162	RVA/Human-wt/ZAF/MRC-DPRU2052/2010/G1P[8]	G1	I	P[8]	III	I1	R1	C1	M1	A1	N1	T1	E1	H1	KF636283
163	RVA/Human-wt/ZAF/MRC-DPRU1506/2010/G1P[8]	G1	I	P[8]	III	I1	R1	C1	M1	A1	N1	T1	E1	H1	KJ753198
164	RVA/Human-wt/ZAF/MRC-DPRU1544/2010/G1P[8]	G1	I	P[8]	III	I1	R1	C1	M1	A1	N1	T1	E1	H1	KF636217
165	RVA/Human-wt/ZAF/MRC-DPRU2061/2010/G1P[8]	G1	I	P[8]	III	I1	R1	C1	M1	A1	N1	T1	E1	H1	KF636184
166	RVA/Human-wt/ZAF/MRC-DPRU1492/2010/G1P[8]	G1	I	P[8]	III	I1	R1	C1	M1	A1	N1	T1	E1	H1	KF636195
167	RVA/Human-wt/ZAF/MRC-DPRU83/2011/G1P[8]	G1	II	P[8]	III	I1	R1	C1	M1	A1	N1	T1	E1	H1	KJ752985
168	RVA/Human-wt/ZAF/MRC-DPRU6954/2011/G1P[8]	G1	I	P[8]	III	I1	R1	C1	M1	A1	N1	T1	E1	H1	KJ752020
169	RVA/Human-wt/ZAF/MRC-DPRU120/2011/G1P[8]	G1	I	P[8]	III	I1	R1	C1	M1	A1	N1	T1	E1	H1	KJ752155
170	RVA/Human-wt/ZAF/MRC-DPRU121/2011/G1P[8]	G1	I	P[8]	III	I1	R1	C1	M1	A1	N1	T1	E1	H1	KJ752590
171	RVA/Human-wt/ZAF/MRC-DPRU4079-11/2011/G1P[8]	G1	I	P[8]	III	I1	R1	C1	M1	A1	N1	T1	E1	H1	KJ753576
